# Stereoselective Lewis base catalyzed formal 1,3-dipolar cycloaddition of azomethine imines with mixed anhydrides†
†Electronic supplementary information (ESI) available: Detailed experimental procedures, and spectral data for all compounds, including scanned images of ^1^H and ^13^C NMR spectra. CCDC 1019046. For ESI and crystallographic data in CIF or other electronic format see DOI: 10.1039/c4sc02612h


**DOI:** 10.1039/c4sc02612h

**Published:** 2014-11-19

**Authors:** Lena Hesping, Anup Biswas, Constantin G. Daniliuc, Christian Mück-Lichtenfeld, Armido Studer

**Affiliations:** a Organisch-Chemisches Institut , Westfälische Wilhelms-Universität Münster Corrensstrasse 40 , 48149 Münster , Germany . Email: studer@uni-muenster.de ; http://www.uni-muenster.de/Chemie.oc/studer/ ; Fax: +49-251-83-36523 ; Tel: +49-251-83-33291

## Abstract

Stereoselective synthesis of pyrazolidinones *via* dipolar cycloaddition of azomethine imines with active esters under Lewis base catalysis is presented. The active esters are readily generated *in situ* from the corresponding acids. Products, which are obtained with excellent diastereocontrol and high enantioselectivity, contain along with the pyrazolidinone core also the tetrahydroisoquinoline structural motif. Theoretical studies give insight into the mechanism of the formal cycloaddition reaction.

## Abstract 2

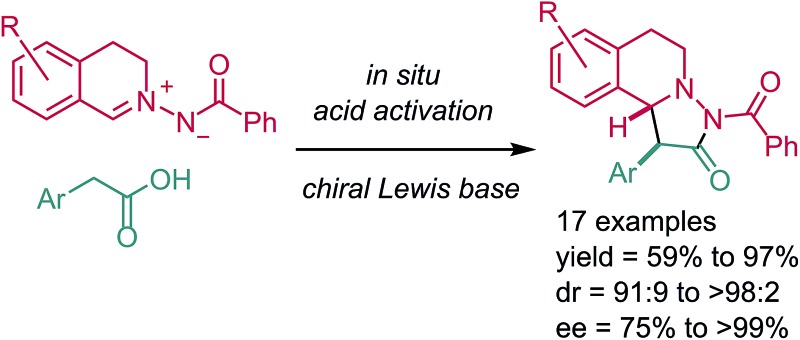
Pyrazolidinones with a tetrahydroisoquinoline core are obtained with excellent diastereocontrol and high enantioselectivity. Theoretical studies give insight on the reaction mechanism.

## Introduction

**Fig. 1:**
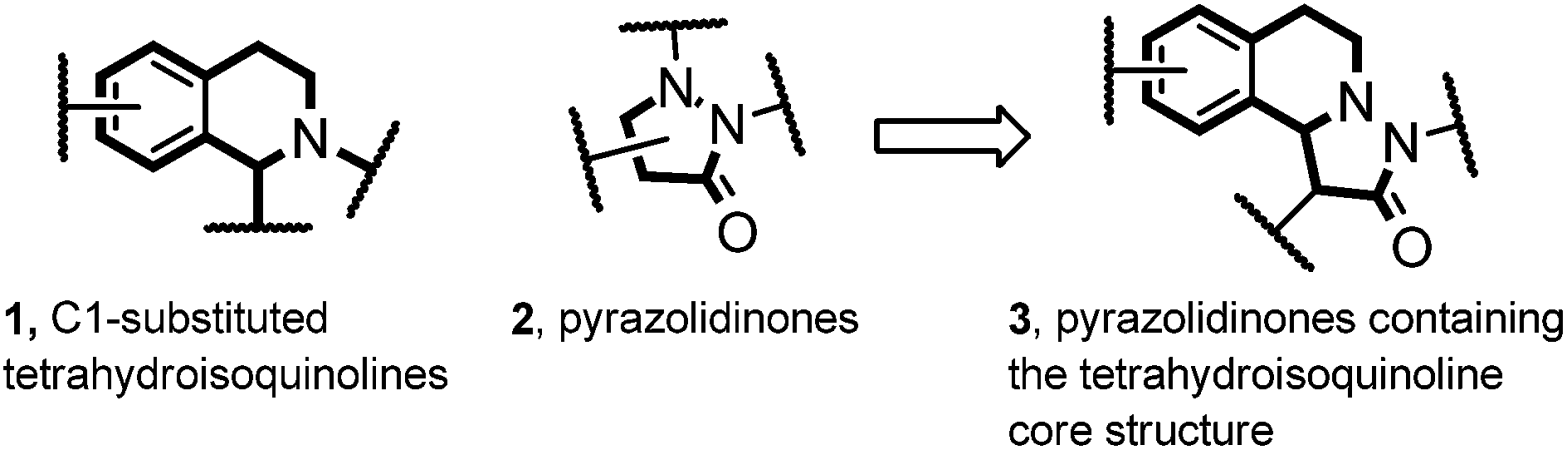
Compounds **3** combining structural features of both **1** and **2**.

Many natural products with interesting biological activity are based on the C1-substituted tetrahydroisoquinoline core **1**.^1^ It is obvious that many synthetic methods have been developed for the stereoselective construction of this important core structure.^2^ Pyrazolidinones **2** are also interesting compounds which have found wide applications in different fields. This structural motif can be found in dyes, in pharmaceuticals and agriculturally relevant compounds.^3^ Pyrazolidinones of type **3** combine the structural features of both **1** and **2** and should therefore be valuable compounds which have so far not been intensively investigated (Fig. 1).^4^ Herein we present a novel straightforward method for the stereoselective synthesis of compounds of type **3***via* 1,3-dipolar cycloaddition of azomethine imines with mixed anhydrides.

## Results and discussion

### Experimental studies

The intermolecular [2 + 3] cycloaddition of azomethine imines with alkynes is known for more than 45 years,^5^ and an enantioselective version was developed by Fu *et al.* using Cu-catalysis.^6^ Chen,^7^ Sibi,8*a* Maruoka^9^ and others8b–d disclosed enantioselective cycloadditions of azomethine imines with electron-poor alkenes and the corresponding enantioselective dipolar cycloaddition with electron-rich alkenes was reported by Leighton^10^ and Maruoka.^11^ Very recently, Wang *et al.* published amine-catalyzed enantioselective 1,3-dipolar cycloadditions of aldehydes to *C*,*N*-cyclic azomethine imines, where intermediately generated electron-rich enamines are the actual dipolarophiles.^12^

**Fig. 2:**
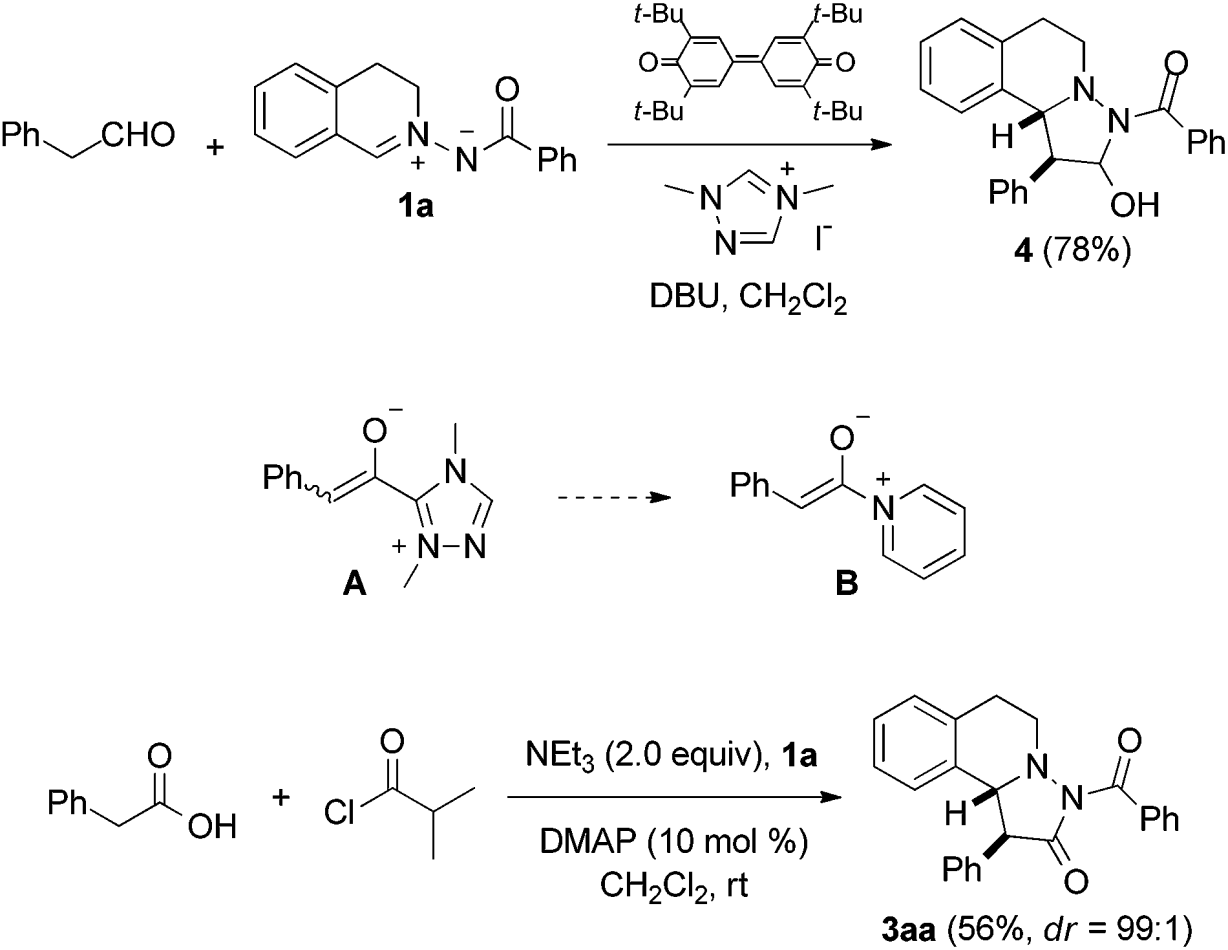
Transformation of **1a** to either **4** or **3aa** under different conditions.

As a continuation of our studies on oxidative carbene catalysis,^13^ we decided to investigate the reaction of aliphatic aldehydes with azomethine imines in the presence of a N-heterocyclic carbene (NHC) under oxidative conditions as a novel method for the preparation of compounds of type **3**. Disappointingly, we found that the azomethine imine **1a** reacted with phenylacetaldehyde in the presence of triazole pre-catalyst and DBU under oxidative conditions to cycloadduct **4** (ref. 14) (Fig. 2). The targeted pyrazolidinone **3aa** was not identified.

The reaction of phenylacetaldehyde with the NHC, oxidation to the acylazolium ion and subsequent enolization^15^ to give an enolate of type **A** is obviously slower than direct reaction of phenylacetaldehyde with **1a** under the applied basic conditions. We therefore switched to enolates of type **B** as potential dipolarophiles for the [2 + 3] cycloaddition with **1a**. It is important to note that stereoselective reactions with enolates formally deriving from acylammonium or acylpyridinium ions have been intensively studied in asymmetric catalysis.^16^ However, the application of such enolates as dipolarophiles in the reaction with azomethine imines is unknown.

**Fig. 3:**
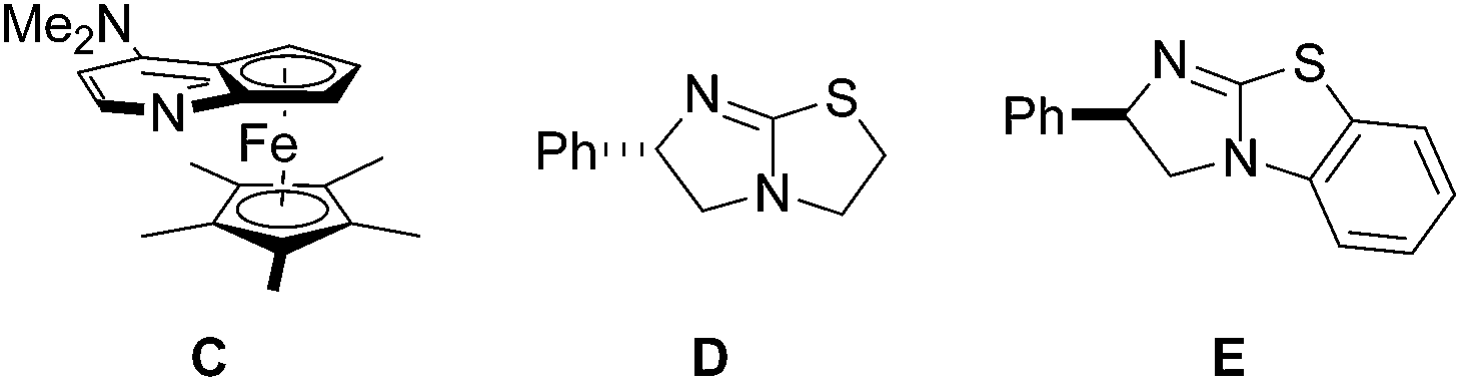
Chiral Lewis bases **C**, **D** and **E** used in this study.

Pleasingly, the mixed anhydride *in situ* generated from phenylacetic acid and isobutyric acid chloride in the presence of NEt_3_ reacted with **1a** and DMAP (10 mol%) to pyrazolidinone **3aa** which was isolated in 56% yield as a 99 : 1 mixture of diastereoisomers, as analyzed by HPLC.^17^,^18^ Encouraged by this result, we continued the studies by testing the chiral commercially available Lewis bases **C**,^19^**D**,^20^ and **E**,^20^ which have successfully been used in asymmetric catalysis (Fig. 3).^21^,^22^

**Fig. 4:**
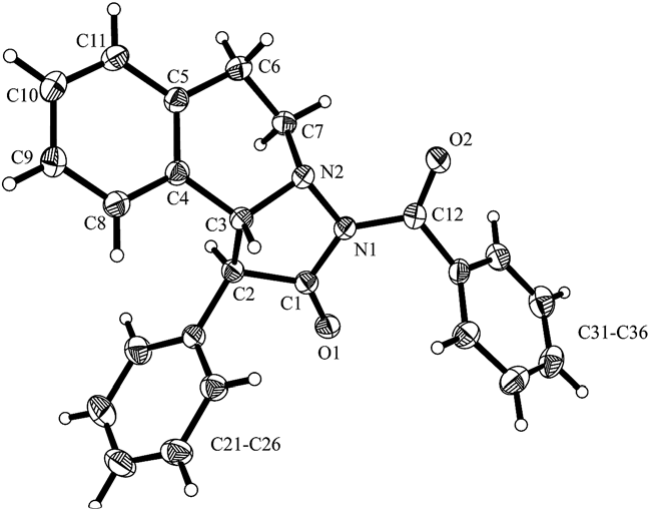
X-ray structure of pyrazolidinone **3aa**. Thermal ellipsoids are shown with 30% probability.

With catalyst **C**, **3aa** was obtained in moderate to good yield with high diastereoselectivity (95 : 5) but no or very low enantioselectivity by using *i*-Pr_2_NEt or Et_3_N as base (Table 1, no. 1, 2). Pleasingly, tetramisole **D** (10 mol%) provided **3aa** with significant ee (56%) but low diastereoselectivity (67 : 33) which was improved to 91 : 9 upon increasing the catalyst loading to 20 mol% (Table 1, no. 3, 4). Likely, back ground reaction, which is cycloaddition of the free ketene with the azomethine imine, competes at lower catalyst loading. This assumption is supported by the fact that the diastereoselectivity at lower catalyst loading was significantly lower (67 : 33 *versus* 91 : 9) and accordingly also the ee was lower. Enantioselectivity was determined by HPLC analysis (see ESI†). With phenylacetyl chloride as substrate, ee and dr were further improved; however, yield was very low in that case (Table 1, no. 5). As compared to **D**, catalyst **E** provided slightly improved selectivities (Table 1, no. 6, 7). From the experiment run with the acid chloride as a substrate it became obvious that the leaving group at the activated acid derivatives may play an important role on the selectivity. Therefore, we tested the *in situ* generated mixed anhydride derived from 2,4,6-trimethylbenzoyl chloride. Disappointingly, both diastereoselectivity and enantioselectivity decreased (Table 1, no. 8). However, the mixed anhydride formed from benzoyl chloride gave **3aa** with excellent ee (99%) and high diastereoselectivity (94 : 6) in good yield (Table 1, no. 9). We found that reaction works far more efficiently at room temperature without diminishing selectivity to a large extent and pyrazolidinone **3aa** was obtained in 95% yield, 94 : 6 diastereoselectivity with 98% ee (Table 1, no. 10). The absolute and relative configuration of **3aa** were unambiguously assigned by X-ray analysis (Fig. 4).

**Table 1:** Reaction optimization

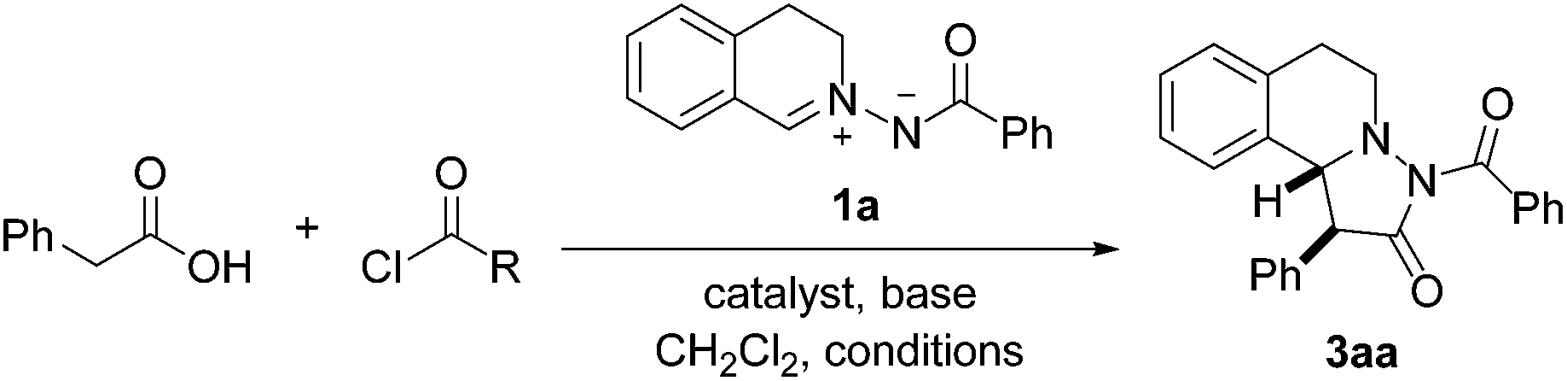							
No.	R	Cat. (mol%)	Base	Temp (°C)/Time (h)	dr	ee (%)	Yield (%)
1	*i*-Pr	**C** (10)	Et_3_N^*a*^	rt/16	95 : 5	6	46
2	*i*-Pr	**C** (10)	*i*-Pr_2_NEt^*a*^	rt/14	95 : 5	*rac*	71
3	*i*-Pr	**D** (10)	*i*-Pr_2_NEt^*b*^	0/23	67 : 33	56^*c*^	90
4	*i*-Pr	**D** (20)	*i*-Pr_2_NEt^*a*^	0/23	91 : 9	76^*c*^	74
5	—^*d*^	**D** (10)	*i*-Pr_2_NEt^*b*^	0/24	99 : 1	92^*c*^	12
6	*i*-Pr	**E** (10)	*i*-Pr_2_NEt^*a*^	0/43	95 : 5	64	64
7	*i*-Pr	**E** (10)	*i*-Pr_2_NEt^*b*^	rt/43	94 : 6	84	86
8	Mes	**E** (10)	*i*-Pr_2_NEt^*b*^	5/24	83 : 17	32	33
9	Ph	**E** (10)	*i*-Pr_2_NEt^*b*^	5/24	94 : 6	99	68
10	Ph	**E** (10)	*i*-Pr_2_NEt^*b*^	rt/16	94 : 6	98	95

^*a*^With 2.1 equiv. base.

^*b*^With 1.1 equiv. base.

^*c*^Other enantiomer formed as major isomer.

^*d*^Phenylacetyl chloride used as substrate.

**Fig. 5:**
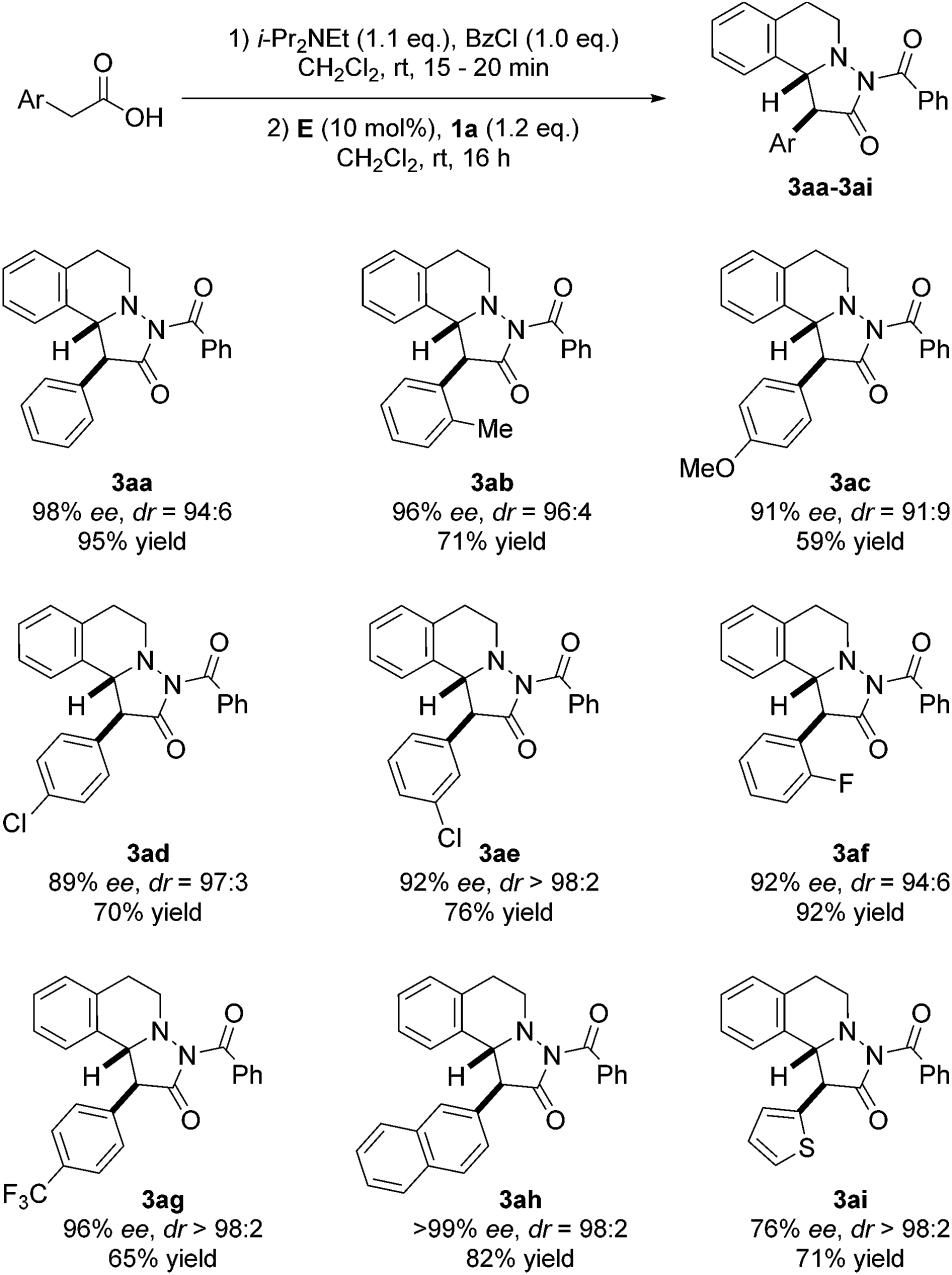
Variation of the acetic acid component.

**Fig. 6:**
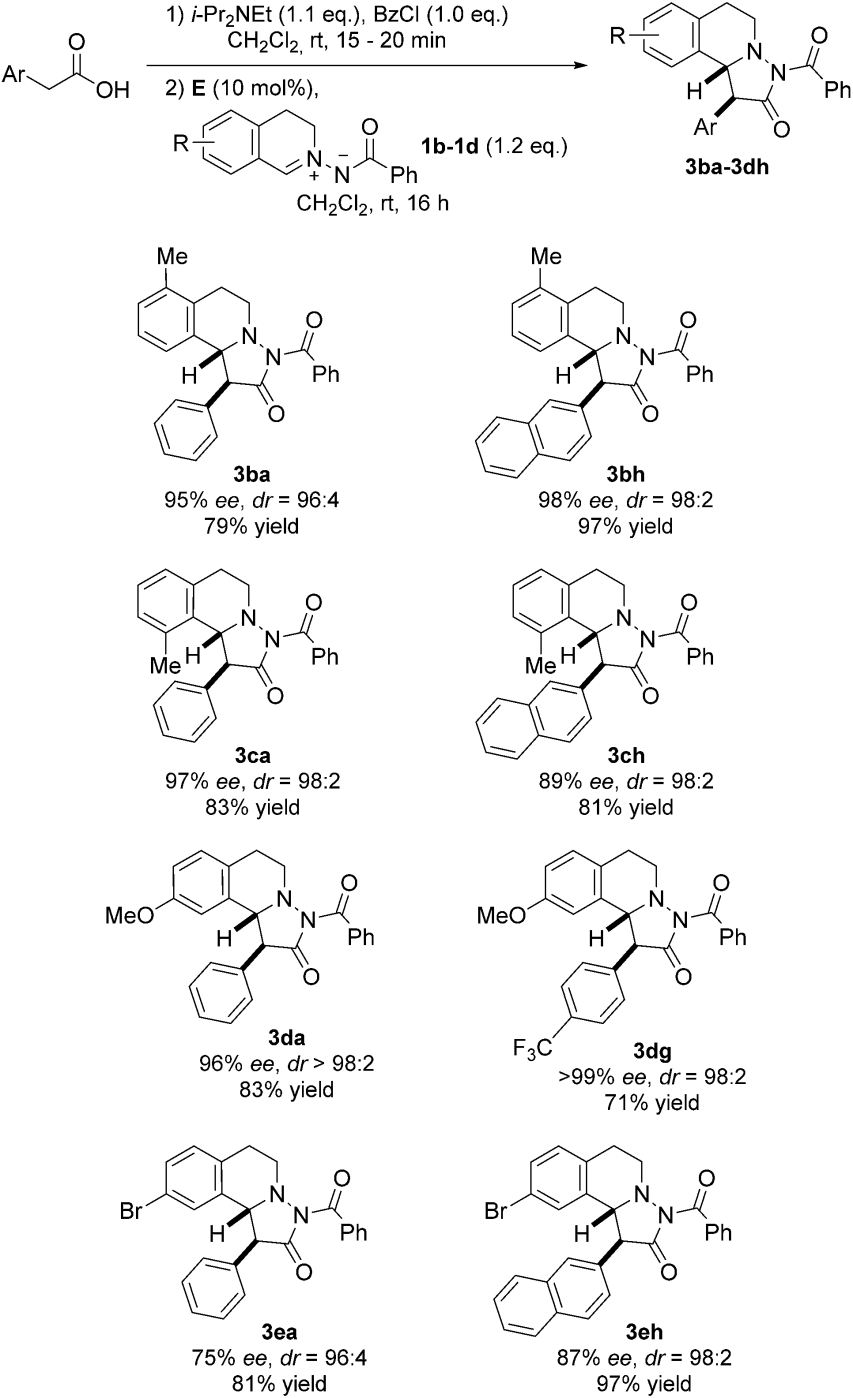
Variation of the azomethine imine and the acetic acid component.

With optimized conditions in hand, we tested scope and limitation of the stereoselective cycloaddition by varying the acid component and also the azomethine imine (Fig. 5 and 6). All azomethine imines used in this study were prepared according to literature procedures (see ESI†). Phenylacetic acids with both electron-donating and electron-withdrawing groups were tolerated to afford cycloaddition products **3ac–3ag** in good to high yields and high enantio- and diastereoselectivity. Furthermore, the sterically demanding *o*-tolylacetic acid could be utilized furnishing **3ab** with high ee and dr. Excellent selectivity and good yield were achieved with 2-naphthylacetic acid as the substrate (product **3ah**, >99% ee, dr = 98 : 2, 82% yield). Additionally, the heteroaromatic substrate thiopheneacetic acid worked well providing cycloadduct **3ai** in good yield and excellent diastereoselectivity, albeit with slightly lower ee.

Next, differently substituted azomethine imines were tested in the reaction with various acetic acid derivatives (Fig. 6). Good to high yields were obtained by using 7-bromo and 7-methoxy-substituted azomethine imines **1d** and **1e** (see **3da–3eh**). The electron-rich azomethine imine **1d** provided significantly higher ee as compared to the ee obtained with the Br-derivative **1e**. Along these lines, 5-methyl-substituted azomethine imine **1b** afforded pyrazolidinones **3ba** and **3bh** in high selectivities and good to excellent yields. Moreover, the sterically hindered 8-methyl-substituted azomethine imine **1c** was a good substrate and cycloadducts **3ca** and **3ch** were obtained in good to excellent selectivities with good yields.

All attempts to conduct the cycloaddition of the chiral ammonium enolate derived from phenylacetic acid with *in situ* generated *C*,*N*-cyclic azomethine imines according to the elegant work of Maruoka *et al.*^9^ failed. Moreover, the *N*,*N*-cyclic azomethine imine 2-benzylidene-5-oxopyrazolidin-2-ium-1-ide, often used in dipolar cycloaddtions,^6^–^8^ did not react with the *in situ* generated ammonium enolate under the tested conditions.

**Fig. 7:**
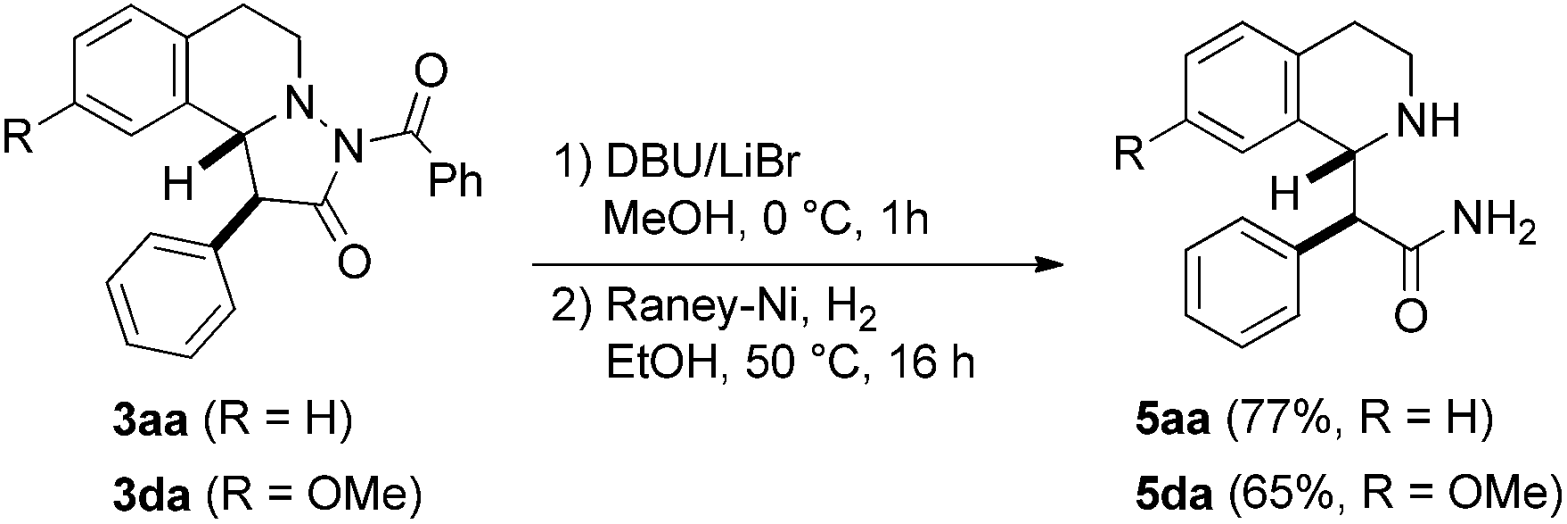
Reductive cleavage of the N–N bond for preparation of β-amino acid derivatives.

To show the value of pyrazolidinones as building blocks in synthesis, we tested a first follow-up reaction. To this end, *N*-benzoyl deprotection in pyrazolidinones **3aa** and **3da** was achieved with DBU/LiBr^23^ in MeOH to give the corresponding NH-pyrazolidinones. N–N bond cleavage by reduction with Raney-Ni/H_2_ eventually provided stereospecifically the tetrahydroisoquinolines **5aa** and **5da** in good yields (Fig. 7). These β-amino acid derivatives might be valuable for preparation of β-peptides.^24^

### Theoretical studies of the mechanism

**Fig. 8:**
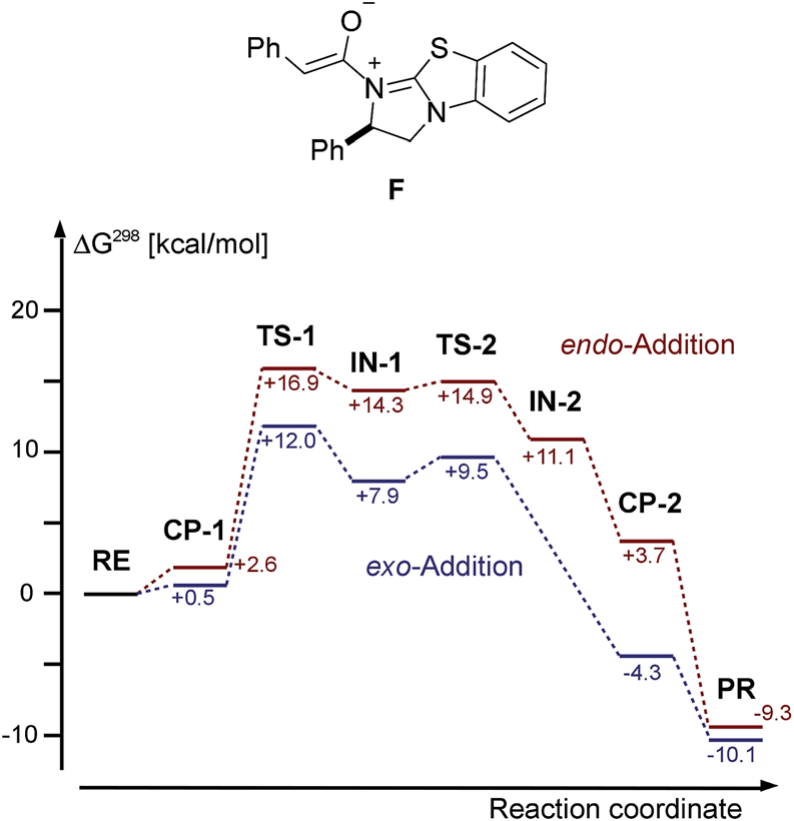
Chemical structure of enolate **F** and DFT calculated (TPSS-D3/def2-TZVP + COSMO (CH_2_Cl_2_) free energies of the cycloaddition of **F** and **1a**. **RE** = **1a**/**F**, **PR** = **3aa**/**E**.

In our DFT study of the mechanism of the cycloaddition reaction, we used TPSS-D3/def2-TZVP and an implicit solvation model (COSMO), for details, see ESI.† We chose the reaction of intermediate **F** deriving from phenylacetic acid and catalyst **E** with azomethine imine **1a** as our model system. The free energies, including solvation energies in CH_2_Cl_2_ and thermodynamic corrections at 298 K starting from the enolate **F** and **1a** are given in Fig. 8.

**Fig. 9:**
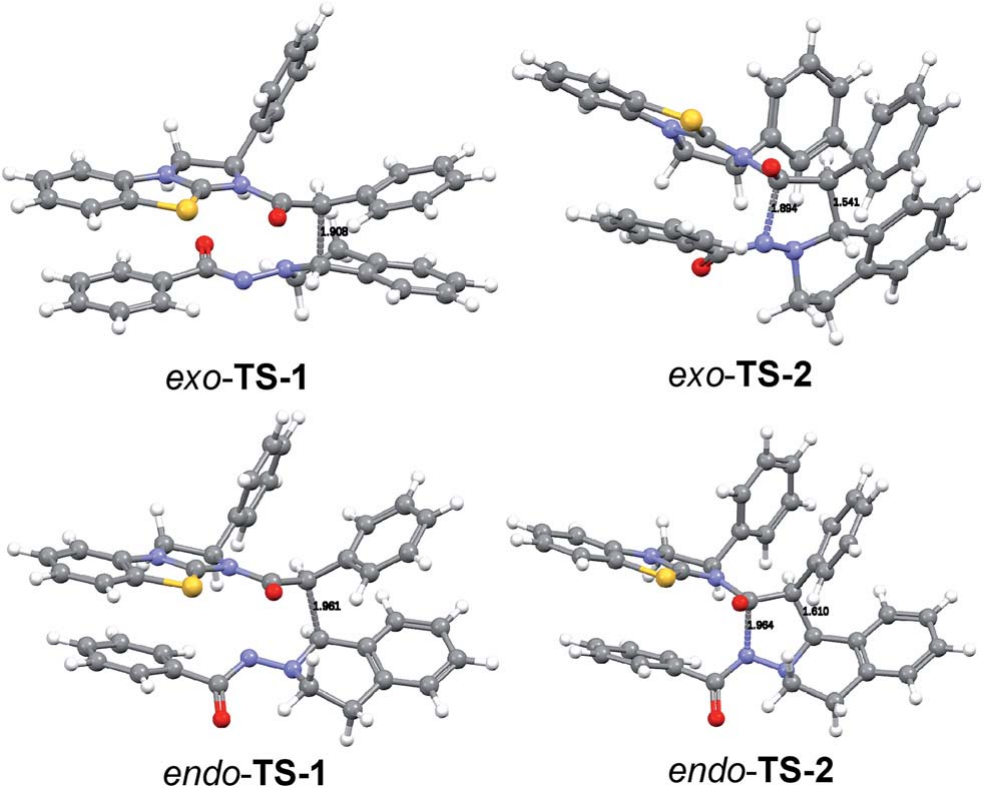
DFT calculated transition structures in the cycloaddition of **F** and **1a**.

We were not able to locate a transition structure of synchronous formation of both bonds (C–C and C–N) of the product (**3aa**). The mechanism proceeds stepwise with C–C bond formation as the first step (**TS-1**), leading to an intermediate (**IN-1**), which subsequently forms the second C–N bond with a very low barrier. Thus, the first step is determining the rate and the stereochemical outcome of the process. The preferred orientation of the two reactants in the pre-reactive complex **CP-1** and for **TS-1** is *exo* (see Fig. 9), in accordance with the observed diastereoselectivity of the reaction. Moreover, the absolute stereochemistry obtained in the calculations agreed with the stereochemistry observed in the experiment. In the *exo* reaction, we could not identify a tetrahedral intermediate **IN-2** as for *endo*, the catalyst **E** is released instantaneously upon formation of the C–N bond of **3aa**. The breakup of the product complex **CP-2** releases product **3aa**, of which the *trans*-diastereoisomer is also thermodynamically the more stable one.

## Conclusion

In summary, the chiral Lewis base benzotetramisole (**E**) catalyzes the highly enantio- and diastereoselective formation of complex pyrazilidinones with a tetrahydroisoquinoline core by 1,3-dipolar cycloaddition of *C*,*N*-cyclic azomethine imines and activated arylacetic acid derivatives. Reactions proceed in high yields with good to excellent diastereo- and enantioselectivity. Reductive N–N bond cleavage and imide hydrolysis provide β-aminoamides. DFT studies reveal a stepwise mechanism with the formation of the C–C bond as the first step which determines the rate and stereochemical outcome of the formal dipolar cycloaddition. The following C–N bond formation occurs with a low barrier.

## Supplementary Material

Supplementary information

Crystal structure data
