# Supplementary material for: Stereoselective Lewis base catalyzed formal 1,3-dipolar cycloaddition of azomethine imines with mixed anhydrides
Source: Chem Sci. 2014 Nov 19;6(2):1252–7. doi: 10.1039/c4sc02612h (PMC5811128; doi:10.1039/c4sc02612h)
Supplement: Supplementary file 1 [file SC-006-C4SC02612H-s001.pdf]

Electronic Supplementary Material (ESI) for Chemical Science.
This journal is © The Royal Society of Chemistry 2014

Supporting Information

Stereoselective Lewis Base Catalyzed 1,3-Dipolar Formal Cycloaddition of Azomethine Imines with Mixed Anhydrides

Lena Hesping, Anup Biswas, Constantin G. Daniliuc, Christian Mück-Lichtenfeld* and Armido Studer*

Fachbereich Chemie, Organisch-Chemisches Institut, Westfälische Wilhelms-Universität, Corrensstrasse 40, 48149 Münster, Germany
studer@uni-muenster.de

1. General information 1
2. Experimental procedures 2
   2.1 General procedure for asymmetric 1,3-dipolar formal cycloaddition of azomethine imines with mixed anhydrides (*GP*) 4
3. X-ray crystallographic data of pyrazolidinone 3aa 17
4. DFT calculations 18
5. HPLC chromatograms of cycloaddition products 22
6. ^{1}H-, ^{13}C-, and ^{19}F-NMR spectra 31
7. Literature 56

1. General information

All reactions involving air- or moisture-sensitive reagents or intermediates were carried out in heat gun-dried glassware under an argon atmosphere and were performed using standard *Schlenk* techniques. Dichloromethane was freshly distilled from P_2O_5. Toluene (99.8%, *AcroSeal® ExtraDry over Molecular Sieves*) and methanol (99.8%, *AcroSeal® ExtraDry over Molecular Sieves*), were purchased from *Acros* and used as received. Unless otherwise noted, all other chemicals were purchased from *Sigma Aldrich*, *Acros Organics*, *ABCR*, *Alfa Aesar*, *Fluka* or *TCI* and were used as received. Solvents for extraction and flash chromatography were distilled before use.

^{1}H-NMR spectra were recorded on a *Bruker DPX 300* (300 MHz) spectrometer. **^{13}C-NMR** spectra were recorded on a *Bruker DPX 300* (75 MHz), a *Bruker AV400* (101 MHz) or an *Agilent DD2 600* (151 MHz) spectrometer. **^{19}F-NMR** spectra were recorded on a *Bruker DPX 300* (282 MHz) or an *Agilent DD2 600* (564 MHz) spectrometer. Chemical shifts δ in ppm are referenced to the solvent residual peak ($CDCl_3$, ^{1}H: δ = 7.26 ppm, ^{13}C: δ = 77.2 ppm) and to an external standard ($CFCl_3$: δ = 0 ppm) for ^{19}F-NMR spectra. Peak multiplicities are given as following: *s*, singlet; *d*, doublet; *t*, triplet; *q*, quartet; *m*, multiplet. **HRMS ESI** (*m*/*z*) spectra were recorded on a *Bruker MicroTof*. **Melting points** (MP) were determined on a *SMP 10 apparatus* (*Stuart Scientific*) and are uncorrected. **IR** spectra were recorded on a *Digilab Varian 4000* or *3100 FT-IR Excalibur Series* with a *MKII Golden Gate Single Reflection* ATR unit. IR signals are described as *w* (weak), *m* (middle), *s* (strong), *br* (broad) in cm^{-1}. **Optical rotation** measurements were performed on a *Perkin-Elmer Polarimeter 341* or a *Jasco P2000 Polarimeter*. Sample concentrations are given in g/100 mL. **HPLC** measurements were performed on a *Hewlett Packard Series 1100* HPLC using UV detection (210 nm, 230 nm, 250 nm, or 260 nm). Separation was performed on a *Chiralpak® AD-H* (0.46 cm × 25 cm, *Daicel Chemical Industries, Ltd.*) or a *Chiralpak® IA* (0.46 cm × 25 cm, *Daicel Chemical Industries, Ltd.*) column. Thin layer chromatography (**TLC**) was carried out on *Merck* silica gel 60 F_{254} plates; detection with UV light or by dipping into a solution of $KMnO_4$ (1.5 g) and $NaHCO_3$ (5.0 g) in H_2O (400 mL) followed by heating. Flash column chromatography (**FC**) was carried out on *Merck* silica gel 60 (40-63 µm) with an argon pressure of about 0.5-1.0 bar.

Diastereomeric ratios were determined by ^{1}H-NMR analysis of the crude mixtures.

2. Experimental procedures

Azomethine imines were prepared according to literature procedures.[1]

The physical data of benzoyl(3,4-dihydroisoquinolin-2-ium-2-yl)amide (**1a**), benzoyl(5-methyl-3,4-dihydroisoquinolin-2-ium-2-yl)amide (**1b**), benzoyl(8-methyl-3,4-dihydroisoquinolin-2-ium-2-yl)amide (**1c**) and benzoyl(7-bromo-3,4-dihydroisoquinolin-2-ium-2-yl)amide (**1e**) are in accordance with those described in the literature.[1a]

1,7-Dimethoxyisochroman

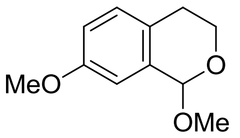

To a solution of DDQ (1.308 g, 5.762 mmol, 1.2 eq.) in CH_2Cl_2 (30 mL) was added anhydrous MeOH (0.24 mL, 5.9 mmol, 1.2 equiv.) and then 7-methoxyisochroman[1d] (791 mg, 4.82 mmol, 1.0 equiv.) at room temperature. The resulting dark green-blue solution was vigorously stirred at room temperature over 20 h and then quenched by addition of $NaHCO_3$ (aq. sat., 30 mL). The heterogeneous mixture was filtered through Celite® which was then rinsed with CH_2Cl_2 (20 mL). The aqueous layer was separated and extracted twice with CH_2Cl_2 (30 mL) and the combined organic layers were washed once with $NaHCO_3$ (aq. sat., 80 mL), once with brine (80 mL), dried over $MgSO_4$ and evaporated under reduced pressure. The crude material was purified by FC (P/EtOAc = 15/1) to give 1,7-dimethoxyisochroman (473 mg, 2.44 mmol, 51%) as a colorless oil.

IR (neat): 743*w*, 819*w*, 853*w*, 957*m*, 999*w*, 1050*s*, 1091*s*, 1188*w*, 1239*m*, 1274*m*, 1319*w*, 1349*w*, 1431*w*, 1466*w*, 1504*s*, 1615*w*, 2832*brw*, 2884*brw*, 2936*brw*. **^{1}H-NMR** (300 MHz, $CDCl_3$, 300 K): δ (ppm) = 7.04 (*d*, *J* = 8.4 Hz, 1H, $C_{arom}H$), 6.82 (*dd*, *J* = 8.4, 2.7 Hz, 1H, $C_{arom}H$), 6.77 (*d*, *J* = 2.7 Hz, 1H, $C_{arom}H$), 5.42 (*s*, 1H, CH), 4.09 (*td*, *J* = 11.6, 3.5 Hz, 1H, CH_2), 3.90 (*ddd*, *J* = 11.2, 5.9, 1.8 Hz, 1H, CH_2), 3.79 (*s*, 3H, CH_3), 3.55 (*s*, 3H, CH_3), 2.95 (*ddd*, *J* = 17.2, 11.9, 5.9 Hz, 1H, CH_2), 2.56 (*ddd*, *J* = 16.2, 3.6, 1.8 Hz, 1H, CH_2). **^{13}C-NMR** (75 MHz, $CDCl_3$, 300 K): δ (ppm) = 158.2 (C), 135.1 (C), 129.6 (CH), 126.2 (C), 115.4 (CH), 111.7 (CH), 98.0 (CH), 58.3 (CH_3), 55.5 (CH_2), 55.4 (CH_2), 27.4 (CH_3). **HRMS (ESI)** *m/z* = 217.0835 calcd. for $C_{11}H_{14}O_3Na^+$ $[M+Na]^+$; found: 217.0837.

2-(2-Bromoethyl)-5-methoxybenzaldehyde

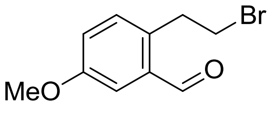

To a solution of 1,7-dimethoxyisochroman (473 mg, 2.44 mmol, 1.0 equiv.) in toluene (2.4 mL) were added tetrabutylammonium bromide (785 mg, 2.44 mmol, 1.0 equiv.) and trimethylsilyl bromide (0.64 mL, 4.8 mmol, 2.0 equiv.) at room temperature. The reaction tube was sealed with a screw cap and the reaction mixture was stirred at 80 °C. After 4.5 h, $NaHCO_3$ (aq. sat., 5 mL) was added, followed by extraction with EtOAc (10 mL). The combined organic layers were dried over $MgSO_4$ and concentrated *in vacuo*. FC (P/CH_2Cl_2 = 1/1) of the crude material afforded the desired benzaldehyde derivative (358 mg, 1.47 mmol, 60%) as a light brown oil.

IR (neat): 635*m*, 744*w*, 779*w*, 829*m*, 877*m*, 1035*s*, 1088*m*, 1267*s*, 1322*m*, 1428*m*, 1500*s*, 1572*m*, 1607*m*, 1690*s*, 2736*brw*, 2838*brw*, 2936*brw*. **^{1}H-NMR** (300 MHz, $CDCl_3$, 300 K): δ (ppm) = 10.14 (*s*, 1H, CHO), 7.34 (*d*, *J* = 2.8 Hz, 1H, $C_{arom}H$), 7.24 (*d*, *J* = 8.4 Hz, 1H, $C_{arom}H$), 7.09 (*dd*, *J* = 8.4, 2.8 Hz, 1H, $C_{arom}H$), 3.86 (*s*, 3H, CH_3), 3.61 – 3.45 (*m*, 4H, CH_2). **^{13}C-NMR** (75 MHz, $CDCl_3$, 300 K): δ (ppm) = 192.2 (CH), 159.2 (C), 134.9 (C), 133.3 (CH), 133.0 (C),

120.1 (CH), 117.6 (CH), 55.7 (CH_3), 35.3 (CH_2), 33.4 (CH_2). **HRMS (ESI)** *m/z* = 264.9835 calcd. for $C_{10}H_{11}BrO_2Na^+$ $[M+Na]^+$; found: 264.9841.

Benzoyl(7-methoxy-3,4-dihydroisoquinolin-2-ium-2-yl)amide (1d)

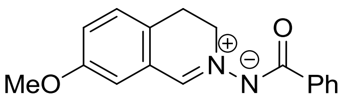

To a solution of 2-(2-bromoethyl)-5-methoxybenzaldehyde (328 mg, 1.35 mmol, 1.05 equiv.) in MeOH (2.7 mL) was added benzoylhydrazine (175 mg, 1.29 mmol, 1.0 equiv.) at room temperature. After the formation of a white suspension, the mixture was heated to reflux and stirred for an additional 1 h to give a clear solution. After cooling to room temperature, Et_3N (0.27 mL, 1.9 mmol, 1.5 equiv.) was added and the mixture was stirred for another 10 min at room temperature. Then water (5 mL) was added and the mixture was carefully stirred for 30 min to give a white precipitate. This solid material was washed with cold Et_2O and then dissolved in CH_2Cl_2 to give a yellow solution, which was dried over $MgSO_4$. Evaporation *in vacuo* afforded azomethine imine **1c** (319 mg, 1.14 mmol, 88%) as a yellow solid.

MP: 83 °C. **IR** (neat): 713*w*, 825*w*, 884*w*, 1031*m*, 1172*m*, 1291*s*, 1318*s*, 1447*w*, 1503*m*, 1551*m*, 1594*m*, 1663*w*, 2931*brw*, 3062*brw*. **^{1}H-NMR** (300 MHz, $CDCl_3$, 300 K): δ (ppm) = 9.68 (*s*, 1H, CHN), 8.13 – 8.04 (*m*, 2H, $C_{arom}H$), 7.43 – 7.32 (*m*, 3H, $C_{arom}H$), 7.16 (*d*, *J* = 8.3 Hz, 1H, $C_{arom}H$), 6.99 (*dd*, *J* = 8.3, 2.6 Hz, 1H, $C_{arom}H$), 6.93 (*d*, *J* = 2.6 Hz, 1H, $C_{arom}H$), 4.22 (*t*, *J* = 7.5 Hz, 2H, CH_2), 3.80 (*s*, 3H, CH_3), 3.13 (*t*, *J* = 7.6 Hz, 2H, CH_2). **^{13}C-NMR** (75 MHz, $CDCl_3$, 300 K): δ (ppm) = δ 170.7 (C), 159.2 (C), 147.3 (CH), 137.4 (C), 130.3 (CH), 128.7 (CH), 128.0 (2 × CH), 128.0 (2 × CH), 125.7 (C), 119.2 (CH), 114.1 (CH), 55.7 (CH_3), 55.5 (CH_2), 26.1 (CH_2). **HRMS (ESI)** *m/z* = 281.1285 calcd. for $C_{17}H_{16}N_2O_2H^+$ $[M+H]^+$; found: 281.1288.

2.1 General procedure for asymmetric 1,3-dipolar formal cycloaddition of azomethine imines with mixed anhydrides (*GP*)

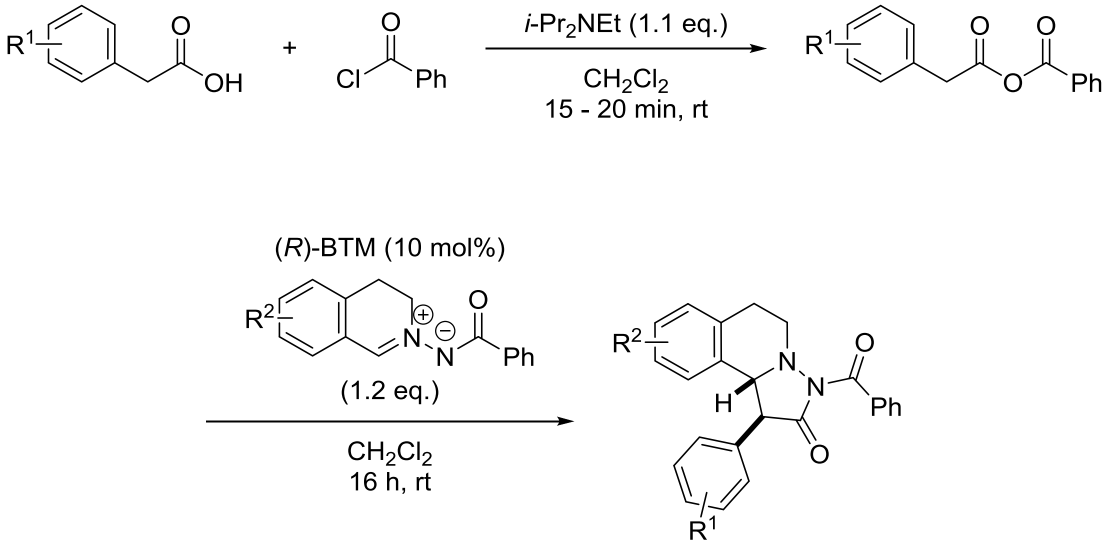

To a solution of the corresponding 2-phenylacetic acid derivative (1.0 eq.) in CH_2Cl_2 (0.1 M) was added *i*-Pr_2EtN (1.1 eq.) and then benzoyl chloride (1.0 eq.). The mixture was stirred for 15 – 20 min at room temperature to form the mixed anhydride. (*R*)-Benzotetramisole (10 mol-%) was added, followed by the azomethine imine (1.2 eq.) and the reaction mixture was stirred for 16 h at room temperature. The mixture was diluted with more CH_2Cl_2, washed with $NaHCO_3$ (aq. sat.) and water, dried over $MgSO_4$ and concentrated *in vacuo*. The crude material was purified by FC (P/MTBE) to give the desired pyrazolidinone.

(1*S*,10b*R*)-3-Benzoyl-1-phenyl-1,5,6,10b-tetrahydropyrazolo[5,1-*a*]isoquinolin-2(3*H*)-one (3aa)

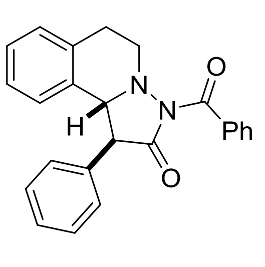

According to ***GP*** with 2-phenylacetic acid (14 mg, 0.10 mmol, 1.0 eq.), *i*-Pr_2EtN (18 µL, 0.11 mmol, 1.1 eq.), benzoyl chloride (12 µL, 0.10 mmol, 1.0 eq.), (*R*)-benzotetramisole (2.5 mg, 10 µmol, 10 mol-%) and benzoyl(3,4-dihydroisoquinolin-2-ium-2-yl)amide (**1a**) (30 mg, 0.12 mmol, 1.2 eq.). FC (P/MTBE = 2/1 → MTBE) afforded the desired pyrazolidinone **3aa** (35 mg, 95 µmol, 95%, *exo*/*endo* = 94/6) as a colorless solid.

$[\alpha]_D^{20}$ = +0.8° (c = 0.40 in CH_2Cl_2). **MP:** 191 °C. **IR** (neat): 634*m*, 663*s*, 695*s*, 732*s*, 757*m*, 770*m*, 800*s*, 862*w*, 886*w*, 956*m*, 1015*m*, 1061*m*, 1079*m*, 1120*m*, 1178*s*, 1215*s*, 1270*s*, 1290*s*, 1346*w*, 1369*w*, 1426*w*, 1455*m*, 1495*w*, 1583*w*, 1601*w*, 1684*s*, 1752*s*, 2339*w*, 2362*w*, 2853*brw*, 2925*brw*, 2958*brw*, 3030*brw*, 3036*brw* cm^{-1}. **^{1}H-NMR** (300 MHz, $CDCl_3$, 300 K): δ (ppm) = 7.77 – 7.68 (*m*, 2H, CH_{arom}), 7.59 – 7.48 (*m*, 1H, CH_{arom}), 7.48 – 7.32 (*m*, 5H, CH_{arom}), 7.29 – 7.13 (*m*, 4H, CH_{arom}), 6.98 (*m*, 1H, CH_{arom}), 6.34 (*d*, *J* = 7.8 Hz, 1H, CH_{arom}), 5.03 (*d*, *J* = 12.0 Hz, 1H, NCH), 4.16 (*d*, *J* = 12.0 Hz, 1H, CH), 3.82 – 3.65 (*m*, 1H, CH_2), 3.41 – 3.19 (*m*, 2H, CH_2), 3.01 – 2.79 (*m*, 1H, CH_2). **^{13}C-NMR** (75 MHz, $CDCl_3$, 300 K): δ (ppm) = 173.7 (C), 166.4 (C), 135.1 (C), 133.9 (C), 132.9 (C), 132.7 (C), 132.2 (CH), 129.7 (2 × CH), 129.3 (2 × CH), 129.0 (2 × CH), 128.7 (CH), 128.4 (CH), 128.1 (2 × CH), 127.7 (CH), 127.1 (CH), 126.2 (CH), 65.5 (CH), 55.7

(CH), 48.7 (CH_2), 29.0 (CH_2). **HRMS** (ESI): *m/z* = 369.1598 calcd. for $C_{24}H_{20}N_2O_2H^+$ $[M+H]^+$; found: 369.1597.

Enantiomeric excess (98% *ee*) was determined by chiral HPLC analysis (*CHIRALPAK® AD-H*, cyclohexane/2-propanol = 95/5, flow rate = 1.0 mL/min, t_r(major) = 23.1 min, t_r(minor) = 15.5 min).

(1*S*,10b*R*)-3-Benzoyl-1-(*o*-tolyl)-1,5,6,10b-tetrahydropyrazolo[5,1-*a*]isoquinolin-2(3*H*)-one (3ab)

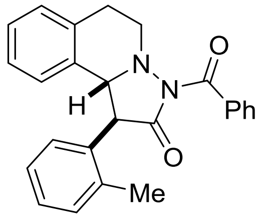

According to ***GP*** with 2-(*o*-tolyl)acetic acid (30 mg, 0.20 mmol, 1.0 eq.), *i*-Pr_2EtN (36 µL, 0.21 mmol, 1.1 eq.), benzoyl chloride (23 µL, 0.20 mmol, 1.0 eq.), (*R*)-benzotetramisole (5.0 mg, 20 µmol, 10 mol-%) and benzoyl(3,4-dihydroisoquinolin-2-ium-2-yl)amide (**1a**) (30 mg, 0.24 mmol, 1.2 eq.). FC (P/MTBE = 2/1) afforded the desired pyrazolidinone **3ab** (54 mg, 0.14 mmol, 71%, *exo*/*endo* = 96/4) as a light yellow solid.

$[\alpha]_D^{20}$ = +78.1° (c = 0.9 in $CHCl_3$). **MP:** 169 °C. **IR** (neat): 664*m*, 719*m*, 758*m*, 862*w*, 956*w*, 1015*w*, 1121*m*, 1197*s*, 1292*s*, 1347*m*, 1451*m*, 1493*m*, 1601*w*, 1685*s*, 1755*s*, 2857*brw*, 2929*brw*, 3028*brw*. **^{1}H-NMR** (300 MHz, $CDCl_3$, 300 K): δ (ppm) = 7.73 (*dt*, *J* = 7.0, 1.4 Hz, 2H, $C_{arom}H$), 7.56 – 7.48 (*m*, 1H, $C_{arom}H$), 7.48 – 7.38 (*m*, 2H, $C_{arom}H$), 7.37 – 7.23 (*m*, 3H, $C_{arom}H$), 7.23 – 7.16 (*m*, 3H, $C_{arom}H$), 7.02 – 6.91 (*m*, 1H, $C_{arom}H$), 6.35 (*d*, *J* = 7.8 Hz, 1H, $C_{arom}H$), 5.03 (*d*, *J* = 11.9 Hz, 1H, NCH), 4.47 (*d*, *J* = 11.9 Hz, 1H, CH), 3.81 – 3.67 (*m*, 1H, CH_2), 3.39 – 3.23 (*m*, 2H, CH_2), 2.99 – 2.83 (*m*, 1H, CH_2), 2.06 (*s*, 3H, CH_3). **^{13}C-NMR** (75 MHz, $CDCl_3$, 300 K): δ (ppm) = 173.9 (C), 166.4 (C), 137.8 (C), 134.0 (C), 133.7 (C), 132.7 (2 × C), 132.1 (CH), 131.0 (CH), 129.0 (2 × CH), 128.7 (2 × CH), 128.2 (CH), 128.1 (2 × CH), 127.7 (CH), 127.2 (CH), 126.5 (CH), 126.4 (CH), 65.5 (CH), 51.6 (CH), 48.7 (CH_2), 29.0 (CH_2), 20.1 (CH_3). **HRMS (ESI)** *m/z* = 405.1573 calcd. for $C_{25}H_{22}N_2O_2Na^+$ $[M+Na]^+$; found: 405.1567.

Enantiomeric excess (96% *ee*) was determined by chiral HPLC analysis (*CHIRALPAK® AD-H*, cyclohexane/2-propanol = 97/3, flow rate = 1.0 mL/min, t_r(major) = 26.0 min, t_r(minor) = 16.5 min).

(1*S*,10b*R*)-3-benzoyl-1-(4-methoxyphenyl)-1,5,6,10b-tetrahydropyrazolo[5,1-*a*]isoquinolin-2(3*H*)-one (3ac)

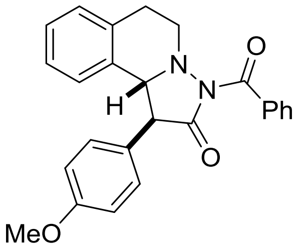

According to ***GP*** with 2-(4-methoxyphenyl)acetic acid (33 mg, 0.20 mmol, 1.0 eq.), *i*-Pr_2EtN (36 µL, 0.21 mmol, 1.1 eq.), benzoyl chloride (23 µL, 0.20 mmol, 1.0 eq.), (*R*)-benzotetramisole (5.0 mg, 20 µmol, 10 mol-%) and benzoyl(3,4-dihydroisoquinolin-2-ium-2-yl)amide (**1a**) (30 mg, 0.24 mmol, 1.2 eq.). FC (P/MTBE = 1.5/1) afforded the desired pyrazolidinone **3ac** (47 mg, 0.12 mmol, 59%, *exo*/*endo* = 91/9) as a light yellow solid.

$[\alpha]_D^{20}$ = +13.8° (c = 0.4 in $CHCl_3$). **MP:** 189 °C. **IR** (neat): 664*m*, 717*m*, 765*m*, 864*w*, 956*w*, 1029*m*, 1118*m*, 1180*s*, 1273*s*, 1347*w*, 1451*m*, 1515*s*, 1686*s*, 1754*s*, 1991*w*, 2839*brw*, 2929*brw*, 3034*brw*. **^{1}H-NMR** (300 MHz, $CDCl_3$, 300 K): δ (ppm) = 7.72 (*d*, *J* = 7.0 Hz, 2H, $C_{arom}H$), 7.56 – 7.47 (*m*, 1H, $C_{arom}H$), 7.46 – 7.37 (*m*, 2H, $C_{arom}H$), 7.22 – 7.11 (*m*, 4H, $C_{arom}H$), 7.03 – 6.91 (*m*,

3H, $C_{arom}H$), 6.39 (*d*, *J* = 7.8 Hz, 1H, $C_{arom}H$), 4.97 (*d*, *J* = 12.0 Hz, 1H, NCH), 4.10 (*d*, *J* = 12.0 Hz, 1H, CH), 3.84 (*s*, 3H, CH_3), 3.79 – 3.64 (*m*, 1H, CH_2), 3.40 – 3.16 (*m*, 2H, CH_2), 2.96 – 2.81 (*m*, 1H, CH_2). **^{13}C-NMR** (75 MHz, $CDCl_3$, 300 K): δ (ppm) = 174.0 (C), 166.4 (C), 159.7 (C), 134.0 (C), 132.9 (C), 132.2 (CH), 130.7 (2 × CH), 129.0 (2 × CH), 128.7 (CH), 128.6 (C), 128.1 (2 × CH), 127.7 (CH), 127.1 (CH), 127.0 (C), 126.2 (CH), 114.9 (2 × CH), 65.5 (CH), 55.5 (CH_3), 54.9 (CH), 48.7 (CH_2), 29.0 (CH_2). **HRMS (ESI)** *m/z* = 421.1523 calcd. for $C_{25}H_{22}N_2O_3Na^+$ $[M+Na]^+$; found: 421.1518.

Enantiomeric excess (91% *ee*) was determined by chiral HPLC analysis (*CHIRALPAK® IA*, cyclohexane/2-propanol = 96.5/3.5, flow rate = 0.3 mL/min, t_r(major) = 121.7 min, t_r(minor) = 105.0 min).

(1*S*,10b*R*)-3-Benzoyl-1-(4-chlorophenyl)-1,5,6,10b-tetrahydropyrazolo[5,1-*a*]isoquinolin-2(3*H*)-one (3ad)

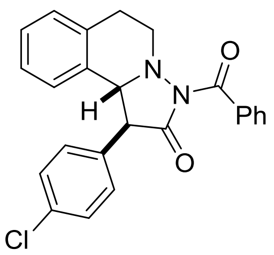

According to ***GP*** with 2-(4-chlorophenyl)acetic acid (34 mg, 0.20 mmol, 1.0 eq.), *i*-Pr_2EtN (36 µL, 0.21 mmol, 1.1 eq.), benzoyl chloride (23 µL, 0.20 mmol, 1.0 eq.), (*R*)-benzotetramisole (5.0 mg, 20 µmol, 10 mol-%) and benzoyl(3,4-dihydroisoquinolin-2-ium-2-yl)amide (**1a**) (30 mg, 0.24 mmol, 1.2 eq.). FC (P/MTBE = 2.5/1) afforded the desired pyrazolidinone **3ad** (56 mg, 0.14 mmol, 70%, *exo*/*endo* = 97/3) as a light yellow solid.

$[\alpha]_D^{20}$ = +16.0° (c = 1.0 in $CHCl_3$). **MP:** 173 °C. **IR** (neat): 641*w*, 664*m*, 712*m*, 791*m*, 955*w*, 1015*m*, 1091*m*, 1121*w*, 1182*m*, 1275*s*, 1346*w*, 1450*w*, 1493*m*, 1660*w*, 1686*m*, 1754*s*, 2848*brw*, 2927*brw*, 3064*brw*. **^{1}H-NMR** (300 MHz, $CDCl_3$, 300 K): δ (ppm) = 7.77 – 7.68 (*m*, 2H, $C_{arom}H$), 7.58 – 7.49 (*m*, 1H, $C_{arom}H$), 7.48 – 7.37 (*m*, 4H, $C_{arom}H$), 7.24 – 7.13 (*m*, 4H, $C_{arom}H$), 7.06 – 6.95 (*m*, 1H, $C_{arom}H$), 6.34 (*d*, *J* = 7.8 Hz, 1H, $C_{arom}H$), 4.97 (*d*, *J* = 12.0 Hz, 1H, NCH), 4.15 (*d*, *J* = 12.0 Hz, 1H, CH), 3.77 – 3.63 (*m*, 1H, CH_2), 3.38 – 3.21 (*m*, 2H, CH_2), 2.98 – 2.81 (*m*, 1H, CH_2). **^{13}C-NMR** (75 MHz, $CDCl_3$, 300 K): δ (ppm) = 173.2 (C), 166.3 (C), 134.5 (C), 133.8 (C), 133.6 (C), 132.9 (C), 132.4 (C), 132.3 (CH), 131.0 (2 × CH), 129.6 (2 × CH), 129.1 (2 × CH), 128.9 (CH), 128.1 (2 × CH), 127.9 (CH), 127.0 (CH), 126.3 (CH), 65.5 (CH), 55.1 (CH), 48.8 (CH_2), 28.9 (CH_2). **HRMS (ESI)** *m/z* = 425.1027 calcd. for $C_{24}H_{19}ClN_2O_2Na^+$ $[M+Na]^+$; found: 425.1019.

Enantiomeric excess (89% *ee*) was determined by chiral HPLC analysis (*CHIRALPAK® IA*, cyclohexane/2-propanol = 93/7, flow rate = 1.0 mL/min, t_r(major) = 22.7 min, t_r(minor) = 15.1 min).

(1*S*,10b*R*)-3-Benzoyl-1-(3-chlorophenyl)-1,5,6,10b-tetrahydropyrazolo[5,1-*a*]isoquinolin-2(3*H*)-one (3ae)

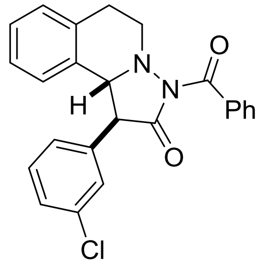

According to ***GP*** with 2-(3-chlorophenyl)acetic acid (34 mg, 0.20 mmol, 1.0 eq.), *i*-Pr_2EtN (36 µL, 0.21 mmol, 1.1 eq.), benzoyl chloride (23 µL, 0.20 mmol, 1.0 eq.), (*R*)-benzotetramisole (5.0 mg, 20 µmol, 10 mol-%) and benzoyl(3,4-dihydroisoquinolin-2-ium-2-yl)amide (**1a**) (30 mg, 0.24 mmol, 1.2 eq.). FC (P/MTBE = 1.5/1) afforded pyrazolidinone **3ae** (61 mg, 0.15 mmol, 76%, *exo*/*endo* > 98/2) as a light yellow solid.

$[\alpha]_D^{20}$ = +32.6° (c = 1.5 in $CHCl_3$). **MP:** 172 °C. **IR** (neat): 624*m*, 664*m*, 710*s*, 761*m*, 875*brw*, 958*w*, 1014*w*, 1082*w*, 1122*w*, 1186*m*, 1215*m*, 1274*s*, 1346*w*, 1451*w*, 1478*w*, 1575*w*, 1599*w*, 1686*s*, 1755*s*, 2009*w*, 2137*w*, 2169*w*, 2199*w*, 2361*brw*, 2848*brw*, 2927*brw*, 3073*brw*. **^{1}H-NMR** (300 MHz, $CDCl_3$, 300 K): δ (ppm) = 7.78 – 7.69 (*m*, 2H, $C_{arom}H$), 7.57 – 7.50 (*m*, 1H, $C_{arom}H$), 7.48 – 7.34 (*m*, 4H, $C_{arom}H$), 7.25 – 7.18 (*m*, 3H, $C_{arom}H$), 7.17 – 7.09 (*m*, 1H, $C_{arom}H$), 7.06 – 6.95 (*m*, 1H, $C_{arom}H$), 6.35 (*d*, *J* = 7.8 Hz, 1H, $C_{arom}H$), 5.00 (*d*, *J* = 12.0 Hz, 1H, NCH), 4.15 (*d*, *J* = 12.0 Hz, 1H, CH), 3.78 – 3.61 (*m*, 1H, CH_2), 3.38 – 3.21 (*m*, 2H, CH_2), 2.98 – 2.81 (*m*, 1H, CH_2). **^{13}C-NMR** (75 MHz, $CDCl_3$, 300 K): δ (ppm) = 173.0 (C), 166.3 (C), 137.0 (C), 135.2 (C), 133.7 (C), 132.8 (C), 132.3 (C, CH), 130.5 (CH), 129.9 (CH), 129.0 (2 × CH), 128.8 (CH), 128.7 (CH), 128.1 (2 × CH), 127.9 (CH), 127.8 (CH), 127.0 (CH), 126.3 (CH), 65.3 (CH), 55.3 (CH), 48.8 (CH_2), 28.9 (CH_2). **HRMS (ESI)** *m/z* = 425.1027 calcd. for $C_{24}H_{19}ClN_2O_2Na^+$ $[M+Na]^+$; found: 425.1025.

Enantiomeric excess (92% *ee*) was determined by chiral HPLC analysis (*CHIRALPAK® IA*, cyclohexane/2-propanol = 93/7, flow rate = 1.0 mL/min, t_r(major) = 17.6 min, t_r(minor) = 12.5 min).

(1*S*,10b*R*)-3-Benzoyl-1-(2-fluorophenyl)-1,5,6,10b-tetrahydropyrazolo[5,1-*a*]isoquinolin-2(3*H*)-one (3af)

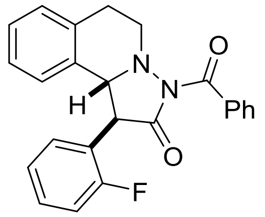

According to ***GP*** with 2-(2-fluorophenyl)acetic acid (62 mg, 0.40 mmol, 1.0 eq.), *i*-Pr_2EtN (72 µL, 0.42 mmol, 1.1 eq.), benzoyl chloride (46 µL, 0.40 mmol, 1.0 eq.), (*R*)-benzotetramisole (10 mg, 40 µmol, 10 mol-%) and benzoyl(3,4-dihydroisoquinolin-2-ium-2-yl)amide (**1a**) (120 mg, 0.479 mmol, 1.2 eq.). FC (P/MTBE = 5/1 → 2/1) afforded the desired pyrazolidinone **3af** (142 mg, 0.367 mmol, 92%, *exo*/*endo* = 94/6) as a colorless solid.

$[\alpha]_D^{20}$ = +46.0° (c = 0.9 in $CHCl_3$). **MP:** 182 °C. **IR** (neat): 663m, 727s, 801w, 910m, 955w, 1015w, 1121w, 1201s, 1275s, 1347w, 1452w, 1493m, 1061w, 1684s, 1755s, 2252w, 2852brw, 2927brw, 3063brw. **^{1}H-NMR** (300 MHz, $CDCl_3$, 300 K): δ (ppm) = 7.78 – 7.71 (*m*, 2H, $C_{arom}H$), 7.56 – 7.48 (*m*, 1H, $C_{arom}H$), 7.48 – 7.35 (*m*, 3H, $C_{arom}H$), 7.24 – 7.11 (*m*, 5H, $C_{arom}H$), 7.04 – 6.94 (*m*, 1H, $C_{arom}H$), 6.35 (*d*, *J* = 7.8 Hz, 1H, $C_{arom}H$), 5.13 (*d*, *J* = 11.8 Hz, 1H, NCH), 4.27 (*d*, *J* = 11.9 Hz, 1H, CH), 3.91 – 3.75 (*m*, 1H, CH_2), 3.43 – 3.20 (*m*, 2H, CH_2), 2.97 – 2.80 (*m*, 1H, CH_2). **^{13}C-NMR** (75 MHz, $CDCl_3$, 300 K): δ (ppm) = 172.8 (C), 166.5 (C), 161.3 (*d*, *J* = 247.4 Hz, C), 133.9 (C), 133.0 (C), 132.7 (C), 132.3 (*d*, *J* = 4.2 Hz, CH), 132.1 (CH), 130.4 (*d*, *J* = 8.5 Hz, CH), 129.0 (*d*, *J* = 1.3 Hz, 2 × CH), 128.8 (CH), 128.1 (2 × CH), 127.7 (CH), 126.9 (CH), 126.3 (CH), 124.9 (*d*, *J* = 3.4 Hz, CH), 122.5 (*d*, *J* = 13.3 Hz, C), 116.4 (*d*, *J* = 21.4 Hz CH), 63.3 (*d*, *J* = 2.3 Hz, CH), 51.6 (CH), 48.4 (CH_2), 29.0 (CH_2). **^{19}F-NMR** (282 MHz, CDCl3, 300 K) δ (ppm) = -114.2 (*s*, CF). **HRMS (ESI)** *m/z* = 409.1323 calcd. for $C_{24}H_{19}FN_2O_2Na^+$ $[M+Na]^+$; found: 409.1322.

Enantiomeric excess (92% *ee*) was determined by chiral HPLC analysis (*CHIRALPAK® AD-H*, cyclohexane/2-propanol = 96/4, flow rate = 0.5 mL/min, t_r(major) = 36.4 min, t_r(minor) = 32.9 min).

(1*S*,10b*R*)-3-Benzoyl-1-(4-(trifluoromethyl)phenyl)-1,5,6,10b-tetrahydropyrazolo[5,1-*a*]isoquinolin-2(3*H*)-one (3ag)

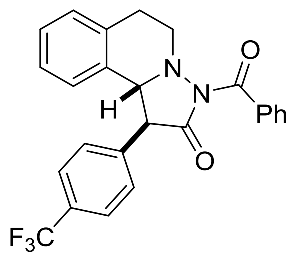

According to ***GP*** with 2-(4-(trifluoromethyl)phenyl)acetic acid (41 mg, 0.20 mmol, 1.0 eq.), *i*-Pr_2EtN (36 µL, 0.21 mmol, 1.1 eq.), benzoyl chloride (23 µL, 0.20 mmol, 1.0 eq.), (*R*)-benzotetramisole (5.0 mg, 20 µmol, 10 mol-%) and benzoyl(3,4-dihydroisoquinolin-2-ium-2-yl)amide (**1a**) (30 mg, 0.24 mmol, 1.2 eq.). FC (P/MTBE = 3/1) afforded the desired pyrazolidinone **3ag** (57 mg, 0.13 mmol, 65%, *exo*/*endo* > 98/2) as a colorless solid.

$[\alpha]_D^{20}$ = +87.8° (c = 0.5 in $CHCl_3$). **MP:** 200 °C. **IR** (neat): 664*m*, 698*m*, 764*m*, 795*w*, 841*w*, 950*w*, 1019*m*, 1069*s*, 1122*s*, 1168*s*, 1273*s*, 1326*s*, 1423*w*, 1451*w*, 1494*w*, 1619*w*, 1688*m*, 1756*s*, 2854*brw*, 2927*brw*, 3067*brw*. **^{1}H-NMR** (300 MHz, $CDCl_3$, 300 K): δ (ppm) = 7.76 – 7.67 (*m*, 4H, $C_{arom}H$), 7.58 – 7.50 (*m*, 1H, $C_{arom}H$), 7.47 – 7.34 (*m*, 4H, $C_{arom}H$), 7.24 – 7.19 (*m*, 2H, $C_{arom}H$), 7.05 – 6.96 (*m*, 1H, $C_{arom}H$), 6.29 (*d*, *J* = 7.8 Hz, 1H, $C_{arom}H$), 5.03 (*d*, *J* = 11.9 Hz, 1H, NCH), 4.25 (*d*, *J* = 11.9 Hz, 1H, CH), 3.79 – 3.64 (*m*, 1H, CH_2), 3.39 – 3.23 (*m*, 2H, CH_2), 3.00 – 2.82 (*m*, 1H, CH_2). **^{13}C-NMR** (151 MHz, $CDCl_3$, 300 K): δ (ppm) = 173.0 (C), 166.2 (C), 139.1 (C), 133.6 (C), 132.9 (C), 132.4 (CH), 132.2 (C), 130.7 (*q*, *J* = 32.7 Hz, C), 130.2 (2 × CH), 129.0 (2 × CH), 128.9 (CH), 128.1 (2 × CH), 128.0 (CH), 126.9 (CH), 126.4 (CH), 126.3 (*q*, *J* = 3.8 Hz, 2 × CH), 124.1 (*q*, *J* = 272.2 Hz, C), 65.5 (CH), 55.5 (CH), 48.8 (CH_2), 28.9 (CH_2). **^{19}F-NMR** (564 MHz, CDCl3, 300 K) δ (ppm) = -62.7 (*s*, CF_3). **HRMS (ESI)** *m/z* = 459.1291 calcd. for $C_{25}H_{19}F_3N_2O_2Na^+$ $[M+Na]^+$; found: 459.1291.

Enantiomeric excess (96% *ee*) was determined by chiral HPLC analysis (*CHIRALPAK® AD-H*, cyclohexane/2-propanol = 91/9, flow rate = 1.0 mL/min, t_r(major) = 16.8 min, t_r(minor) = 12.1 min).

(1*S*,10b*R*)-3-Benzoyl-1-(naphthalen-2-yl)-1,5,6,10b-tetrahydropyrazolo[5,1-*a*]isoquinolin-2(3*H*)-one (3ah)

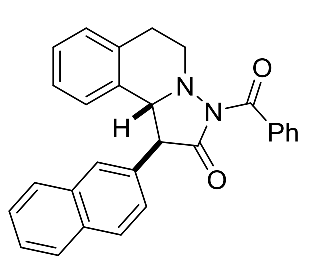

According to ***GP*** with 2-(naphthalen-2-yl)acetic acid (37 mg, 0.20 mmol, 1.0 eq.), *i*-Pr_2EtN (36 µL, 0.21 mmol, 1.1 eq.), benzoyl chloride (23 µL, 0.20 mmol, 1.0 eq.), (*R*)-benzotetramisole (5.0 mg, 20 µmol, 10 mol-%) and benzoyl(3,4-dihydroisoquinolin-2-ium-2-yl)amide (**1a**) (30 mg, 0.24 mmol, 1.2 eq.). FC (P/MTBE = 4/1) afforded the desired pyrazolidinone **3ah** (69 mg, 0.16 mmol, 82%, *exo*/*endo* = 98/2) as a yellow solid.

$[\alpha]_D^{20}$ = -1.1° (c = 0.9 in $CHCl_3$). **MP:** 159 °C. **IR** (neat): 664*m*, 697*m*, 761*m*, 807*m*, 861*w*, 946*w*, 1015*w*, 1061*w*, 1119*w*, 1193*s*, 1276*s*, 1346*w*, 1450*w*, 1493*w*, 1601*w*, 1686*s*, 1755*s*, 2854*brw*, 2924*brw*, 3055*brw*. **^{1}H-NMR** (300 MHz, $CDCl_3$, 300 K): δ (ppm) = 7.95 (*d*, *J* = 8.5 Hz, 1H, $C_{arom}H$), 7.92 – 7.86 (*m*, 1H, $C_{arom}H$), 7.85 – 7.74 (*m*, 3H, $C_{arom}H$), 7.70 (*d*, *J* = 1.7 Hz, 1H, $C_{arom}H$), 7.58 – 7.49 (*m*, 3H, $C_{arom}H$), 7.48 – 7.37 (*m*, 3H, $C_{arom}H$), 7.25 – 7.15 (*m*, 2H, $C_{arom}H$), 6.90 (*ddd*, *J* = 8.3, 6.3, 2.4 Hz, 1H, $C_{arom}H$), 6.32 (*d*, *J* = 7.7 Hz, 1H, $C_{arom}H$), 5.18 (*d*, *J* = 12.0 Hz, 1H, NCH), 4.35 (*d*, *J* = 12.0 Hz, 1H, CH), 3.82 – 3.67 (*m*, 1H, CH_2), 3.43 – 3.25 (*m*, 2H, CH_2), 3.00 – 2.84 (*m*, 1H, CH_2). **^{13}C-NMR** (75 MHz, $CDCl_3$, 300 K): δ (ppm) = 173.6 (C), 166.4 (C), 133.9 (C), 133.6 (C), 133.2 (C), 132.8 (C), 132.7 (C), 132.3 (C), 132.2 (CH), 129.6 (CH), 129.4

(CH), 129.1 (2 × CH), 128.7 (CH), 128.0 (2 × CH), 128.0 (CH), 127.9 (CH), 127.7 (CH), 127.1 (CH), 126.6 (CH), 126.6 (CH), 126.3 (CH), 126.2 (CH), 65.2 (CH), 55.8 (CH), 48.8 (CH_2), 29.0 (CH_2). **HRMS (ESI)** *m/z* = 441.1573 calcd. for $C_{28}H_{22}N_2O_2Na^+$ $[M+Na]^+$; found: 441.1564.

Enantiomeric excess (> 99% *ee*) was determined by chiral HPLC analysis (*CHIRALPAK® AD-H*, cyclohexane/2-propanol = 96.5/3.5, flow rate = 1.0 mL/min, t_r(major) = 31.9 min, t_r(minor) = 28.0 min).

(1*S*,10b*R*)-3-Benzoyl-1-(thiophen-2-yl)-1,5,6,10b-tetrahydropyrazolo[5,1-*a*]isoquinolin-2(3*H*)-one (3ai)

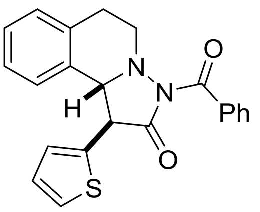

According to ***GP*** with 2-(thiophen-2-yl)acetic acid (57 mg, 0.40 mmol, 1.0 eq.), *i*-Pr_2EtN (72 µL, 0.42 mmol, 1.1 eq.), benzoyl chloride (46 µL, 0.40 mmol, 1.0 eq.), (*R*)-benzotetramisole (10 mg, 40 µmol, 10 mol-%) and benzoyl(3,4-dihydroisoquinolin-2-ium-2-yl)amide (**1a**) (120 mg, 0.479 mmol, 1.2 eq.). FC (P/MTBE = 2/1) afforded the desired pyrazolidinone **3ai** (107 mg, 0.286 mmol, 71%, *exo*/*endo* > 98/2) as a light brown solid.

$[\alpha]_D^{20}$ = +6.7° (c = 1.0 in $CHCl_3$). **MP:** 167 °C. **IR** (neat): 698*m*, 859*w*, 913*w*, 945*w*, 1016*w*, 1121*w*, 1220*m*, 1278*s*, 1344*w*, 1450*w*, 1493*w*, 1600*w*, 1688*s*, 1754*s*, 1935*w*, 1991*w*, 2073*w*, 2254*w*, 2481*w*, 2853*brw*, 2925*brw*, 3063*brw*. **^{1}H-NMR** (300 MHz, $CDCl_3$, 300 K): δ (ppm) = 7.75 – 7.67 (*m*, 2H, $C_{arom}H$), 7.56 – 7.48 (*m*, 1H, $C_{arom}H$), 7.46 – 7.37 (*m*, 3H, $C_{arom}H$), 7.25 – 7.17 (*m*, 2H, $C_{arom}H$), 7.11 – 6.99 (*m*, 3H, $C_{arom}H$), 6.61 (*d*, *J* = 7.8 Hz, 1H, $C_{arom}H$), 5.05 (*d*, *J* = 11.9 Hz, 1H, NCH), 4.45 (*d*, *J* = 11.9 Hz, 1H, CH), 3.75 (*ddd*, *J* = 9.9, 4.3, 2.3 Hz, 1H, CH_2), 3.40 – 3.16 (*m*, 2H, CH_2), 2.89 (*dt*, *J* = 15.2, 2.3 Hz, 1H, CH_2). **^{13}C-NMR** (75 MHz, $CDCl_3$, 300 K): δ (ppm) = 172.3 (C), 166.3 (C), 136.8 (C), 133.5 (C), 132.7 (C), 132.4 (C), 132.2 (CH), 128.9 (2 × CH), 128.6 (CH), 128.3 (CH), 128.0 (2 × CH), 127.8 (CH), 127.5 (CH), 126.8 (CH), 126.3 (2 × CH), 65.8 (CH), 50.9 (CH), 48.8 (CH_2), 28.8 (CH_2). **HRMS (ESI)** *m/z* = 397.0981 calcd. for $C_{22}H_{18}N_2O_2SNa^+$ $[M+Na]^+$; found: 397.0978.

Enantiomeric excess (76% *ee*) was determined by chiral HPLC analysis (*CHIRALPAK® AD-H*, cyclohexane/2-propanol = 94/6, flow rate = 1.0 mL/min, t_r(major) = 15.0 min, t_r(minor) = 12.9 min).

(1*S*,10b*R*)-3-Benzoyl-7-methyl-1-phenyl-1,5,6,10b-tetrahydropyrazolo[5,1-*a*]isoquinolin-2(3*H*)-one (3ba)

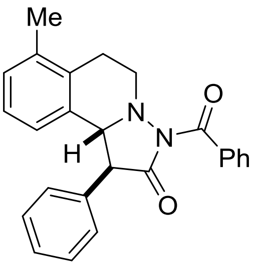

According to ***GP*** with 2-phenylacetic acid (54 mg, 0.40 mmol, 1.0 eq.), *i*-Pr_2EtN (75 µL, 0.44 mmol, 1.1 eq.), benzoyl chloride (46 µL, 0.40 mmol, 1.0 eq.), (*R*)-benzotetramisole (10 mg, 40 µmol, 10 mol-%) and benzoyl(5-methyl-3,4-dihydroisoquinolin-2-ium-2-yl)amide (**1b**) (126 mg, 0.477 mmol, 1.2 eq.). FC (P/MTBE = 2/1) afforded the desired pyrazolidinone **3ba** (121 mg, 0.316 mmol, 79%, *exo*/*endo* = 96/4) as a light yellow solid.

$[\alpha]_D^{20}$ = +39.8° (c = 1.0 in $CHCl_3$). **MP:** 189 °C. **IR** (neat): 664*m*, 704*s*, 781*w*, 953*w*, 1019*w*, 1082*w*, 1132*w*, 1182*m*, 1214*m*, 1285*s*, 1347*w*, 1468*w*, 1600*w*, 1686*s*, 1755*s*, 2927*brw*. **^{1}H-NMR** (300 MHz, $CDCl_3$, 300 K): δ (ppm) = 7.77 – 7.70 (*m*, 2H, $C_{arom}H$), 7.56 – 7.49 (*m*, 1H, $C_{arom}H$), 7.48 – 7.34 (*m*, 5H, $C_{arom}H$), 7.26 – 7.21 (*m*, 2H, $C_{arom}H$), 7.07 (*d*, *J* = 7.4 Hz, 1H, $C_{arom}H$), 6.89 (*t*,

J = 7.6 Hz, 1H, $C_{arom}H$), 6.18 (*d*, *J* = 7.8 Hz, 1H, $C_{arom}H$), 5.00 (*d*, *J* = 11.9 Hz, 1H, NCH), 4.18 (*d*, *J* = 11.9 Hz, 1H, CH), 3.77 (*ddd*, *J* = 10.2, 5.3, 2.3 Hz, 1H, CH_2), 3.28 (*ddd*, *J* = 11.9, 10.2, 3.7 Hz, 1H, CH_2), 3.07 (*ddd*, *J* = 17.1, 11.8, 5.3 Hz, 1H, CH_2), 2.89 (*ddd*, *J* = 16.8, 3.4, 1.3 Hz, 1H, CH_2), 2.28 (*s*, 3H, CH_3). **^{13}C-NMR** (75 MHz, $CDCl_3$, 300 K): δ (ppm) = 173.9 (C), 166.4 (C), 136.4 (C), 135.3 (C), 133.9 (C), 132.6 (C), 132.2 (CH), 131.5 (C), 129.7 (2 × CH), 129.3 (2 × CH), 129.1 (2 × CH), 129.0 (CH), 128.3 (CH), 128.1 (2 × CH), 126.0 (CH), 125.0 (CH), 65.8 (CH), 55.6 (CH), 48.6 (CH_2), 26.5 (CH_2), 19.6 (CH_3). **HRMS (ESI)** *m/z* = 405.1573 calcd. for $C_{25}H_{22}N_2O_2Na^+$ $[M+Na]^+$; found: 405.1571.

Enantiomeric excess (95% *ee*) was determined by chiral HPLC analysis (*CHIRALPAK® AD-H*, cyclohexane/2-propanol = 90/10, flow rate = 1.0 mL/min, t_r(major) = 8.7 min, t_r(minor) = 10.3 min).

(1*S*,10b*R*)-3-Benzoyl-7-methyl-1-(naphthalen-2-yl)-1,5,6,10b-tetrahydropyrazolo[5,1-*a*]isoquinolin-2(3*H*)-one (3bh)

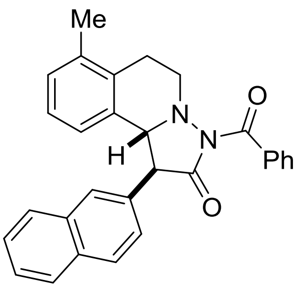

According to ***GP*** with 2-(naphthalen-2-yl)acetic acid (37 mg, 0.20 mmol, 1.0 eq.), *i*-Pr_2EtN (37 µL, 0.22 mmol, 1.1 eq.), benzoyl chloride (23 µL, 0.20 mmol, 1.0 eq.), (*R*)-benzotetramisole (5.0 mg, 20 µmol, 10 mol-%) and benzoyl(5-methyl-3,4-dihydroisoquinolin-2-ium-2-yl)amide (**1b**) (63 mg, 0.24 mmol, 1.2 eq.). FC (P/MTBE = 2/1 → 1/2) afforded the desired pyrazolidinone **3bh** (84 mg, 0.19 mmol, 97%, *exo*/*endo* = 98/2) as a colorless solid.

$[\alpha]_D^{20}$ = -5.1° (c = 0.5 in $CHCl_3$). **MP:** 208 °C. **IR** (neat): 628*w*, 642*w*, 664*m*, 695*m*, 712*m*, 748*s*, 807*w*, 859*w*, 948*w*, 1018*w*, 1081*w*, 1134*w*, 1201*m*, 1283*s*, 1343*w*, 1373*w*, 1448*w*, 1468*w*, 1510*w*, 1600*w*, 1682*m*, 1754*m*, 2337*brw*, 2363*brw*, 2854*brw*, 2924*brw*, 3021*brw*, 3057*brw*. **^{1}H-NMR** (300 MHz, $CDCl_3$, 300 K): δ (ppm) = 7.95 (*d*, *J* = 8.5 Hz, 1H, $C_{arom}H$), 7.92 – 7.86 (*m*, 1H, $C_{arom}H$), 7.85 – 7.75 (*m*, 3H, $C_{arom}H$), 7.71 (*d*, *J* = 1.7 Hz, 1H, $C_{arom}H$), 7.58 – 7.48 (*m*, 3H, $C_{arom}H$), 7.48 – 7.38 (*m*, 3H, $C_{arom}H$), 7.06 (*d*, *J* = 7.4 Hz, 1H, $C_{arom}H$), 6.82 (*t*, *J* = 7.6 Hz, 1H, $C_{arom}H$), 6.17 (*d*, *J* = 7.8 Hz, 1H, $C_{arom}H$), 5.15 (*d*, *J* = 11.9 Hz, 1H, NCH), 4.38 (*d*, *J* = 11.9 Hz, 1H, CH), 3.80 (*ddd*, *J* = 10.2, 5.3, 2.2 Hz, 1H, CH_2), 3.34 (*ddd*, *J* = 11.8, 10.3, 3.7 Hz, 1H, CH_2), 3.09 (*ddd*, *J* = 17.1, 11.8, 5.3 Hz, 1H, CH_2), 2.97 – 2.86 (*m*, 1H, CH_2), 2.29 (*s*, 3H, CH_3). **^{13}C-NMR** (75 MHz, $CDCl_3$, 300 K): δ (ppm) = 173.8 (C), 166.4 (C), 136.4 (C), 133.9 (C), 133.6 (C), 133.2 (C), 132.6 (C), 132.5 (C), 132.2 (CH), 131.4 (C), 129.7 (CH), 129.3 (CH), 129.1 (2 × CH), 129.0 (CH), 128.1 (2 × CH), 128.0 (CH), 127.9 (CH), 126.6 (CH), 126.5 (CH), 126.3 (CH), 126.0 (CH), 125.0 (CH), 65.5 (CH), 55.8 (CH), 48.7 (CH_2), 26.5 (CH_2), 19.6 (CH_3). **HRMS (ESI)** *m/z* = 455.1730 calcd. for $C_{29}H_{24}N_2O_2Na^+$ $[M+Na]^+$; found: 455.1721.

Enantiomeric excess (98% *ee*) was determined by chiral HPLC analysis (*CHIRALPAK® AD-H*, cyclohexane/2-propanol = 90/10, flow rate = 1.0 mL/min, t_r(major) = 9.6 min, t_r(minor) = 14.2 min).

(1*S*,10b*R*)-3-Benzoyl-10-methyl-1-phenyl-1,5,6,10b-tetrahydropyrazolo[5,1-*a*]isoquinolin-2(3*H*)-one (3ca)

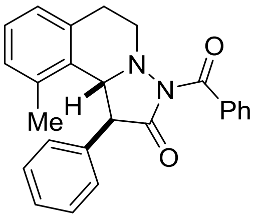

According to ***GP*** with 2-phenylacetic acid (34 mg, 0.25 mmol, 1.0 eq.), *i*-Pr_2EtN (47 µL, 0.28 mmol, 1.1 eq.), benzoyl chloride (29 µL, 0.25 mmol, 1.0 eq.), (*R*)-benzotetramisole (6.3 mg, 25 µmol, 10 mol-%) and benzoyl(8-methyl-3,4-dihydroisoquinolin-2-ium-2-yl)amide (**1c**) (79 mg, 0.30 mmol, 1.2 eq.). FC (P/MTBE = 3/1) afforded the desired pyrazolidinone **3ca** (79 mg, 0.21 mmol, 84%, *exo*/*endo* = 98/2) as a colorless solid.

$[\alpha]_D^{20}$ = +152.4° (c = 1.0 in $CHCl_3$). **MP:** 60 °C. **IR** (neat): 625*w*, 664*m*, 694*s*, 752*s*, 799*w*, 856*w*, 953*w*, 1069*w*, 1126*w*, 1188*m*, 1215*m*, 1269*s*, 1285*s*, 1342*w*, 1451*w*, 1497*w*, 1686*m*, 1747*s*, 2342*w*, 2361*w*, 2932*brw*, 2963*brw*. **^{1}H-NMR** (300 MHz, $CDCl_3$, 300 K): δ (ppm) = 7.79 – 7.72 (*m*, 2H, $C_{arom}H$); 7.57 – 7.50 (*m*, 1H, $C_{arom}H$); 7.46 – 7.30 (*m*, 5H, $C_{arom}H$); 7.24 – 7.20 (*m*, 2H, $C_{arom}H$); 7.13 (*t*, *J* = 7.5 Hz, 1H, $C_{arom}H$); 7.05 (*d*, *J* = 7.4 Hz, 1H, $C_{arom}H$); 6.93 (*d*, *J* = 7.4 Hz, 1H, $C_{arom}H$); 5.14 (*d*, *J* = 9.7 Hz, 1H, NCH); 4.06 (*d*, *J* = 9.7 Hz, 1H, CH); 3.56 (*t*, *J* = 5.9 Hz, 2H, CH_2); 3.08 (*t*, *J* = 5.9 Hz, 2H, CH_2); 1.45 (*s*, 3H, CH_3). **^{13}C-NMR** (75 MHz, $CDCl_3$, 300 K): δ (ppm) = 173.98 (C), 166.55 (C), 136.72 (C), 135.99 (C), 133.88 (C), 133.62 (C), 132.41 (CH), 131.81 (C), 129.31 (3 × CH), 129.29 (2 × CH), 129.23 (2 × CH), 128.26 (CH), 128.09 (2 × CH), 127.44 (CH), 126.58 (CH), 64.35 (CH), 57.03 (CH), 49.13 (CH_2), 27.89 (CH_2), 19.27 (CH_3). **HRMS** (ESI): *m/z* = 405.1573 calcd. for $C_{25}H_{22}N_2O_2Na^+$ $[M+Na]^+$; found: 405.1566.

Enantiomeric excess (97% *ee*) was determined by chiral HPLC analysis (*CHIRALPAK*® *AD-H*, cyclohexane/2-propanol = 90/10, flow rate = 1.0 mL/min, t_r(major) = 21.7 min, t_r(minor) = 8.0 min).

(1*S*,10b*R*)-3-Benzoyl-9-bromo-1-(naphthalen-2-yl)-1,5,6,10b-tetrahydropyrazolo[5,1-*a*]isoquinolin-2(3*H*)-one (3ch)

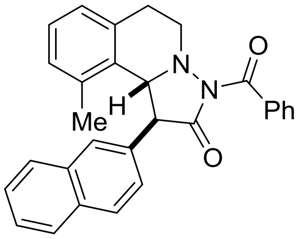

According to ***GP*** with 2-(naphthalen-2-yl)acetic acid (32 mg, 0.20 mmol, 1.0 eq.), *i*-Pr_2EtN (37 µL, 0.22 mmol, 1.1 eq.), benzoyl chloride (23 µL, 0.20 mmol, 1.0 eq.), (*R*)-benzotetramisole (5.0 mg, 20 µmol, 10 mol-%) and benzoyl(8-methyl-3,4-dihydroisoquinolin-2-ium-2-yl)amide (**1c**) (63 mg, 0.24 mmol, 1.2 eq.). FC (P/MTBE = 3/1) afforded the desired pyrazolidinone **3ch** (70 mg, 0.16 mmol, 81%, *exo*/*endo* = 98/2) as a colorless solid.

$[\alpha]_D^{20}$ = +91.7° (c = 1.0 in $CHCl_3$). **MP:** 80 °C. **IR** (neat): 667*w*, 694*m*, 752*s*, 856*w*, 961*m*, 1123*w*, 1192*m*, 1211*m*, 1284*s*, 1373*w*, 1451*w*, 1508*w*, 1601*w*, 1682*s*, 1751*s*, 2342*w*, 2361*w*, 2928*brw*, 3024*brw*. **^{1}H-NMR** (300 MHz, $CDCl_3$, 300 K): δ (ppm) = 7.91 – 7.74 (*m*, 5H, $C_{arom}H$); 7.70 – 7.67 (*m*, 1H, $C_{arom}H$); 7.56 – 7.38 (*m*, 6H, $C_{arom}H$); 7.14 (*t*, *J* = 7.4 Hz, 1H, $C_{arom}H$); 7.07 (*d*, *J* = 7.4 Hz, 1H, $C_{arom}H$); 6.90 (*d*, *J* = 7.3 Hz, 1H, $C_{arom}H$); 5.30 (*d*, *J* = 9.5 Hz, 1H, NCH); 4.24 (*d*, *J* = 9.5 Hz, 1H, CH); 3.69 – 3.50 (*m*, 2H, CH_2); 3.10 (*t*, *J* = 5.9 Hz, 2H, CH_2); 1.42 (*s*, 3H, CH_3). **^{13}C-NMR** (75 MHz, $CDCl_3$, 300 K): δ (ppm) = 173.93 (C), 166.58 (C), 136.71 (C), 133.87 (C), 133.72 (C), 133.57 (C), 133.34 (C), 133.04 (C), 132.47 (CH), 131.96 (C), 129.38 (3 × CH), 129.27 (CH), 128.34 (CH), 128.11 (2 × CH), 128.01 (CH), 127.87 (CH), 127.47 (CH), 126.63 (CH), 126.57 (CH), 126.49 (CH), 126.14 (CH), 64.05 (CH), 57.24 (CH), 49.20 (CH_2), 27.81

(CH$_2$), 19.57 (CH$_3$). **HRMS** (ESI): *m/z* = 455.1730 calcd. for $C_{29}H_{24}N_2O_2Na^+$ [M+Na]$^+$; found: 455.1723.

Enantiomeric excess (89% *ee*) was determined by chiral HPLC analysis (*CHIRALPAK® AD-H*, cyclohexane/2-propanol = 90/10, flow rate = 1.0 mL/min, t_r(major) = 21.9 min, t_r(minor) = 10.1 min).

(1*S*,10b*R*)-3-Benzoyl-9-methoxy-1-phenyl-1,5,6,10b-tetrahydropyrazolo[5,1-*a*]isoquinolin-2(3*H*)-one (3da)

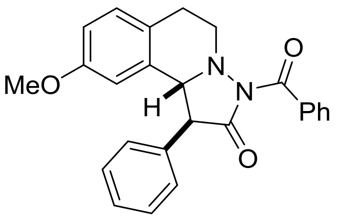

According to ***GP*** with 2-phenylacetic acid (54 mg, 0.40 mmol, 1.0 eq.), *i*-Pr$_2$EtN (72 µL, 0.42 mmol, 1.1 eq.), benzoyl chloride (46 µL, 0.40 mmol, 1.0 eq.), (*R*)-benzotetramisole (10 mg, 40 µmol, 10 mol-%) and benzoyl(7-methoxy-3,4-dihydroisoquinolin-2-ium-2-yl)amide (**1c**) (135 mg, 0.482 mmol, 1.2 eq.). FC (P/MTBE = 2/1) afforded the desired pyrazolidinone **3da** (133 mg, 0.334 mmol, 83%, *exo*/*endo* > 98/2) as a colorless solid.

$[\alpha]_D^{20}$ = +88.4° (c = 1.0 in CHCl$_3$). **MP:** 149 °C. **IR** (neat): 664*w*, 698*m*, 730*m*, 911*w*, 853*w*, 912*w*, 1036*w*, 1116*w*, 1187*m*, 1216*m*, 1273*s*, 1451*w*, 1505*m*, 1614*w*, 1685*m*, 1754*s*, 2252*w*, 2837*w*, 2935*brw*. **^{1}H-NMR** (300 MHz, CDCl$_3$, 300 K): δ (ppm) = 7.76 – 7.69 (*m*, 2H, C_{arom}H), 7.55 – 7.47 (*m*, 1H, C_{arom}H), 7.47 – 7.33 (*m*, 5H, C_{arom}H), 7.28 – 7.21 (*m*, 2H, C_{arom}H), 7.08 (*d*, *J* = 8.5 Hz, 1H, C_{arom}H), 6.75 (*dd*, *J* = 8.5, 2.7 Hz, 1H, C_{arom}H), 5.80 (*d*, *J* = 2.6 Hz, 1H, C_{arom}H), 4.95 (*d*, *J* = 12.0 Hz, 1H, NCH), 4.15 (*d*, *J* = 12.0 Hz, 1H, CH), 3.75 – 3.63 (*m*, 1H, CH$_2$), 3.42 (*s*, 3H, CH$_3$), 3.30 – 3.14 (*m*, 2H, CH$_2$), 2.88 – 2.74 (*m*, 1H, CH$_2$). **^{13}C-NMR** (75 MHz, CDCl$_3$, 300 K): δ (ppm) = 173.6 (C), 166.4 (C), 157.7 (C), 135.2 (C), 134.0 (C), 133.6 (C), 132.2 (CH), 129.8 (2 × CH), 129.7 (CH), 129.3 (2 × CH), 129.0 (2 × CH), 128.4 (CH), 128.1 (2 × CH), 124.8 (C), 115.0 (CH), 111.0 (CH), 65.9 (CH), 55.6 (CH), 55.0 (CH$_3$), 49.1 (CH$_2$), 28.2 (CH$_2$). **HRMS (ESI)** *m/z* = 421.1523 calcd. for $C_{25}H_{22}N_2O_3Na^+$ [M+Na]$^+$; found: 421.1522.

Enantiomeric excess (96% *ee*) was determined by chiral HPLC analysis (*CHIRALPAK® AD-H*, cyclohexane/2-propanol = 90/10, flow rate = 1.0 mL/min, t_r(major) = 15.3 min, t_r(minor) = 11.8 min).

(1*S*,10b*R*)-3-Benzoyl-9-methoxy-1-(4-(trifluoromethyl)phenyl)-1,5,6,10b-tetrahydropyrazolo[5,1-*a*]isoquinolin-2(3*H*)-one (3dg)

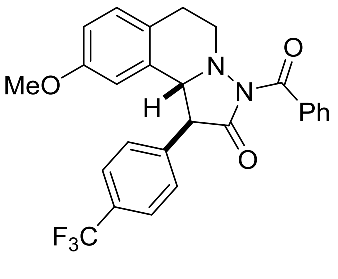

According to ***GP*** with 2-(4-(trifluoromethyl)phenyl)acetic acid (41 mg, 0.20 mmol, 1.0 eq.), *i*-Pr$_2$EtN (37 µL, 0.22 mmol, 1.1 eq.), benzoyl chloride (23 µL, 0.20 mmol, 1.0 eq.), (*R*)-benzotetramisole (5.0 mg, 20 µmol, 10 mol-%) and benzoyl(7-methoxy-3,4-dihydroisoquinolin-2-ium-2-yl)amide (**1c**) (67 mg, 0.24 mmol, 1.2 eq.). FC (P/MTBE = 4/1 → 2/1) afforded the desired pyrazolidinone **3dg** (66 mg, 0.14 mmol, 71%, *exo*/*endo* = 98/2) as a colorless solid.

$[\alpha]_D^{20}$ = +64,2° (c = 1.0 in CHCl$_3$). **MP:** 173 °C. **IR** (neat): 665*w*, 698*m*, 759*m*, 848*w*, 936*w*, 1037*w*, 1070*m*, 1117*s*, 1168*s*, 1274*s*, 1326*s*, 1424*w*, 1466*w*, 1506*m*, 1617*w*, 1688*m*, 1754*m*, 2841*brw*, 2928*brw*, 3012*brw*. **^{1}H-NMR** (300 MHz, CDCl$_3$, 300 K): δ (ppm) = 7.77 – 7.68 (*m*, 4H,

$C_{arom}H$), 7.58 – 7.50 (*m*, 1H, $C_{arom}H$), 7.48 – 7.36 (*m*, 4H, $C_{arom}H$), 7.11 (*d*, *J* = 8.5 Hz, 1H, $C_{arom}H$), 6.78 (*dd*, *J* = 8.5, 2.6 Hz, 1H, $C_{arom}H$), 5.74 (*d*, *J* = 2.6 Hz, 1H, $C_{arom}H$), 4.96 (*d*, *J* = 11.9 Hz, 1H, NCH), 4.26 (*d*, *J* = 11.9 Hz, 1H, CH), 3.76 – 3.63 (*m*, 1H, CH_2), 3.45 (*s*, 3H, CH_3), 3.34 – 3.14 (*m*, 2H, CH_2), 2.91 – 2.75 (*m*, 1H, CH_2). **^{13}C-NMR** (101 MHz, $CDCl_3$, 300 K): δ (ppm) = 172.8 (C), 166.2 (C), 157.7 (C), 139.3 (C), 133.6 (C), 133.1 (C), 132.4 (CH), 130.7 (*q*, *J* = 32.7 Hz, C), 130.3 (2 × CH), 129.9 (CH), 129.0 (2 × CH), 128.1 (2 × CH), 126.2 (*q*, *J* = 3.7 Hz, 2 × CH), 124.7 (C), 124.0 (*q*, *J* = 272.4 Hz, C), 114.8 (CH), 111.1 (CH), 65.8 (CH), 55.4 (CH), 54.9 (CH_3), 49.1 (CH_2), 28.0 (CH_2). **^{19}F-NMR** (282 MHz, CDCl3, 300 K) δ (ppm) = -62.7 (*s*, CF_3). **HRMS (ESI)** *m/z* = 489.1396 calcd. for $C_{26}H_{21}N_2O_3F_3Na^+$ $[M+Na]^+$; found: 489.1385.

Enantiomeric excess (> 99% *ee*) was determined by chiral HPLC analysis (*CHIRALPAK*® *AD-H*, cyclohexane/2-propanol = 90/10, flow rate = 1.0 mL/min, t_r(major) = 17.8 min, t_r(minor) = 14.0 min).

(1*S*,10b*R*)-3-Benzoyl-9-bromo-1-phenyl-1,5,6,10b-tetrahydropyrazolo[5,1-*a*]isoquinolin-2(3*H*)-one (3ea)

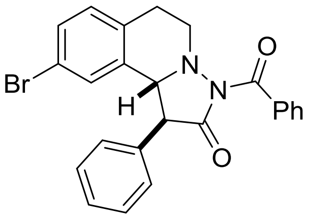

According to ***GP*** with 2-phenylacetic acid (27 mg, 0.20 mmol, 1.0 eq.), *i*-Pr_2EtN (37 µL, 0.22 mmol, 1.1 eq.), benzoyl chloride (23 µL, 0.20 mmol, 1.0 eq.), (*R*)-benzotetramisole (5.0 mg, 20 µmol, 10 mol-%) and benzoyl(7-bromo-3,4-dihydroisoquinolin-2-ium-2-yl)amide (**1d**) (79 mg, 0.24 mmol, 1.2 eq.). FC (P/MTBE = 2/1) afforded the desired pyrazolidinone **3ea** (81 mg, 0.18 mmol, 91%, *exo*/*endo* = 96/4) as a light yellow solid.

$[\boldsymbol{\alpha}]_{\mathbf{D}}^{\mathbf{20}}$ = +68.6° (c = 1.0 in $CHCl_3$). **MP:** 150 °C. **IR** (neat): 641*w*, 663*m*, 698*s*, 811*w*, 878*w*, 898*w*, 954*w*, 1015*w*, 1080*w*, 1121*w*, 1182*m*, 1214*m*, 1286*s*, 1426*w*, 1450*w*, 1485*m*, 1600*w*, 1686*s*, 1755*s*, 2855*brw*, 2930*brw*, 3061*brw*. **^{1}H-NMR** (300 MHz, $CDCl_3$, 300 K): δ (ppm) = 7.74 – 7.68 (*m*, 2H, $C_{arom}H$), 7.56 – 7.38 (*m*, 6H, $C_{arom}H$), 7.32 (*dd*, *J* = 8.2, 2.1 Hz, 1H, $C_{arom}H$), 7.25 – 7.19 (*m*, 2H, $C_{arom}H$), 7.08 (*d*, *J* = 8.2 Hz, 1H, $C_{arom}H$), 6.43 (*d*, *J* = 2.0 Hz, 1H, $C_{arom}H$), 4.92 (*d*, *J* = 11.9 Hz, 1H, NCH), 4.12 (*d*, *J* = 12.0 Hz, 1H, CH), 3.75 (*dt*, *J* = 7.3, 2.1 Hz, 1H, CH_2), 3.32 – 3.17 (*m*, 2H, CH_2), 2.85 (*dt*, *J* = 11.1, 2.3 Hz, 1H, CH_2). **^{13}C-NMR** (75 MHz, $CDCl_3$, 300 K): δ (ppm) = 173.0 (C), 166.2 (C), 134.7 (C), 134.3 (C), 133.6 (C), 132.2 (CH), 131.8 (C), 130.7 (CH), 130.2 (CH), 129.8 (CH), 129.4 (2 × CH), 129.3 (2 × CH), 128.9 (2 × CH), 128.5 (CH), 128.0 (2 × CH), 119.6 (C), 65.0 (CH), 55.3 (CH), 48.3 (CH_2), 28.4 (CH_2). **HRMS (ESI)** *m/z* = 469.0522 calcd. for $C_{24}H_{19}BrN_2O_2Na^+$ $[M+Na]^+$; found: 469.0518.

Enantiomeric excess (75% *ee*) was determined by chiral HPLC analysis (*CHIRALPAK*® *AD-H*, cyclohexane/2-propanol = 90/10, flow rate = 1.0 mL/min, t_r(major) = 18.7 min, t_r(minor) = 11.6 min).

(1*S*,10b*R*)-3-Benzoyl-9-bromo-1-(naphthalen-2-yl)-1,5,6,10b-tetrahydropyrazolo[5,1-*a*]isoquinolin-2(3*H*)-one (3eh)

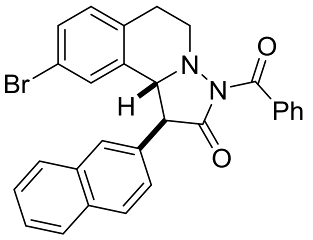

According to ***GP*** with 2-(naphthalen-2-yl)acetic acid (37 mg, 0.20 mmol, 1.0 eq.), *i*-Pr_2EtN (37 µL, 0.22 mmol, 1.1 eq.), benzoyl chloride (23 µL, 0.20 mmol, 1.0 eq.), (*R*)-benzotetramisole (5.0 mg, 20 µmol, 10 mol-%) and benzoyl(7-bromo-3,4-dihydroisoquinolin-2-ium-2-yl)amide (**1d**) (79 mg, 0.24 mmol, 1.2 eq.). FC (P/MTBE = 2/1) afforded the desired pyrazolidinone **3eh** (96 mg, 0.19 mmol, 97%, *exo*/*endo* = 98/2) as a colorless solid.

$[\alpha]_D^{20}$ = +64.7° (c = 1.0 in $CHCl_3$). **MP:** 217 °C. **IR** (neat): 617*w*, 664*m*, 697*m*, 758*m*, 822*w*, 892*m*, 1011*w*, 1130*w*, 1199*m*, 1285*s*, 1485*w*, 1686*s*, 1755*s*, 3049*brw*. **^{1}H-NMR** (300 MHz, $CDCl_3$, 300 K): δ (ppm) = 7.96 (*d*, *J* = 8.5 Hz, 1H, C_{arom}H), 7.87 (*ddd*, *J* = 15.1, 6.8, 3.5 Hz, 2H, C_{arom}H), 7.79 – 7.68 (*m*, 3H, C_{arom}H), 7.54 (*ddd*, *J* = 9.4, 4.8, 1.9 Hz, 3H, C_{arom}H), 7.44 (*dd*, *J* = 8.3, 6.8 Hz, 2H, C_{arom}H), 7.33 (*ddd*, *J* = 12.2, 8.3, 1.9 Hz, 2H, C_{arom}H), 7.08 (*d*, *J* = 8.2 Hz, 1H, C_{arom}H), 6.46 (*d*, *J* = 2.0 Hz, 1H, C_{arom}H), 5.06 (*d*, *J* = 11.8 Hz, 1H, NCH), 4.31 (*d*, *J* = 11.8 Hz, 1H, CH), 3.83 – 3.69 (*m*, 1H, CH_2), 3.36 – 3.16 (*m*, 2H, CH_2), 2.95 – 2.79 (*m*, 1H, CH_2). **^{13}C-NMR** (75 MHz, $CDCl_3$, 300 K): δ (ppm) = 173.2 (C), 166.3 (C), 134.9 (C), 133.7 (C), 133.6 (C), 133.2 (C), 132.3 (CH), 131.9 (C), 131.8 (C), 130.9 (CH), 130.4 (CH), 129.9 (CH), 129.5 (CH), 129.5 (CH), 129.1 (2 × CH), 128.1 (2 × CH), 128.0 (CH), 127.9 (CH), 126.7 (CH), 126.7 (CH), 126.2 (CH), 119.8 (C), 64.9 (CH), 55.6 (CH), 48.6 (CH_2), 28.5 (CH_2). **HRMS** (ESI): *m/z* = 519.0679 calcd. for $C_{28}H_{21}N_2O_2BrNa^+$ $[M+Na]^+$; found: 519.0661.

Enantiomeric excess (87% *ee*) was determined by chiral HPLC analysis (*CHIRALPAK*® *AD-H*, cyclohexane/2-propanol = 92.5/7.5, flow rate = 1.0 mL/min, t_r(major) = 24.3 min, t_r(minor) = 19.8 min).

(2-Hydroxy-1-phenyl-1,5,6,10*b*-tetrahydropyrazolo[5,1-*a*]isoquinolin-3(2*H*)-yl)(phenyl)methanone (4)

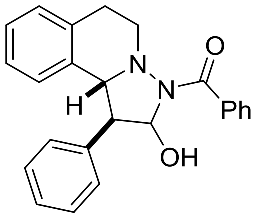

1,4-dimethyl-1*H*-1,2,4-triazol-4-ium iodide[2] (45 mg, 0.20 mmol, 0.1 eq.), 3,3´,5,5´-tetra-*tert*-butyldiphenoquinone[3] (360 mg, 0.881 mmol, 0.4 eq.), DBU (296 µL, 1.98 mmol, 1.0 eq.), and benzoyl(3,4-dihydroisoquinolin-2-ium-2-yl)amide (**1a**) (600 mg, 2.40 mmol, 1.2 eq.) were added subsequently to the reaction vessel. Then CH_2Cl_2 (20 mL) was added, followed by 2-phenylacetaldehyde (232 µL, 1.99 mmol, 1.0 eq.). After stirring at room temperature for 16 h, the solvent was evaporated under reduced pressure. The crude material was purified by FC (P/MTBE = 20/1 → 2/1) to give the desired pyrazolidinol **4** (575 mg, 1.55 mmol, 78%, *dr* > 98/2) as a colorless solid.

MP: 171 °C. **IR** (neat): 628*w*, 701*s*, 762*s*, 954*w*, 1068*m*, 1109*w*, 1241*w*, 1279*w*, 1352*w*, 1421*m*, 1494*w*, 1623*s*, 2935*brw*, 3030*w*, 3395*brw*. **^{1}H-NMR** (300 MHz, $CDCl_3$, 300 K): δ (ppm) = 8.15 – 8.07 (*m*, 2H, C_{arom}H), 7.53 – 7.31 (*m*, 8H, C_{arom}H), 7.19 – 7.08 (*m*, 2H), 6.95 – 6.87 (*m*, 1H, C_{arom}H), 6.23 (*d*, *J* = 7.8 Hz, 1H, C_{arom}H), 6.11 (*d*, *J* = 5.7 Hz, 1H, HOC*H*), 4.64 (*d*, *J* = 11.0 Hz, 1H, NCH), 4.27 (*s*, 1H, OH) 3.72 (*dd*, *J* = 11.0, 5.7 Hz, 1H, CH), 3.58 (*ddd*, *J* = 12.4, 10.5, 3.5 Hz, 1H, CH_2), 3.11 (*ddd*, *J* = 10.5, 5.3, 1.9 Hz, 1H, CH_2), 2.99 (*ddd*, *J* = 17.4, 12.4, 5.3 Hz, 1H, CH_2), 2.72 (*ddd*, *J* = 16.4, 3.5, 2.0 Hz, 1H, CH_2). **^{13}C-NMR** (75 MHz, $CDCl_3$, 300 K):

δ (ppm) = 167.8 (C), 138.1 (C), 133.8 (C), 133.7 (C), 132.9 (C), 131.4 (CH), 129.4 (2 × CH), 129.2 (2 × CH), 128.9 (2 × CH), 128.6 (CH), 128.0 (2 × CH), 127.9 (CH), 127.3 (CH), 126.8 (CH), 125.9 (CH), 91.6 (CH), 68.7 (CH), 59.7 (CH), 50.3 (CH_2), 29.8 (CH_2). **HRMS (ESI)** *m/z* = 393.1573 calcd. for $C_{24}H_{22}N_2O_2Na^+$ $[M+Na]^+$; found: 393.1574.

(*S*)-2-Phenyl-2-((*R*)-1,2,3,4-tetrahydroisoquinolin-1-yl)acetamide (5aa)

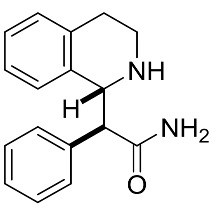

To a stirred suspension of (1*S*,10b*R*)-3-Benzoyl-1-phenyl-1,5,6,10b-tetrahydropyrazolo-[5,1-*a*]isoquinolin-2(3*H*)-one (**3aa**, 74 mg, 0.20 mmol, 1.0 eq.) and LiBr (87 mg, 1.0 mmol, 5.0 eq.) in MeOH (6 mL) at 0 °C was added DBU (60 µL, 0.4 mmol, 2.0 eq.). The reaction mixture was stirred at 0 °C for 1 h and then quenched with NH_4Cl (12 mL, aq. sat., x mL). After extraction with CH_2Cl_2 (3x), the combined organic phases were dried over Na_2SO_4 and concentrated under reduced pressure. The so obtained crude mixture was transferred to a Schlenk tube and dissolved in EtOH (4 mL). Raney nickel® (50% slurry in H_2O, approx. 704 mg, 6.0 mmol, 30 eq.) was added and the reaction mixture was stirred under an atmosphere of H_2 at 50 °C. After 16 h, it was filtered through a pad of Celite with EtOH. The solvent was removed and the residue was purified by FC (EtOAc/MeOH/Et_3N = 100/5/0,5) to give the desired amide **5aa** (41 mg, 0.15 mmol, 77%) as a light orange solid.

$[\alpha]_D^{20}$ = -29.1° (c = 1.0 in $CDCl_3$). **MP:** 51 °C. **IR** (neat): 701*m*, 722*m*, 746*m*, 951*w*, 1035*w*, 1124*w*, 1299*w*, 1322*w*, 1396*w*, 1454*m*, 1496*w*, 1667*s*, 2810*w*, 2925*w*, 3028*w*, 3063*w*, 3179*brw*, 3327*brw*. **^{1}H-NMR** (300 MHz, $CDCl_3$, 300 K): δ (ppm) = 8.59 (*s*, 2H, NH_2), 7.48 – 7.27 (*m*, 6H, $C_{arom}H$), 7.21 – 7.08 (*m*, 3H, $C_{arom}H$), 5.50 (*s*, 1H, NH), 4.44 (*d*, *J* = 4.4 Hz, 1H, CH), 4.41 (*d*, *J* = 4.4 Hz, 1H, CH), 3.23 (*ddd*, *J* = 11.0, 5.5, 2.3 Hz, 1H, CH_2), 3.09 – 2.95 (*m*, 1H, CH_2), 2.85 (*td*, *J* = 11.0, 3.4 Hz, 1H, CH_2), 2.73 (*dt*, *J* = 15.5, 2.9 Hz, 1H, CH_2). **^{13}C-NMR** (75 MHz, $CDCl_3$, 300 K): δ (ppm) = 173.65 (C), 137.28 (C), 135.77 (C), 135.27 (C), 129.44 (CH), 129.17 (2 × CH), 128.53 (2 × CH), 127.67 (CH), 126.81 (CH), 126.57 (CH), 125.44 (CH), 59.37 (CH), 56.93 (CH), 43.09 (CH_2), 30.04 (CH_2). **HRMS** (ESI): *m/z* = 267.1492 calcd. for $C_{17}H_{18}N_2OH^+$ $[M+H]^+$; found: 267.1491.

(*S*)-2-((*R*)-7-Methoxy-1,2,3,4-tetrahydroisoquinolin-1-yl)-2-phenylacetamide (5da)

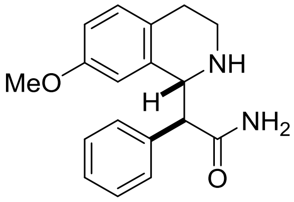

According to the preparation of **5aa** with (1*S*,10b*R*)-3-benzoyl-9-methoxy-1-phenyl-1,5,6,10b-tetrahydropyrazolo[5,1-*a*]isoquinolin-2(3*H*)-one (**3da,** 63 mg, 0.16 mmol, 1.0 eq.), LiBr (69 mg, 0.79 mmol, 5.0 eq.), DBU (47 µL, 0.32 mmol, 2.0 eq.) and subsequent reduction by Raney nickel® (50% slurry in H_2O, approx. 552 mg, 4.7 mmol, 30 eq.). FC (EtOAc/MeOH/Et_3N = 100/3/1) afforded the desired amide **5da** (31 mg, 0.10 mmol, 65%) as a light orange solid.

$[\alpha]_D^{20}$ = -36.6° (c = 0.9 in $CDCl_3$). **MP:** 138 °C. **IR** (neat): 702*m*, 725*m*, 757*w*, 809*w*, 856*w*, 951*w*, 1038*m*, 1132*w*, 1160*w*, 1247*s*, 1269*m*, 1312*m*, 1395*w*, 1505*s*, 1611*m*, 1668*s*, 2834*w*, 2916*brw*, 3197*brw*, 3317*brw*. **^{1}H-NMR** (300 MHz, $CDCl_3$, 300 K): δ (ppm) = 8.45 (*s*, 2H, NH_2), 7.48 – 7.27 (*m*, 5H, $C_{arom}H$), 7.06 – 6.97 (*m*, 1H, $C_{arom}H$), 6.78 – 6.70 (*m*, 2H, $C_{arom}H$), 5.52 (*s*, 1H, NH), 4.40 (*d*, *J* = 4.4 Hz, 1H, CH), 4.33 (*d*, *J* = 4.4 Hz, 1H, CH), 3.71 (*s*, 3H, CH_3), 3.21 (*ddd*, *J* = 10.9, 4.7, 2.7 Hz, 1H, CH_2), 2.99 – 2.87 (*m*, 1H, CH_2), 2.82 (*td*, *J* = 10.4, 2.9 Hz, 1H, CH_2), 2.72 – 2.62 (*m*, 1H, CH_2). **^{13}C-NMR** (75 MHz, $CDCl_3$, 300 K): δ (ppm) = 173.68 (C), 158.04 (C), 137.41 (C),

136.83 (C), 130.30 (CH), 129.12 (2 × CH), 128.59 (2 × CH), 127.63 (CH), 127.34 (C), 113.34 (CH), 110.55 (CH), 59.42 (CH), 57.08 (CH), 55.39 (CH3), 43.01 (CH_2), 29.27 (CH_2). **HRMS** (ESI): *m/z* = 297.1598 calcd. for $C_{18}H_{20}N_2O_2H^+$ $[M+H]^+$; found: 297.1596.

3. X-ray crystallographic data of pyrazolidinone 3aa

X-ray diffraction: Data sets were collected with a Nonius KappaCCD diffractometer. Programs used: data collection, COLLECT (Nonius B.V., 1998); data reduction Denzo-SMN[4]; absorption correction, Denzo[5]; structure solution SHELXS-97[6]; structure refinement SHELXL-97[7] and graphics, XP (*BrukerAXS, 2000*). *R*-values are given for observed reflections, and wR^2 values are given for all reflections.

X-ray crystal structure analysis of 3aa: formula $C_{24}H_{20}N_2O_2$, $M = 368.42$, colourless crystal, 0.12 x 0.05 x 0.03 mm, $a = 7.5800(1)$, $b = 13.2149(1)$, $c = 18.5282(1)$ Å, $V = 1855.95(3)$ Å^3, $\rho_{calc} = 1.319$ gcm^{-3}, $\mu = 0.673$ mm^{-1}, empirical absorption correction ($0.923 \leq T \leq 0.980$), $Z = 4$, orthorhombic, space group $P2_12_12_1$ (No. 19), $\lambda = 1.54178$ Å, $T = 223(2)$ K, ω and φ scans, 9433 reflections collected ($\pm h$, $\pm k$, $\pm l$), $[(\sin\theta)/\lambda] = 0.60$ Å^{-1}, 2976 independent ($R_{int} = 0.042$) and 2727 observed reflections [$I > 2\sigma(I)$], 253 refined parameters, $R = 0.035$, $wR^2 = 0.082$, max. (min.) residual electron density 0.11 (-0.12) e.Å^{-3}, hydrogen atoms calculated and refined as riding atoms. Flack parameter: 0.0(3).

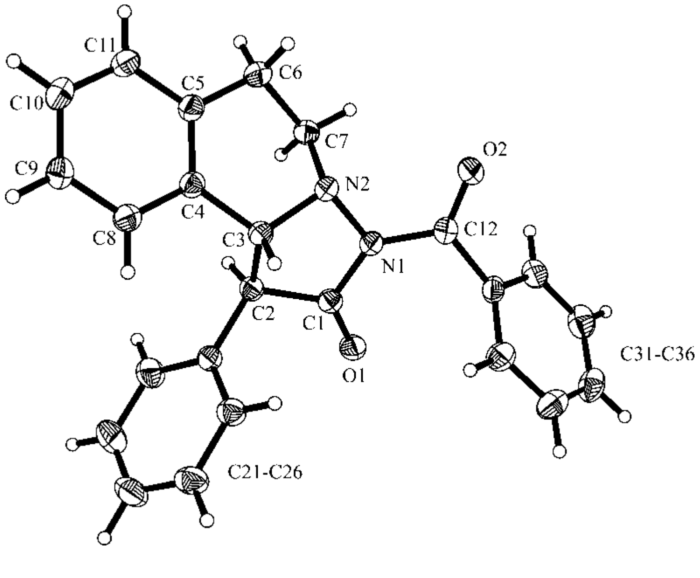

Figure S1: Crystal structure of compound **3aa**.

Thermals ellipsoids are shown with 30% probability.

4. DFT calculations

DFT geometry optimizations (in vacuum) were performed with TURBOMOLE,[8] using the TPSS meta-GGA functional,[9] the triple zeta basis set def2-TZVP,[10] and the dispersion correction of Grimme et al.[11] with BJ damping[12] (TPSS-D3/def2-TZVP). The calculation of vibrational normal modes was done for all structures and the contribution of translations, rotations and normal vibrations to the free energy at 298 K (G_{298}) added to the electronic energy obtained in single point calculations with the COSMO solvation model[13] using $\varepsilon = 9.08$ (CH_2Cl_2).

Table S1: Absolute Energies (TPSS-D3/def2-TZVP + COSMO(CH_2Cl_2) for all reported structures.

	E(vac) [Eh]	E(COSMO) [Eh]	G298(vac) [kcal/mol]	H298(vac) [kcal/mol]	ΔE(vac)[a] [kcal/mol]	ΔE(COSMO)[a] [kcal/mol]	ΔG(298)[a] (COSMO) [kcal/mol]	ΔH(298)[a] (COSMO) [kcal/mol]
**F**	-1470.888969	-1470.914053	187.29	232.45				
**1a**	-803.317751	-803.333750	137.98	173.95				
exo:								
**CP-1**	-2274.251946	-2274.275634	343.22	408.35	-28.4	-17.5	0.5	-15.5
**TS-1**	-2274.232961	-2274.258246	343.84	407.58	-16.5	-6.6	12.0	-5.4
**IN-1**	-2274.243010	-2274.267381	345.48	408.70	-22.8	-12.3	7.9	-10.0
**TS-2**	-2274.241574	-2274.265079	345.62	407.97	-21.9	-10.8	9.5	-9.3
**CP-2**	-2274.264521	-2274.284187	343.77	407.87	-36.3	-22.8	-4.3	-21.4
endo:								
**CP-1**	-2274.245053	-2274.270764	342.30	408.17	-24.1	-14.4	2.6	-12.6
**TS-1**	-2274.218685	-2274.250040	343.52	407.56	-7.5	-1.4	16.9	-0.2
**IN-1**	-2274.224067	-2274.256534	345.03	408.62	-10.9	-5.5	14.3	-3.3
**TS-2**	-2274.223632	-2274.255433	344.90	407.95	-10.6	-4.8	14.9	-3.2
**IN-1**	-2274.233623	-2274.261651	345.05	408.66	-16.9	-8.7	11.1	-6.4
**CP-2**	-2274.245053	-2274.270764	343.33	408.03	-24.1	-14.4	3.7	-12.8
**E**	-1086.977583	-1086.988965	119.42	154.30				
trans-**3aa**[b]	-1187.259569	-1187.277160	207.28	252.50	-19.1	-11.5	-10.1	-11.1
cis-**3aa**[b]	-1187.258118	-1187.276211	207.41	252.74	-18.2	-10.9	-9.3	-10.3

[a] energies ΔE, ΔG and ΔH are relative to isolated **F** and **1a**.
[b] relative energies for **3aa** + **E**

Optimized geometries (TPSS-D3/def2-TZVP)

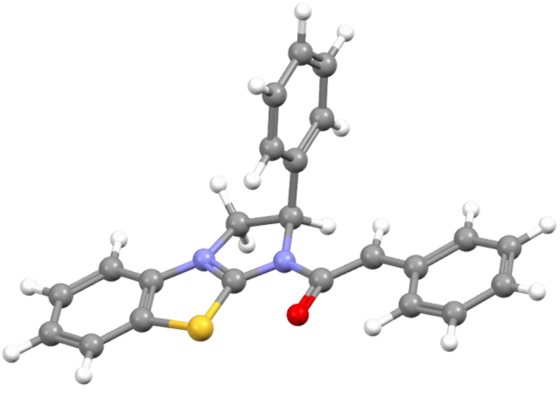

F

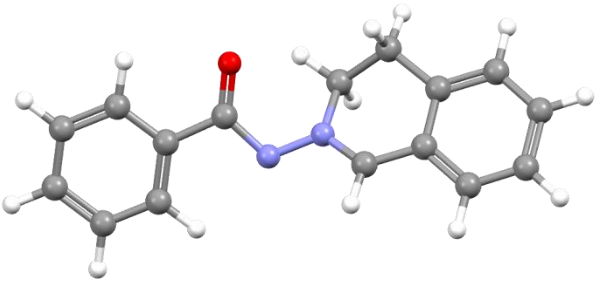

1a

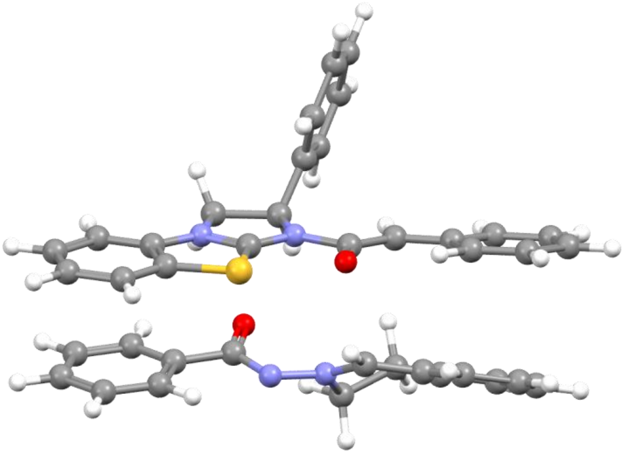

***exo*-CP-1**

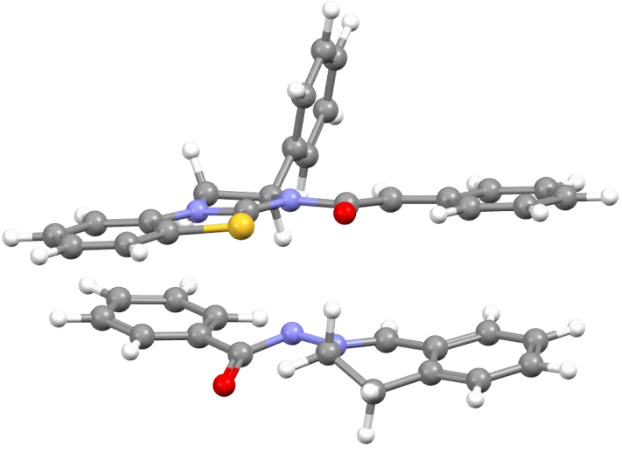

***endo*-CP-1**

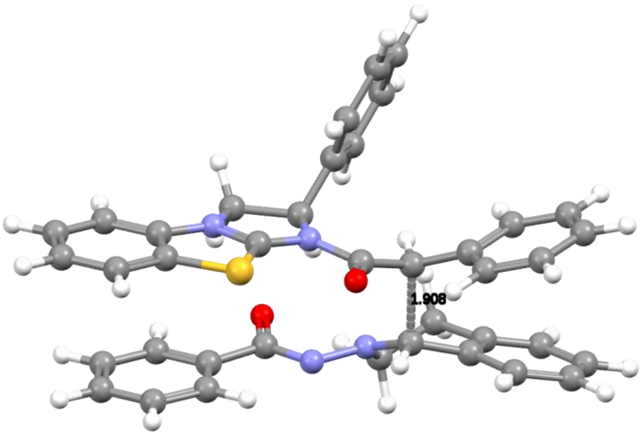

***exo*-TS-1**

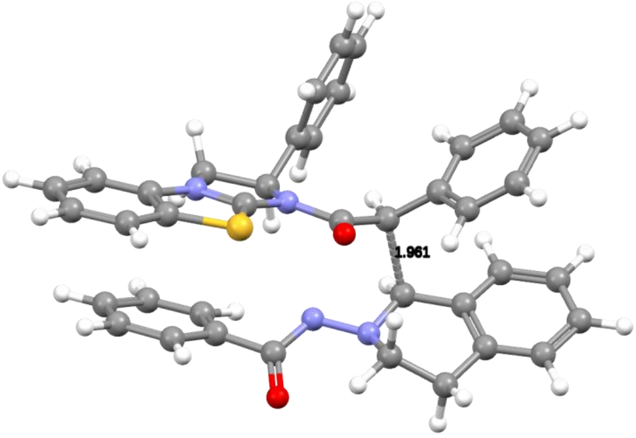

***endo*-TS-1**

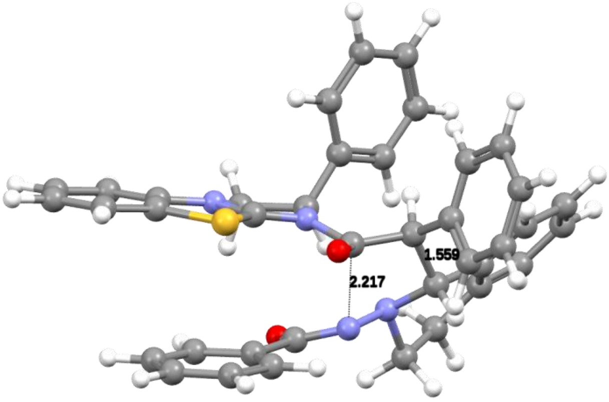

***exo*-IN-1**

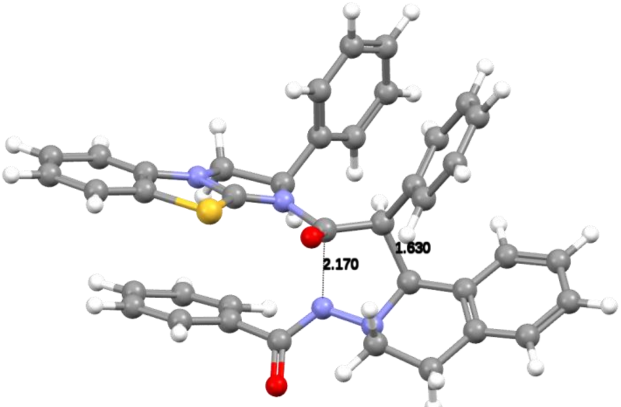

***endo*-IN-1**

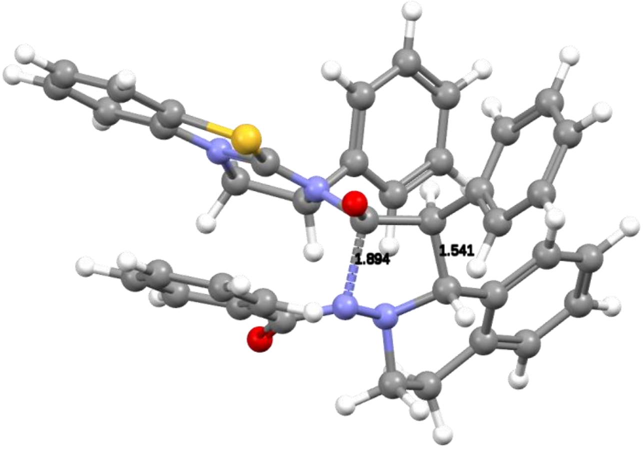

***exo*-TS-2**

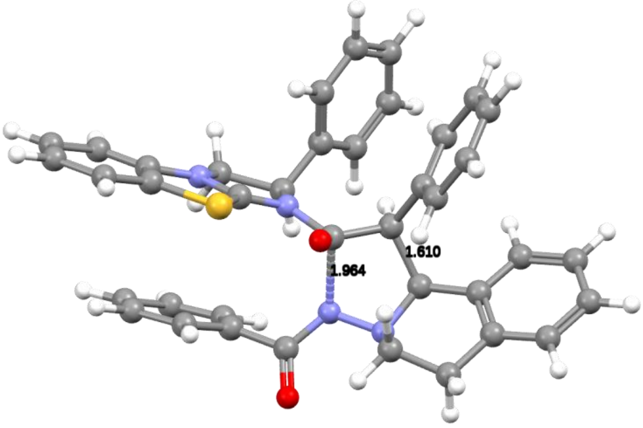

***endo*-TS-2**

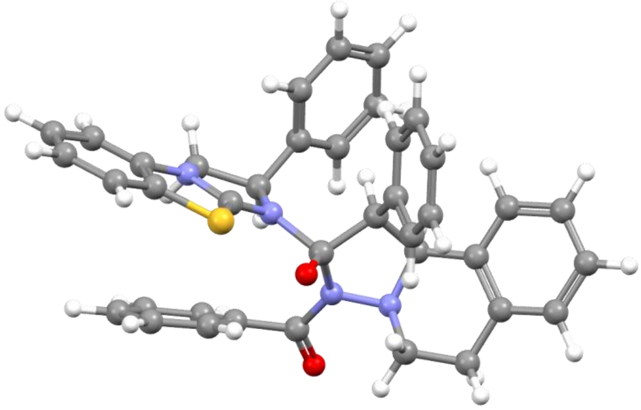

***endo*-IN-2**

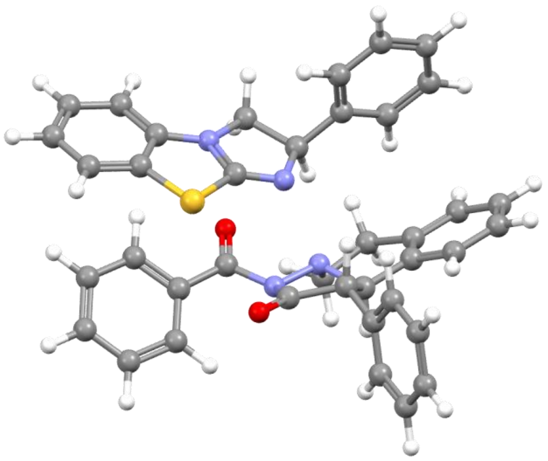

***exo*-CP-2**

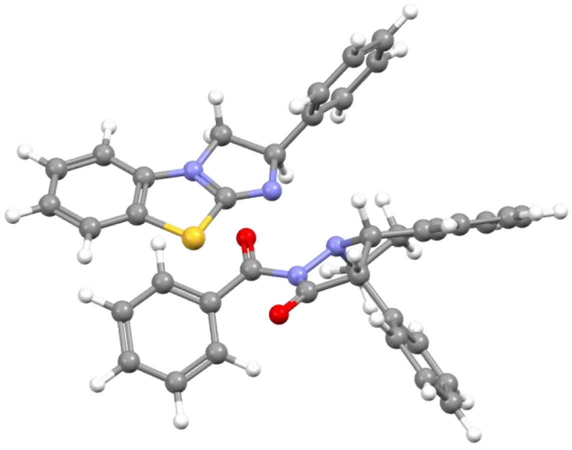

***endo*-CP-2**

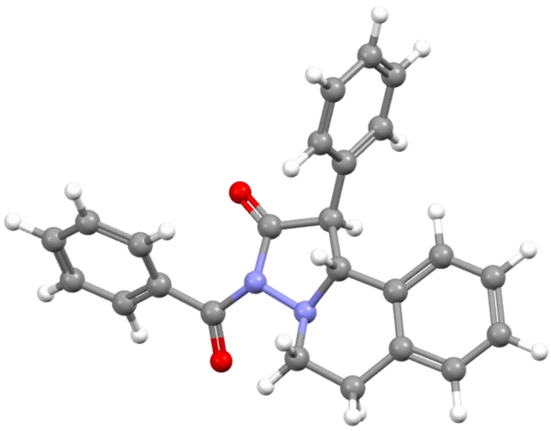

trans-3aa

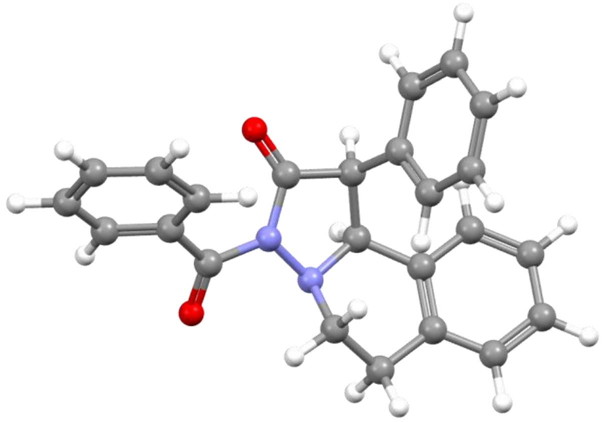

cis-3aa

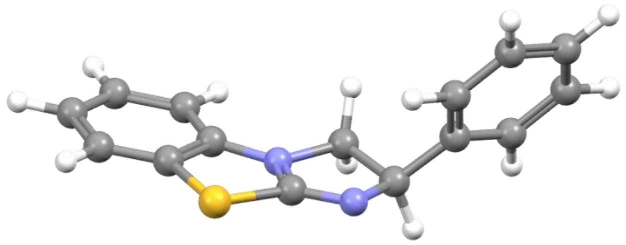

E

5. HPLC chromatograms of cycloaddition products

(1*S*,10b*R*)-3-Benzoyl-1-phenyl-1,5,6,10b-tetrahydropyrazolo[5,1-*a*]isoquinolin-2(3*H*)-one (3aa)

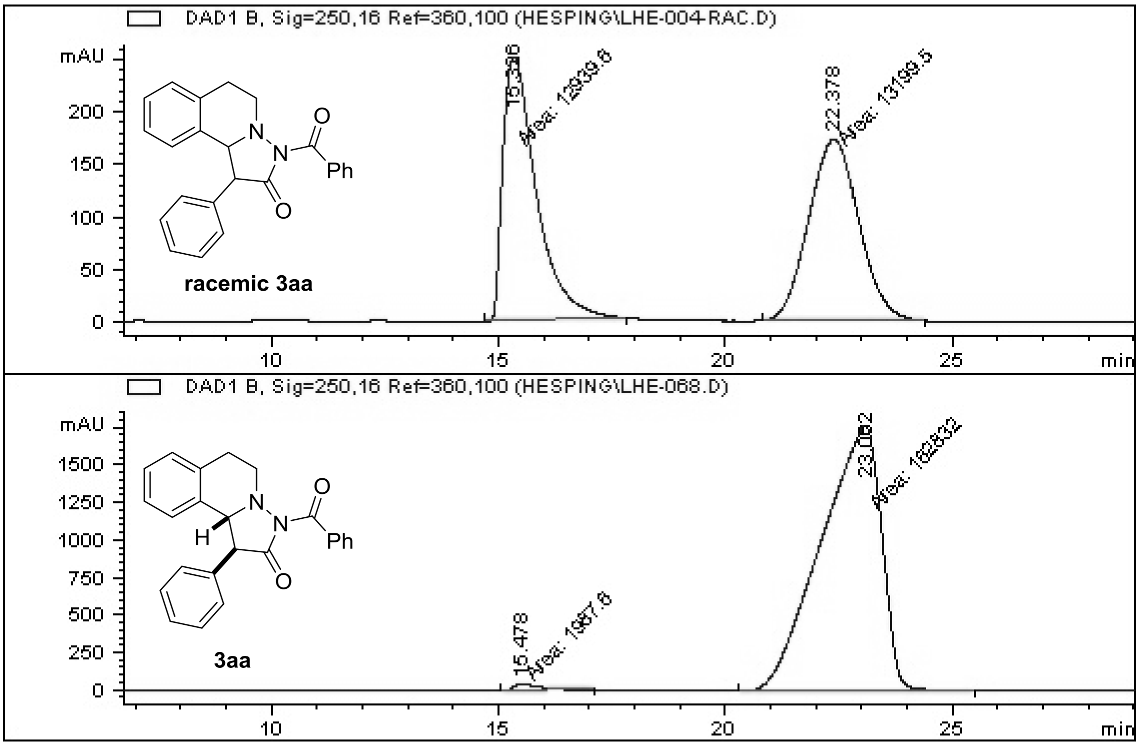

(1*S*,10b*R*)-3-Benzoyl-1-(*o*-tolyl)-1,5,6,10b-tetrahydropyrazolo[5,1-*a*]isoquinolin-2(3*H*)-one (3ab)

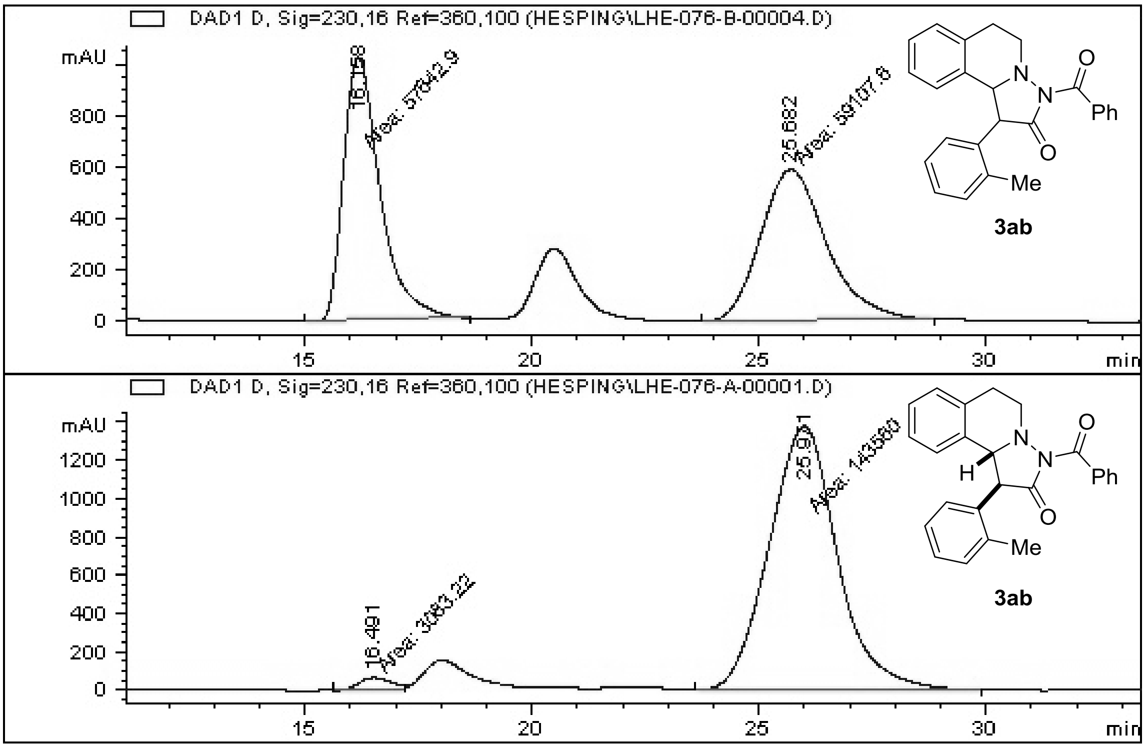

(1*S*,10b*R*)-3-Benzoyl-1-(4-methoxyphenyl)-1,5,6,10b-tetrahydropyrazolo[5,1-*a*]isoquinolin-2(3*H*)-one (3ac)

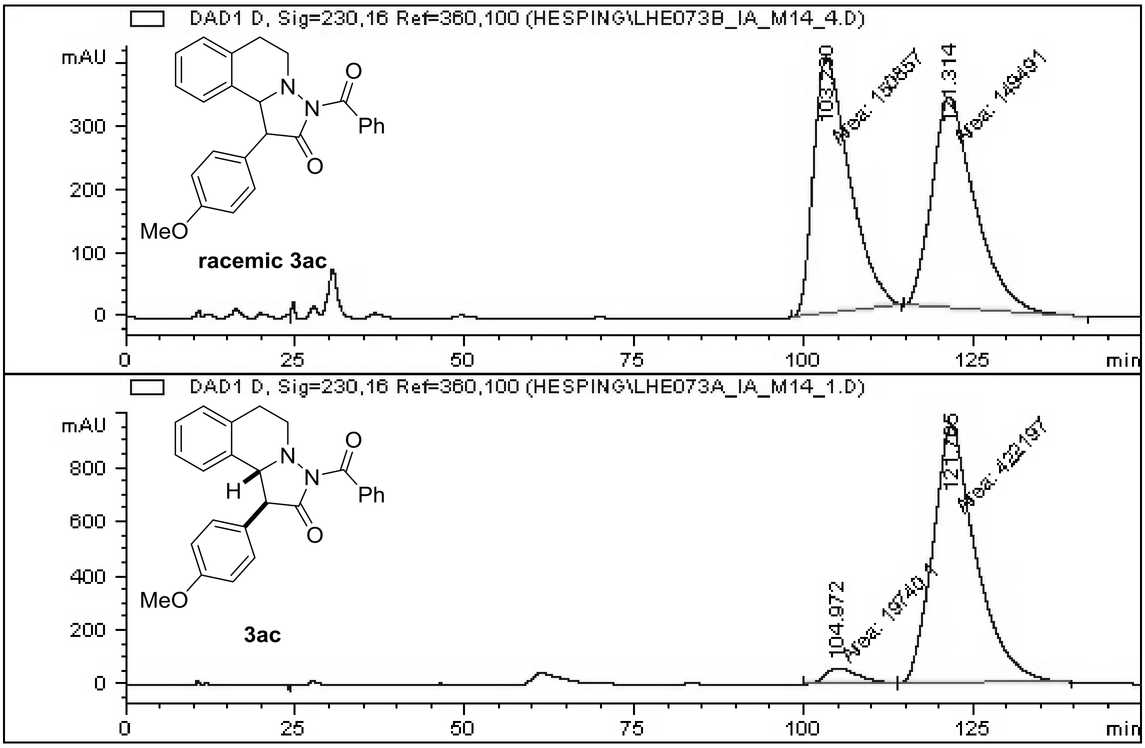

(1*S*,10b*R*)-3-Benzoyl-1-(4-chlorophenyl)-1,5,6,10b-tetrahydropyrazolo[5,1-*a*]isoquinolin-2(3*H*)-one (3ad)

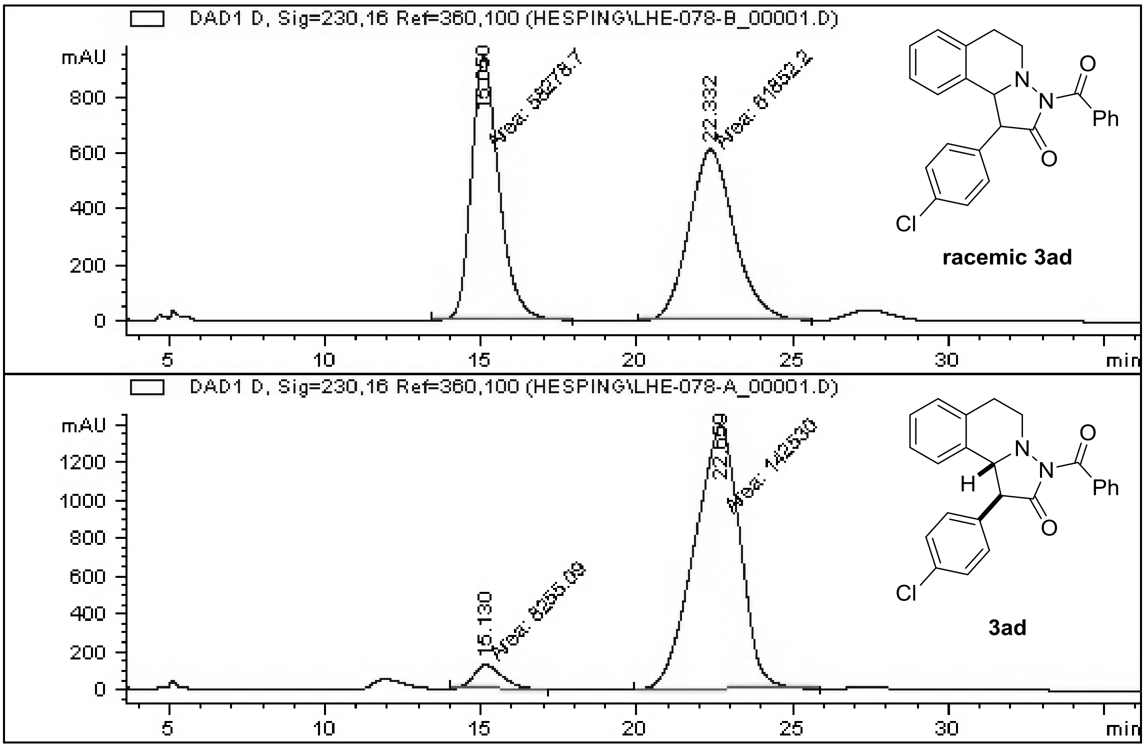

(1*S*,10b*R*)-3-Benzoyl-1-(3-chlorophenyl)-1,5,6,10b-tetrahydropyrazolo[5,1-*a*]isoquinolin-2(3*H*)-one (3ae)

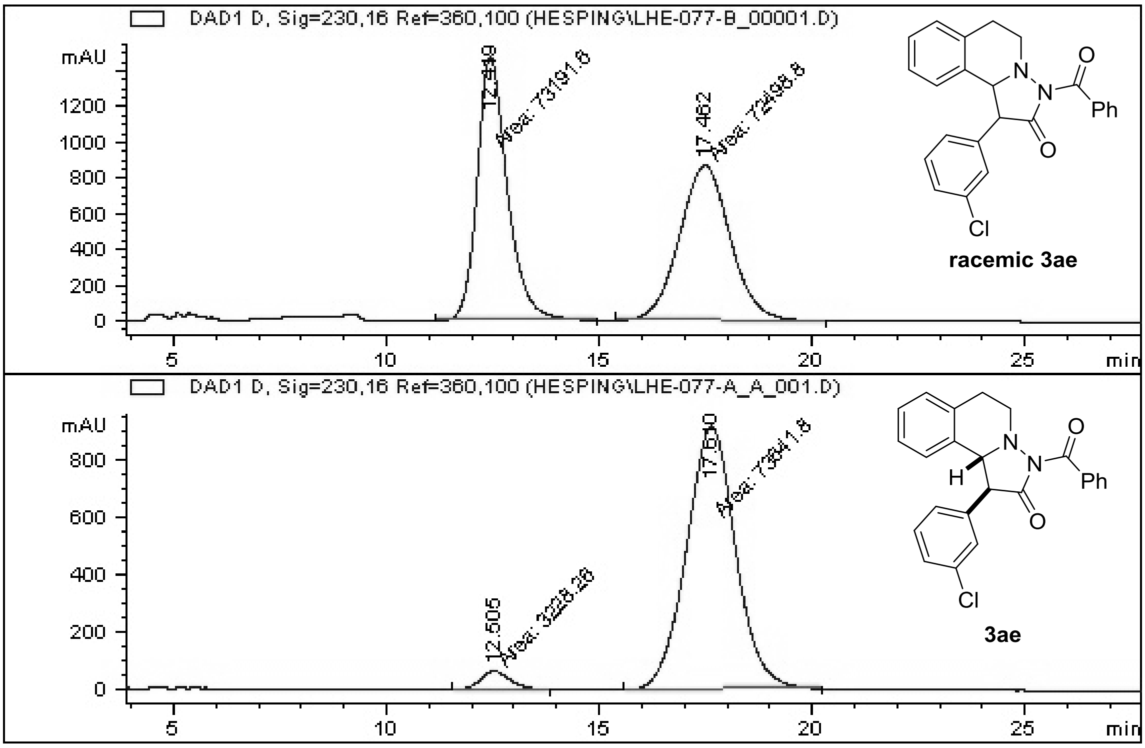

(1*S*,10b*R*)-3-Benzoyl-1-(2-fluorophenyl)-1,5,6,10b-tetrahydropyrazolo[5,1-*a*]isoquinolin-2(3*H*)-one (3af)

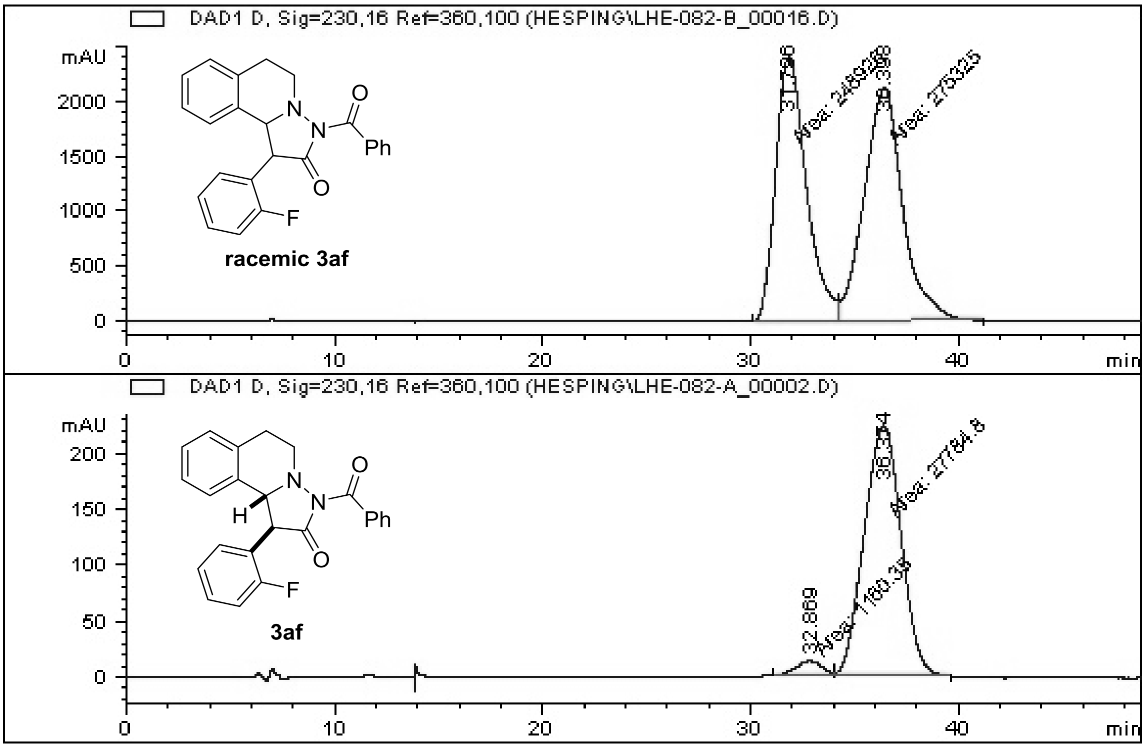

(1*S*,10b*R*)-3-Benzoyl-1-(4-(trifluoromethyl)phenyl)-1,5,6,10b-tetrahydropyrazolo[5,1-*a*]isoquinolin-2(3*H*)-one (3ag)

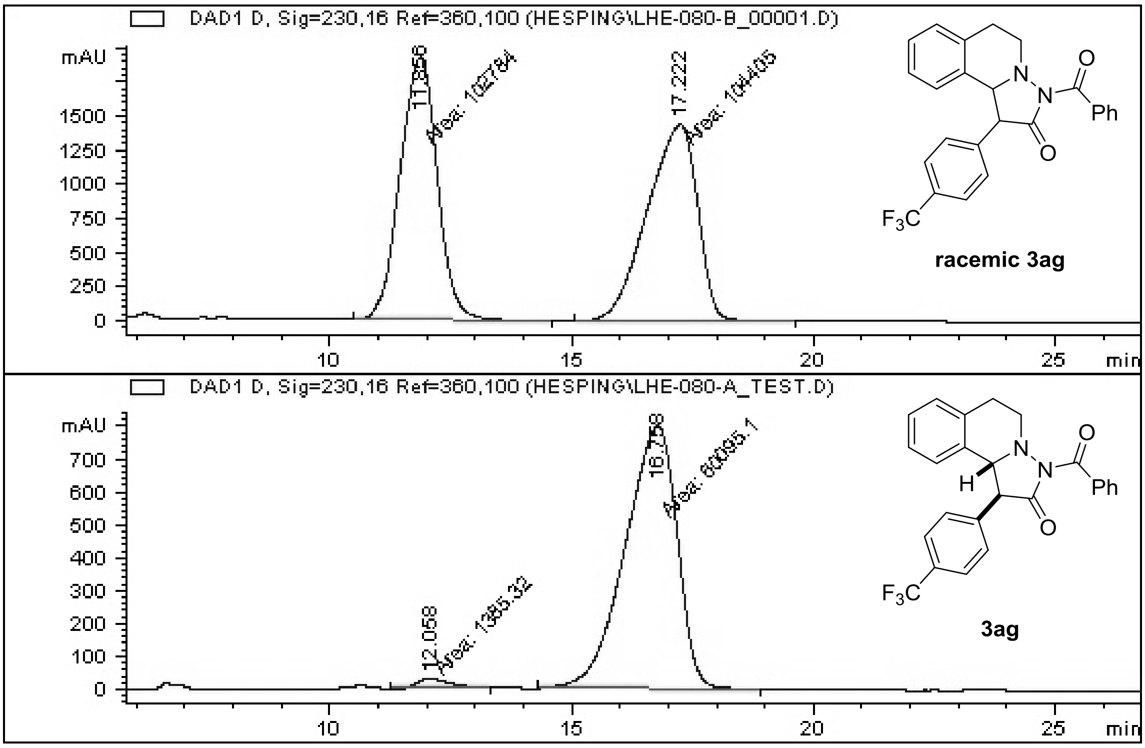

(1*S*,10b*R*)-3-Benzoyl-1-(naphthalen-2-yl)-1,5,6,10b-tetrahydropyrazolo[5,1-*a*]isoquinolin-2(3*H*)-one (3ah)

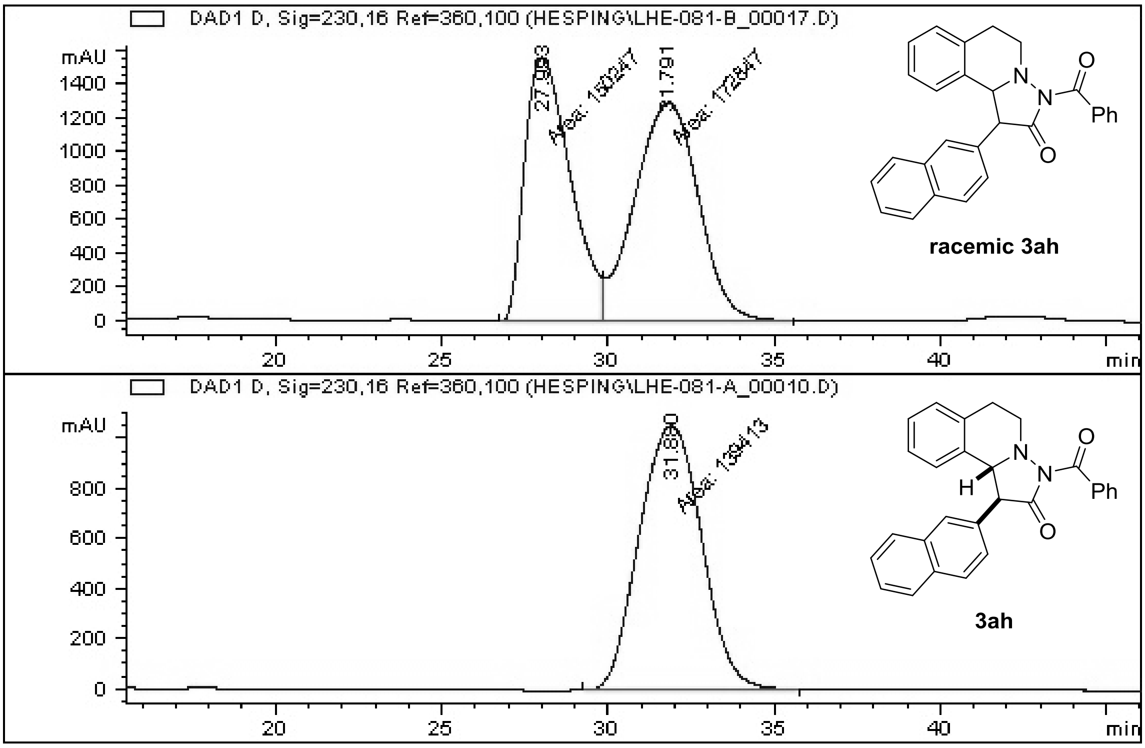

(1*S*,10b*R*)-3-Benzoyl-1-(thiophen-2-yl)-1,5,6,10b-tetrahydropyrazolo[5,1-*a*]isoquinolin-2(3*H*)-one (3ai)

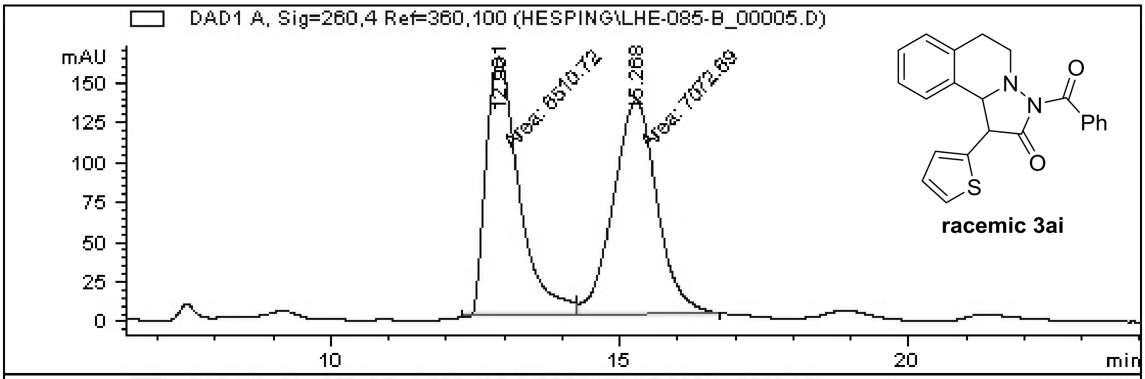

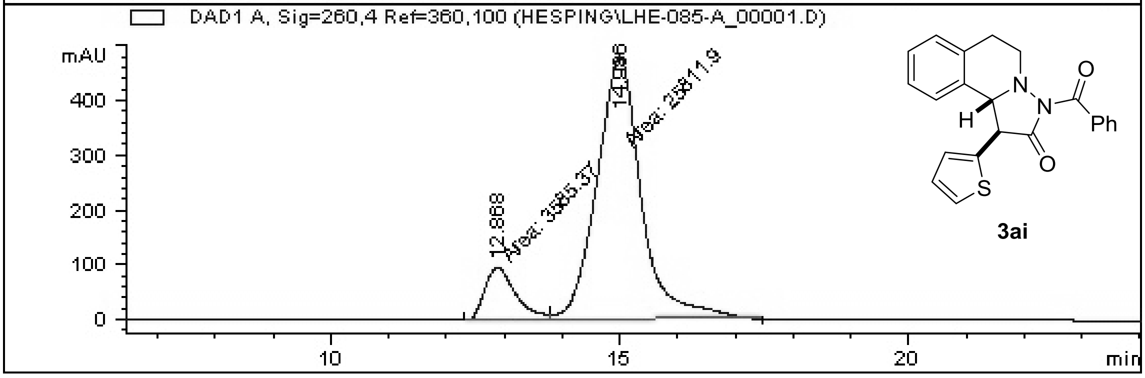

(1*S*,10b*R*)-3-Benzoyl-7-methyl-1-phenyl-1,5,6,10b-tetrahydropyrazolo[5,1-*a*]isoquinolin-2(3*H*)-one (3ba)

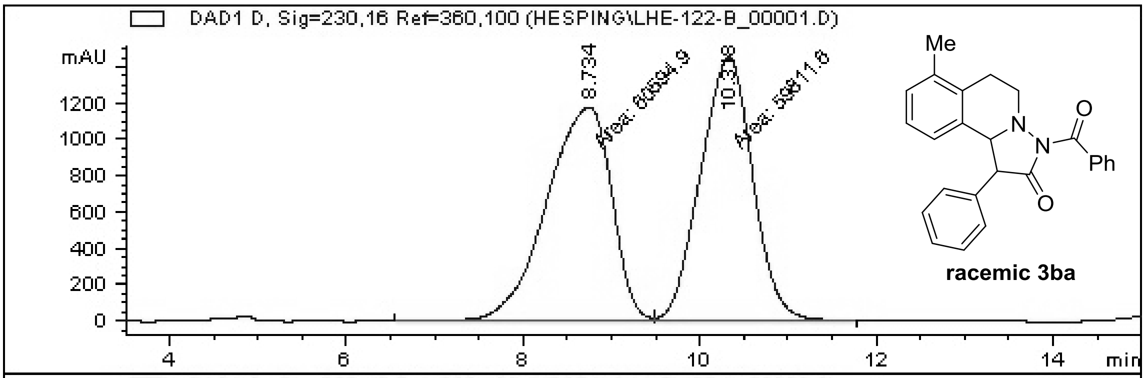

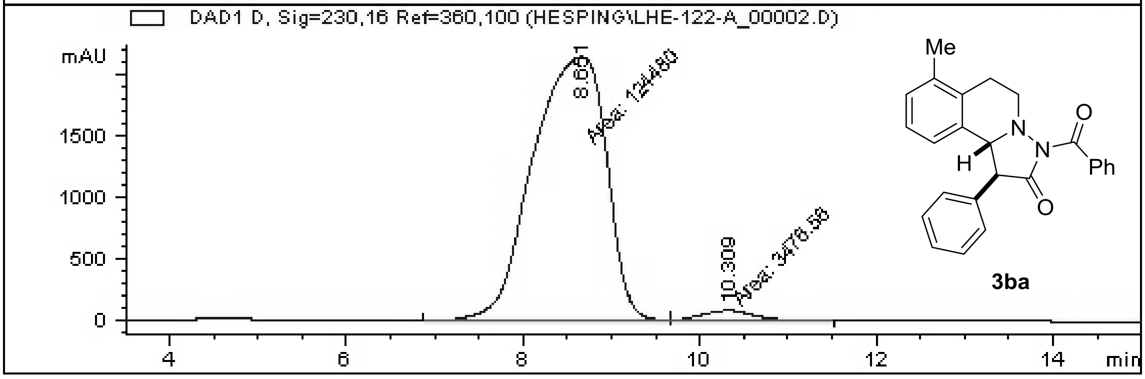

(1*S*,10b*R*)-3-Benzoyl-7-methyl-1-(naphthalen-2-yl)-1,5,6,10b-tetrahydropyrazolo[5,1-*a*]isoquinolin-2(3*H*)-one (3bh)

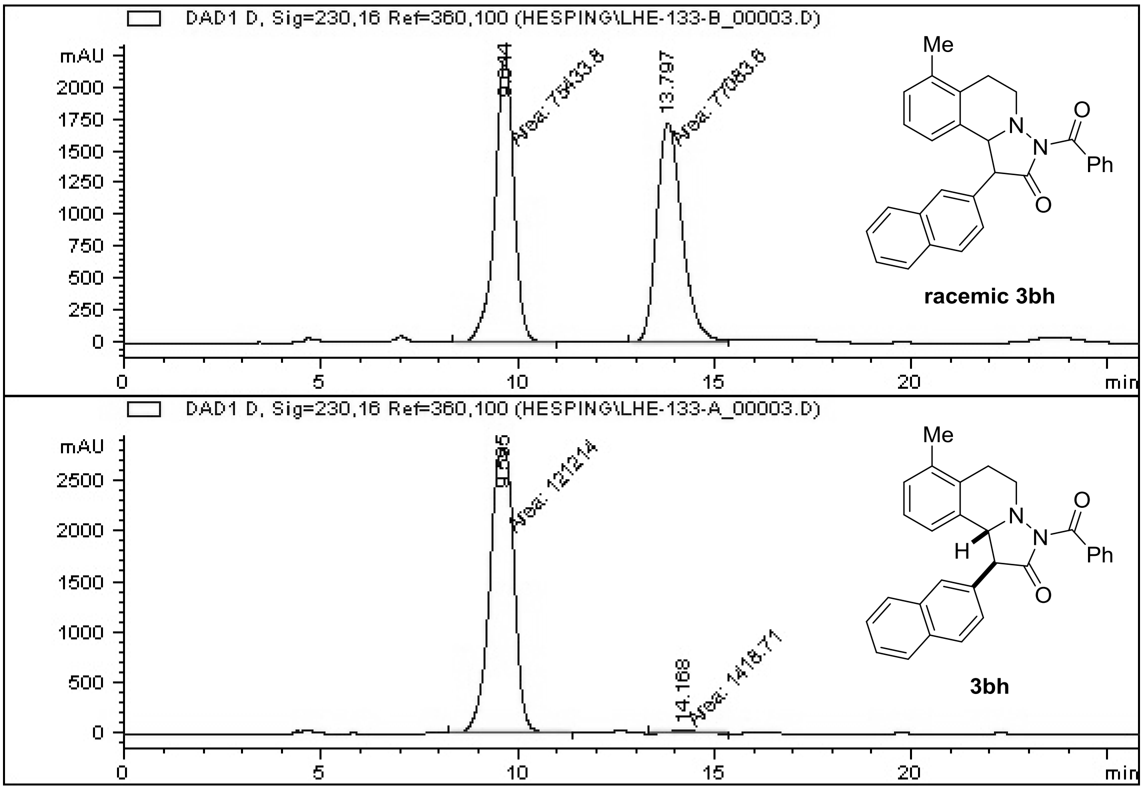

(1*S*,10b*R*)-3-Benzoyl-10-methyl-1-phenyl-1,5,6,10b-tetrahydropyrazolo[5,1-*a*]isoquinolin-2(3*H*)-one (3ca)

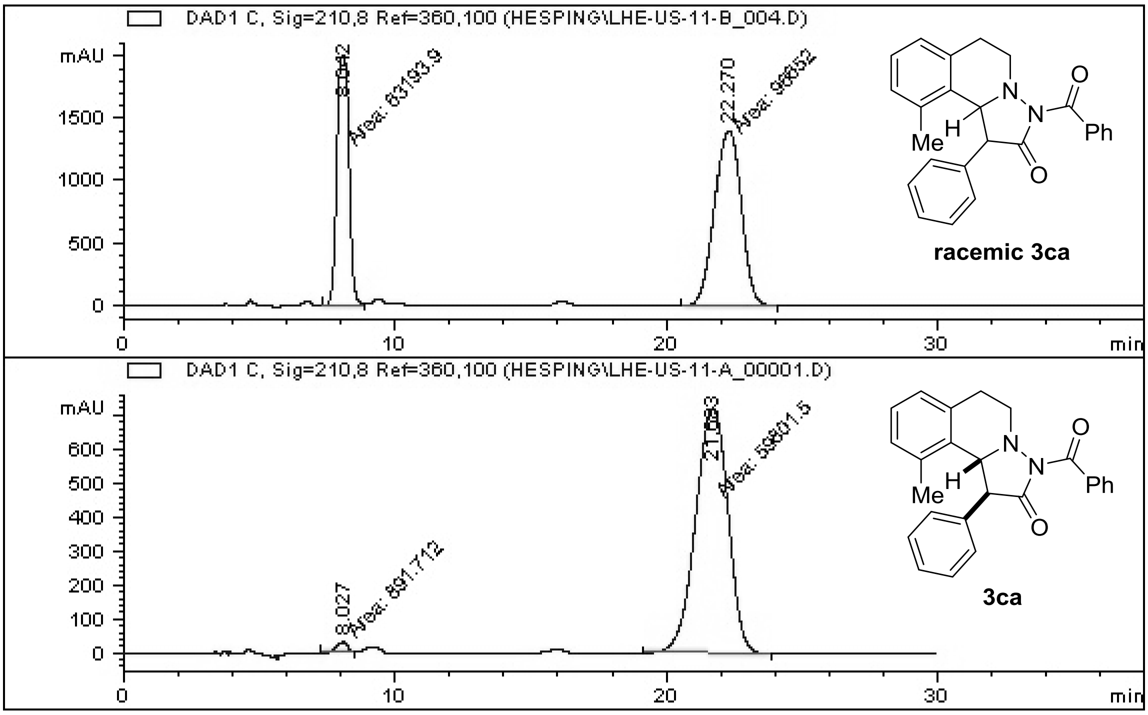

(1*S*,10b*R*)-3-Benzoyl-9-bromo-1-(naphthalen-2-yl)-1,5,6,10b-tetrahydropyrazolo[5,1-*a*]isoquinolin-2(3*H*)-one (3ch)

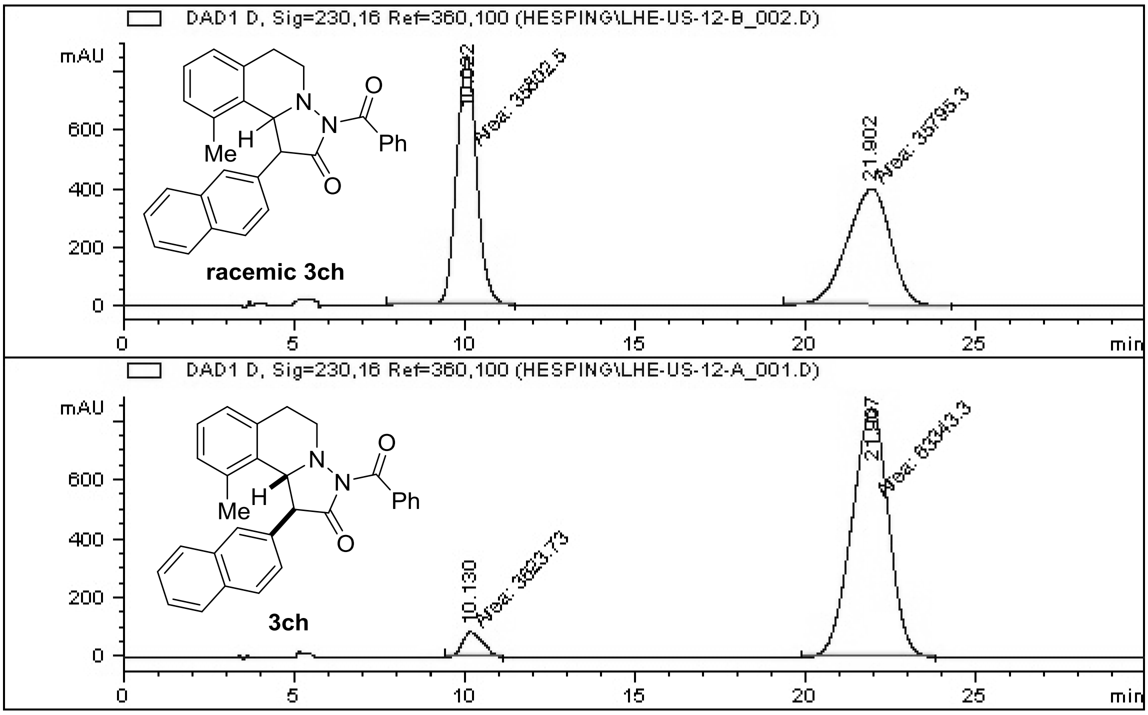

(1*S*,10b*R*)-3-Benzoyl-9-methoxy-1-phenyl-1,5,6,10b-tetrahydropyrazolo[5,1-*a*]isoquinolin-2(3*H*)-one (3da)

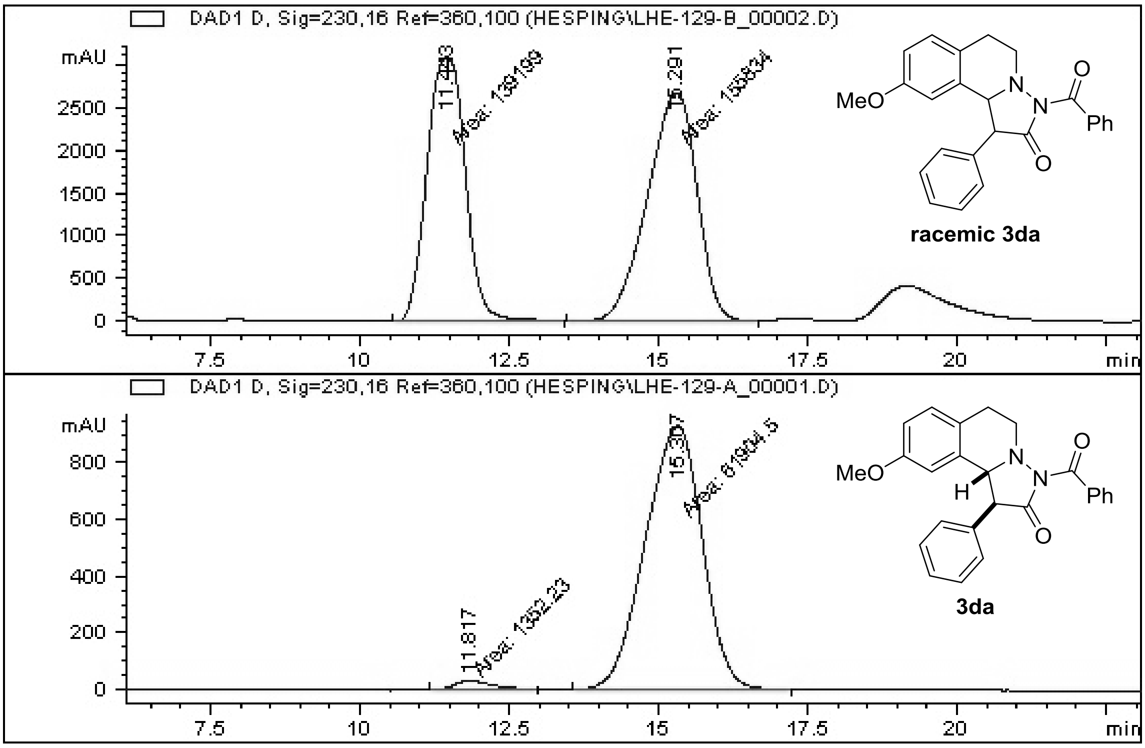

(1*S*,10b*R*)-3-Benzoyl-9-methoxy-1-(4-(trifluoromethyl)phenyl)-1,5,6,10b-tetrahydropyrazolo[5,1-*a*]isoquinolin-2(3*H*)-one (3dg)

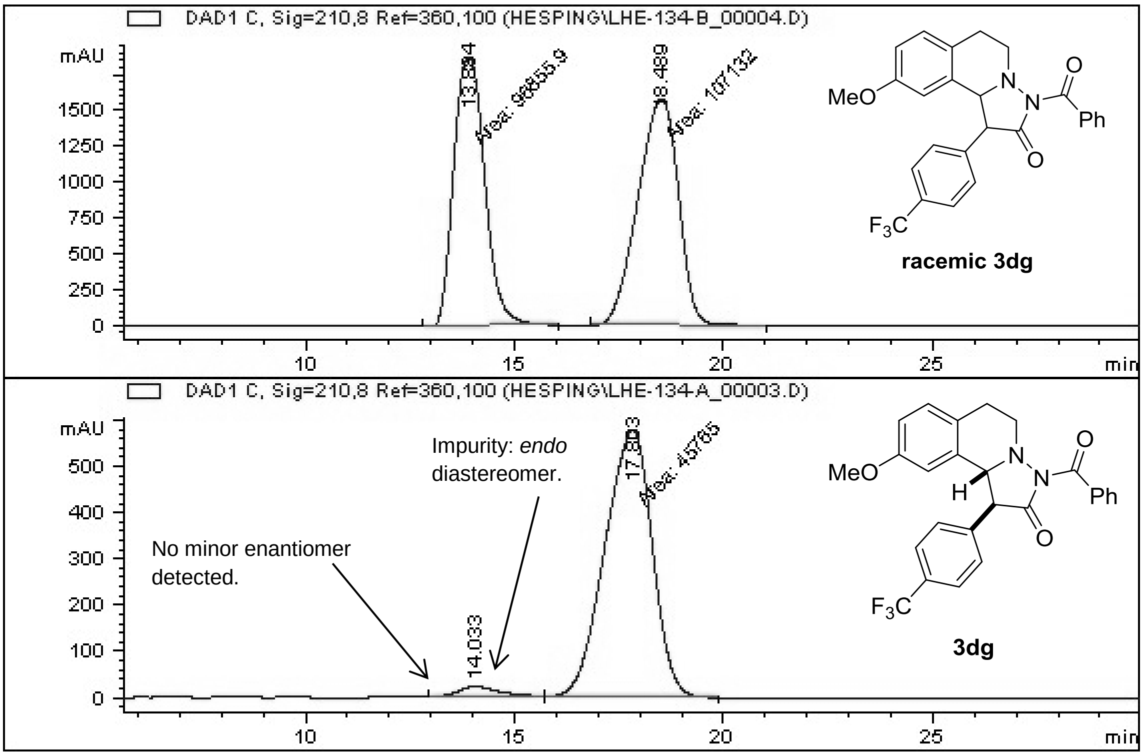

(1*S*,10b*R*)-3-Benzoyl-9-bromo-1-phenyl-1,5,6,10b-tetrahydropyrazolo[5,1-*a*]isoquinolin-2(3*H*)-one (3ea)

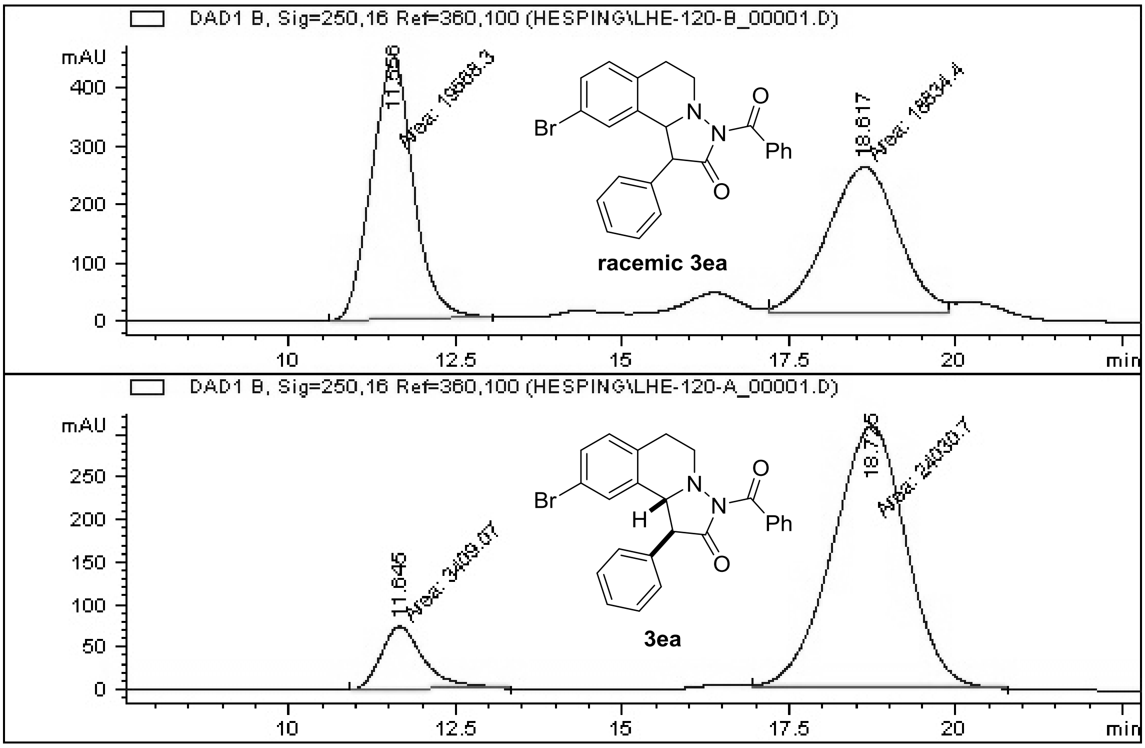

(1*S*,10b*R*)-3-Benzoyl-9-bromo-1-(naphthalen-2-yl)-1,5,6,10b-tetrahydropyrazolo[5,1-*a*]isoquinolin-2(3*H*)-one (3eh)

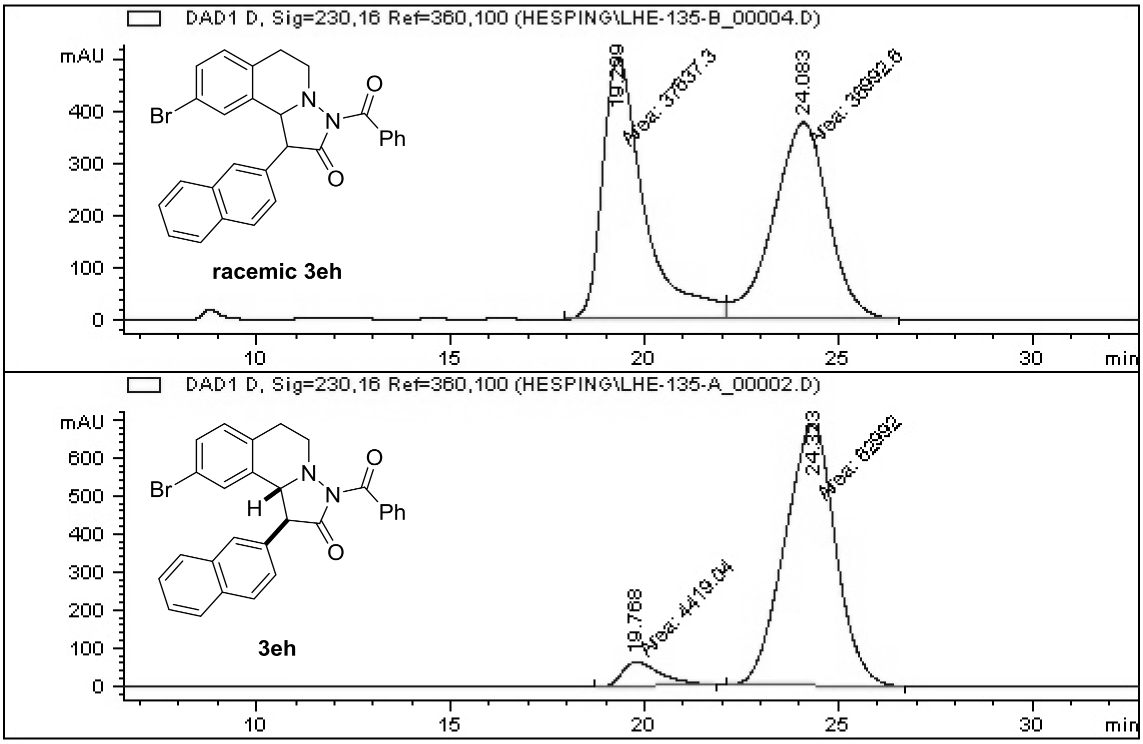

6. ^{1}H-, ^{13}C-, and ^{19}F-NMR spectra

1,7-Dimethoxyisochroman

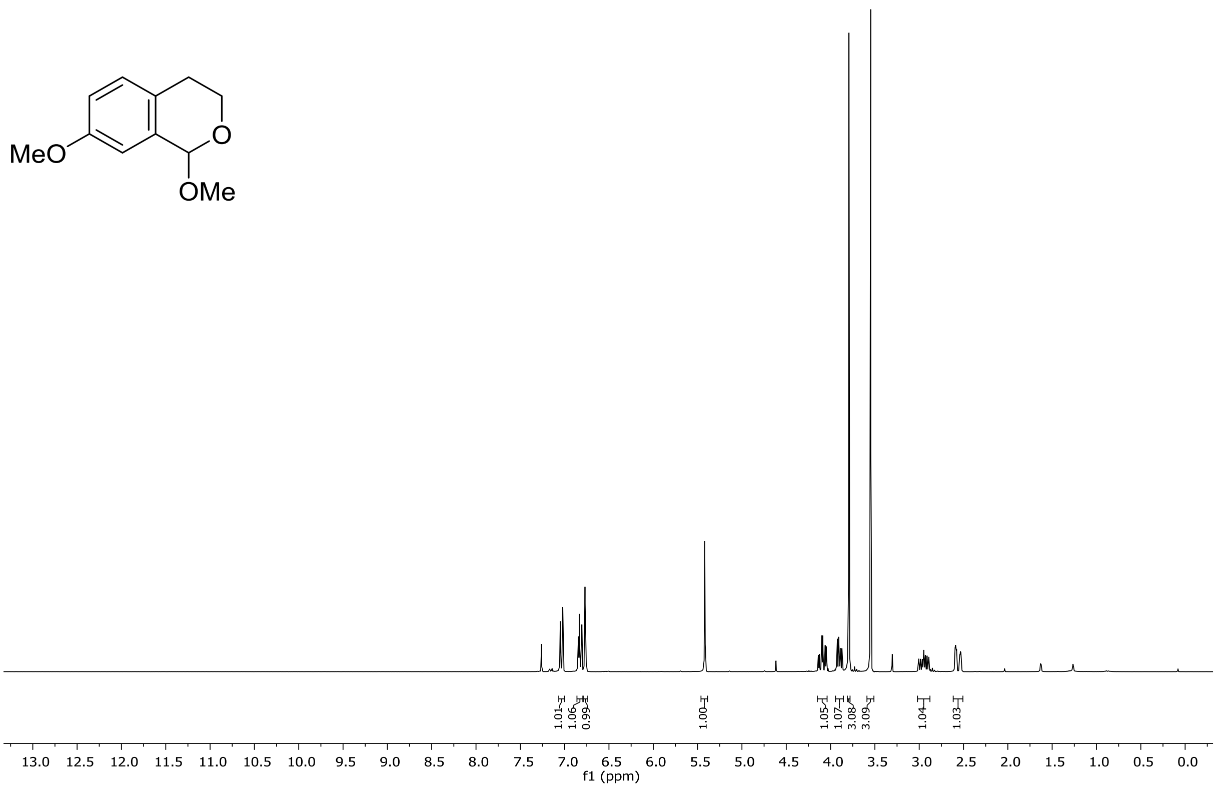

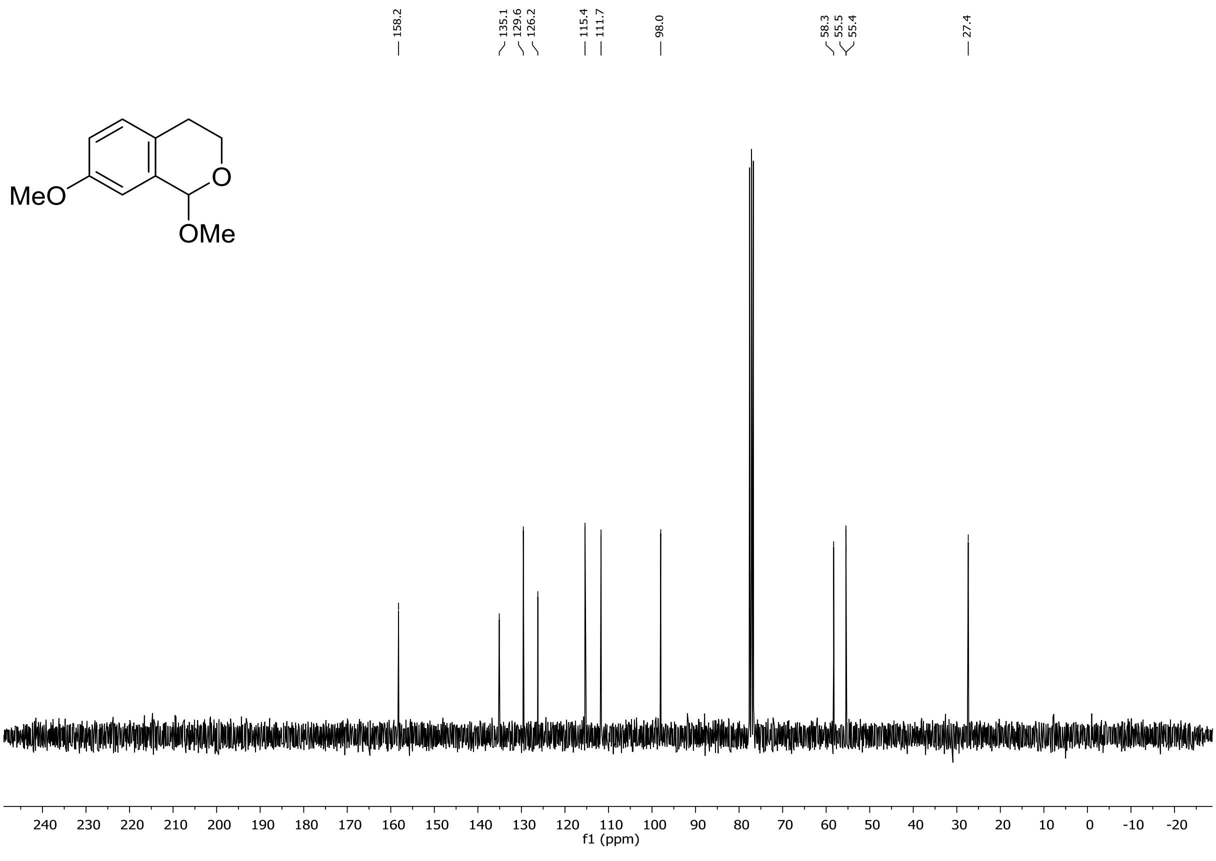

2-(2-Bromoethyl)-5-methoxybenzaldehyde

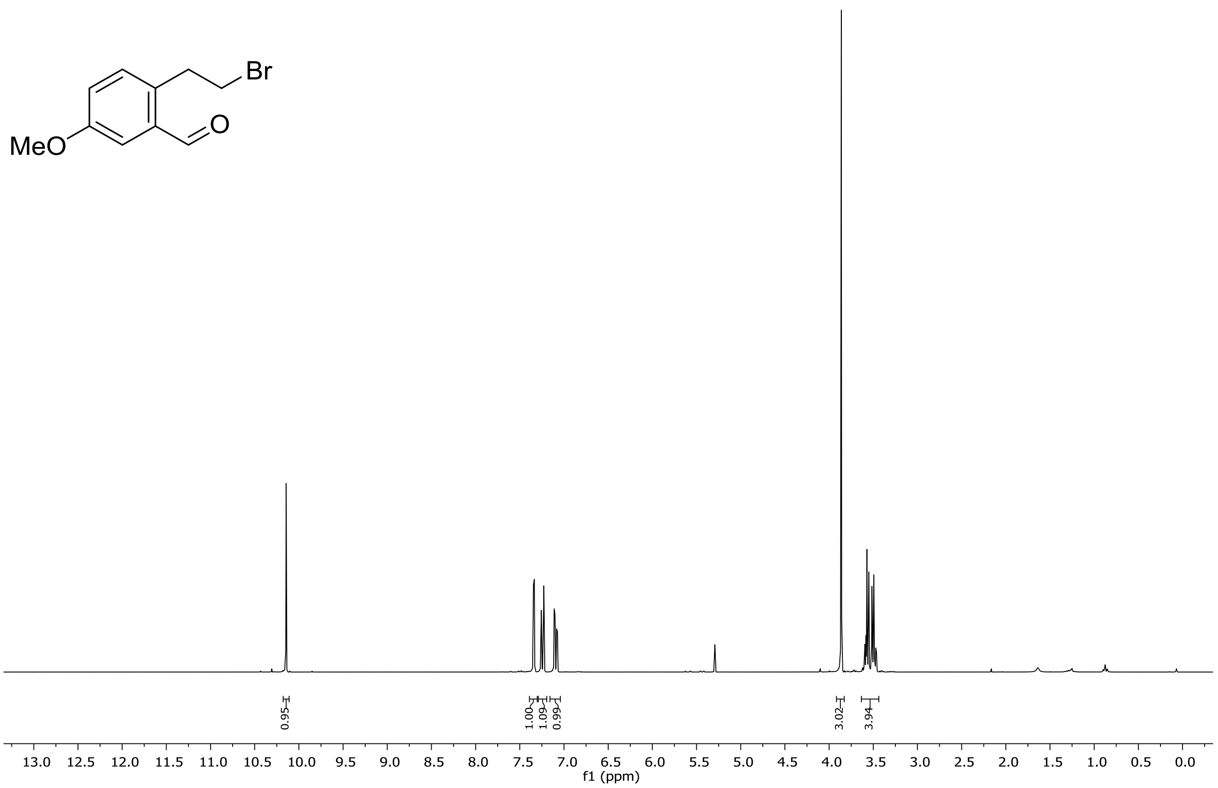

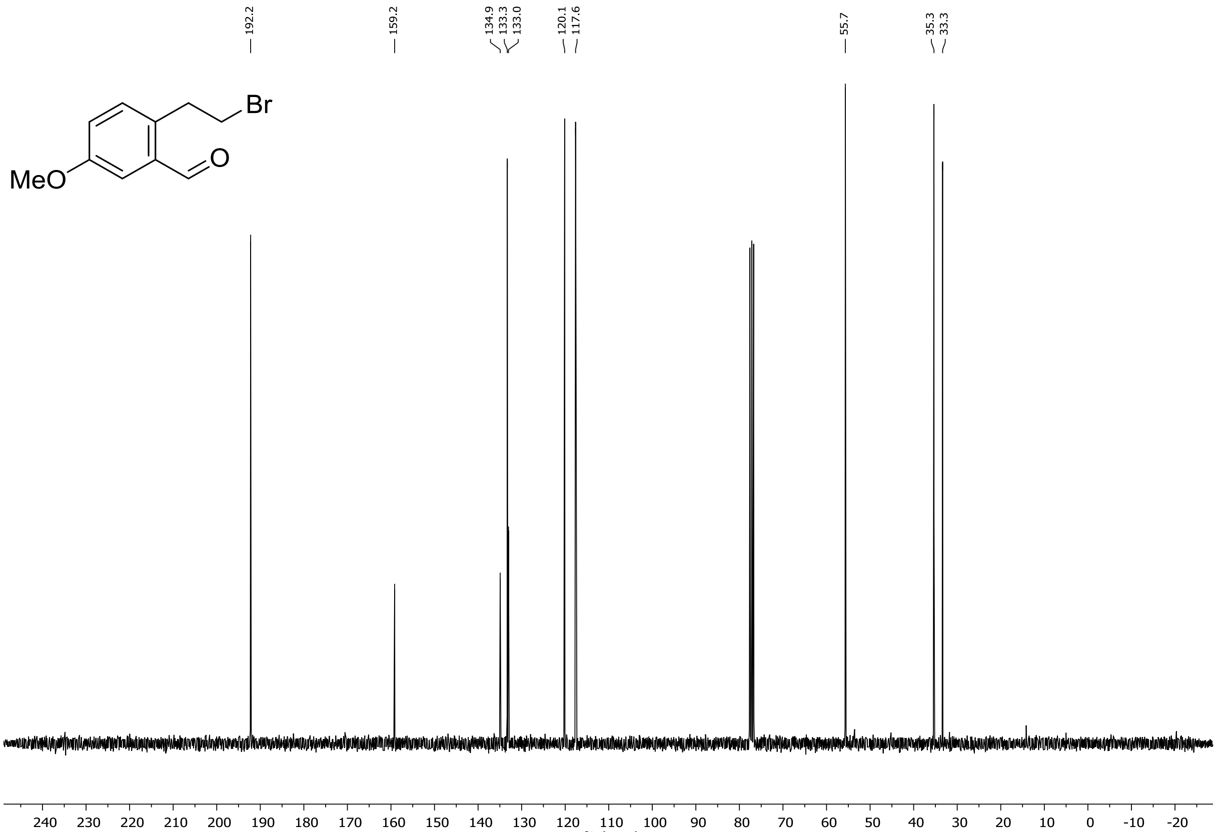

Benzoyl(7-methoxy-3,4-dihydroisoquinolin-2-ium-2-yl)amide (1d)

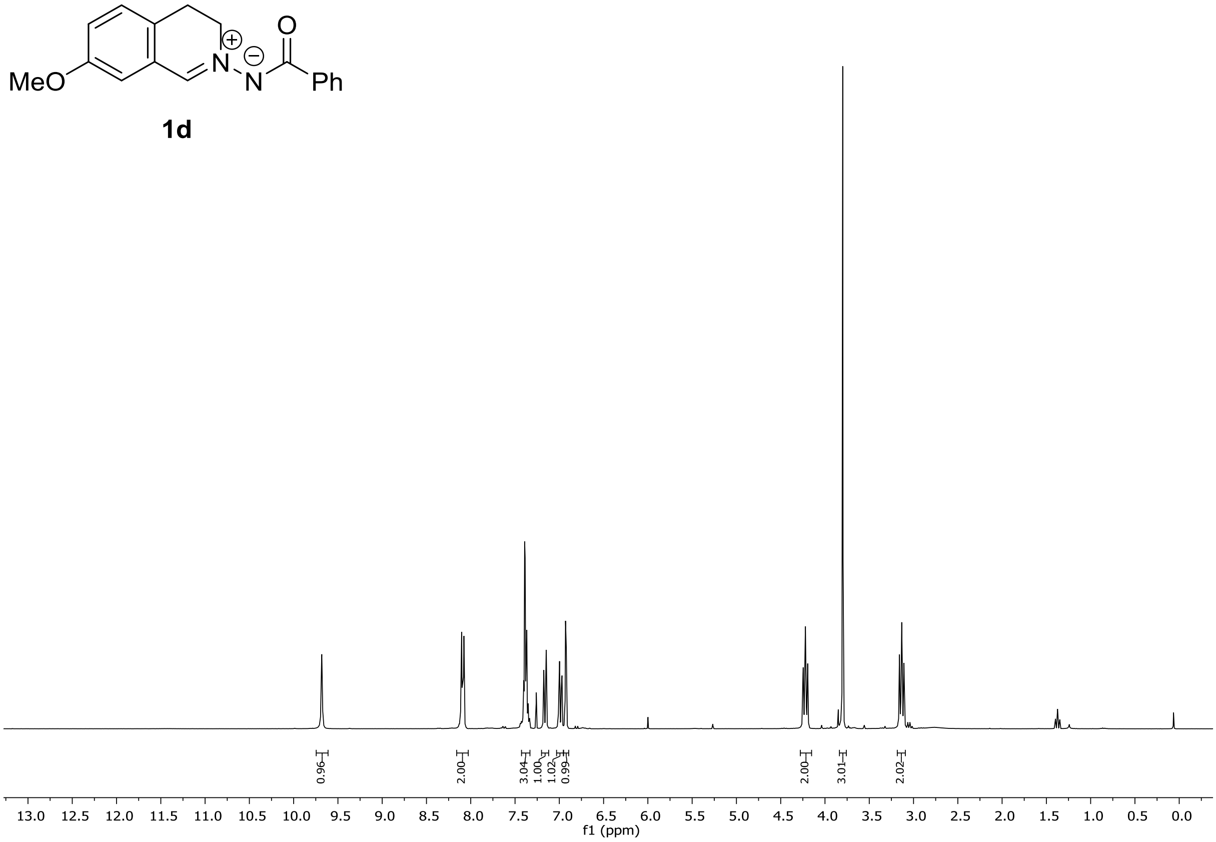

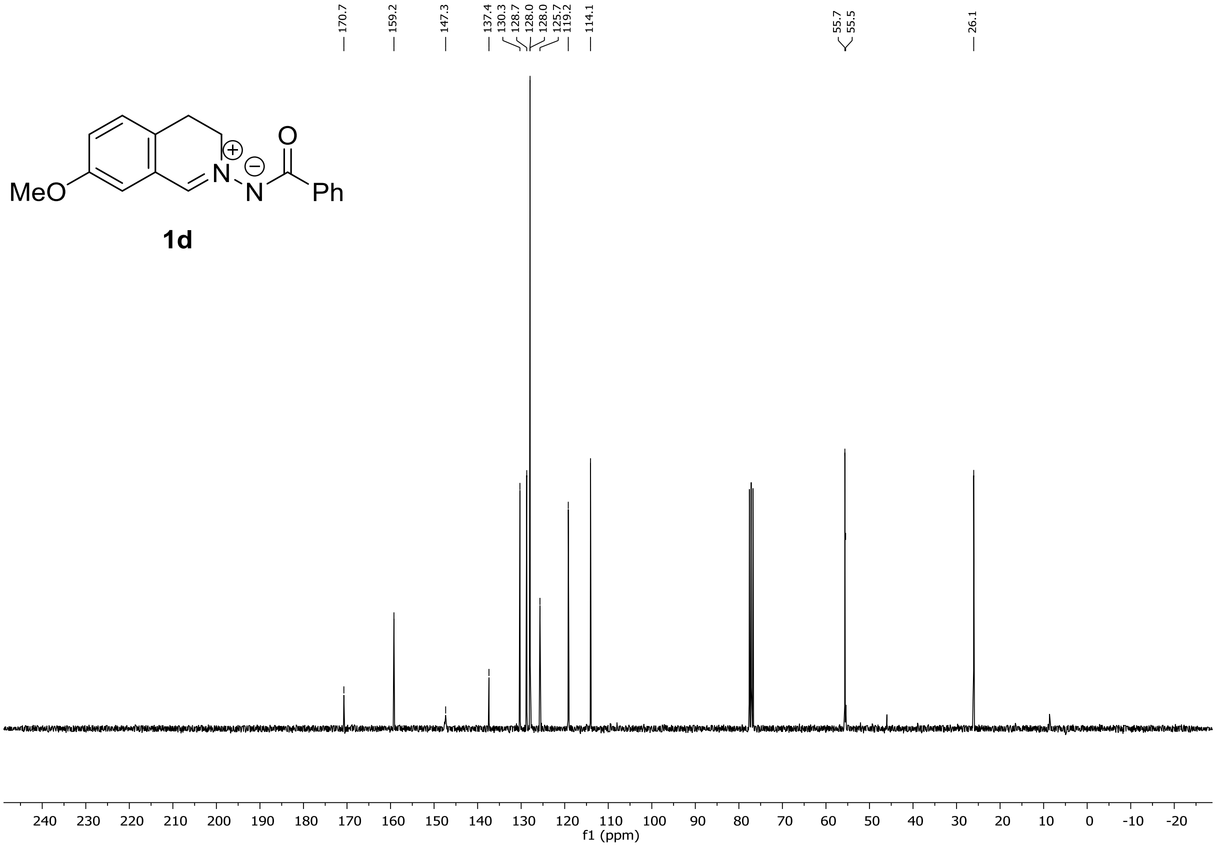

(1*S*,10b*R*)-3-Benzoyl-1-phenyl-1,5,6,10b-tetrahydropyrazolo[5,1-*a*]isoquinolin-2(3*H*)-one (3aa)

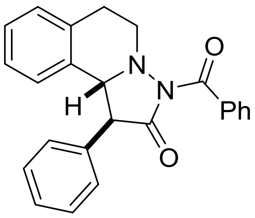

3aa

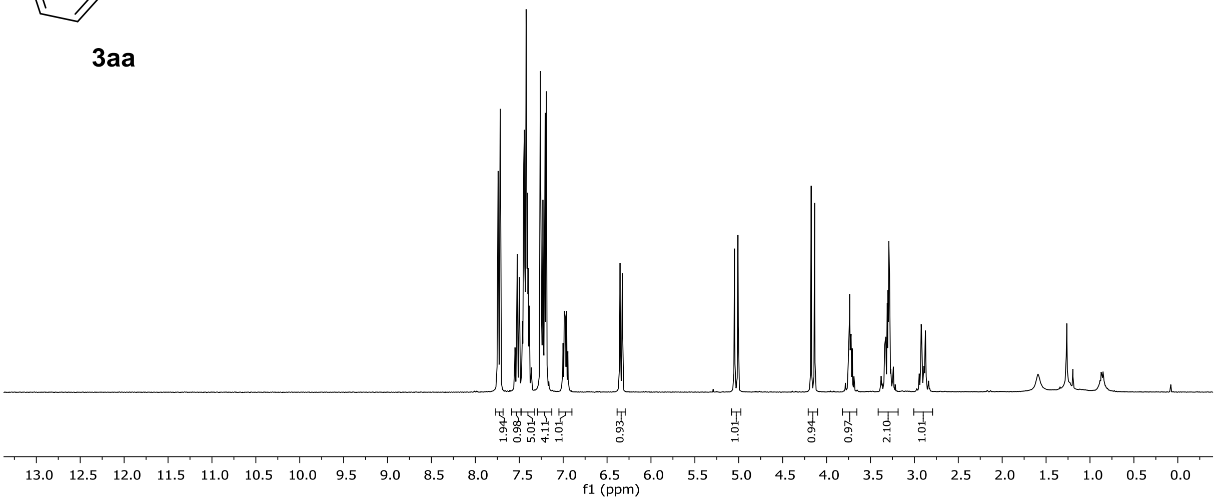

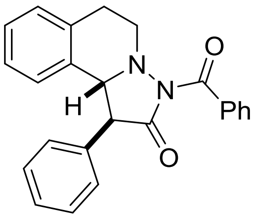

3aa

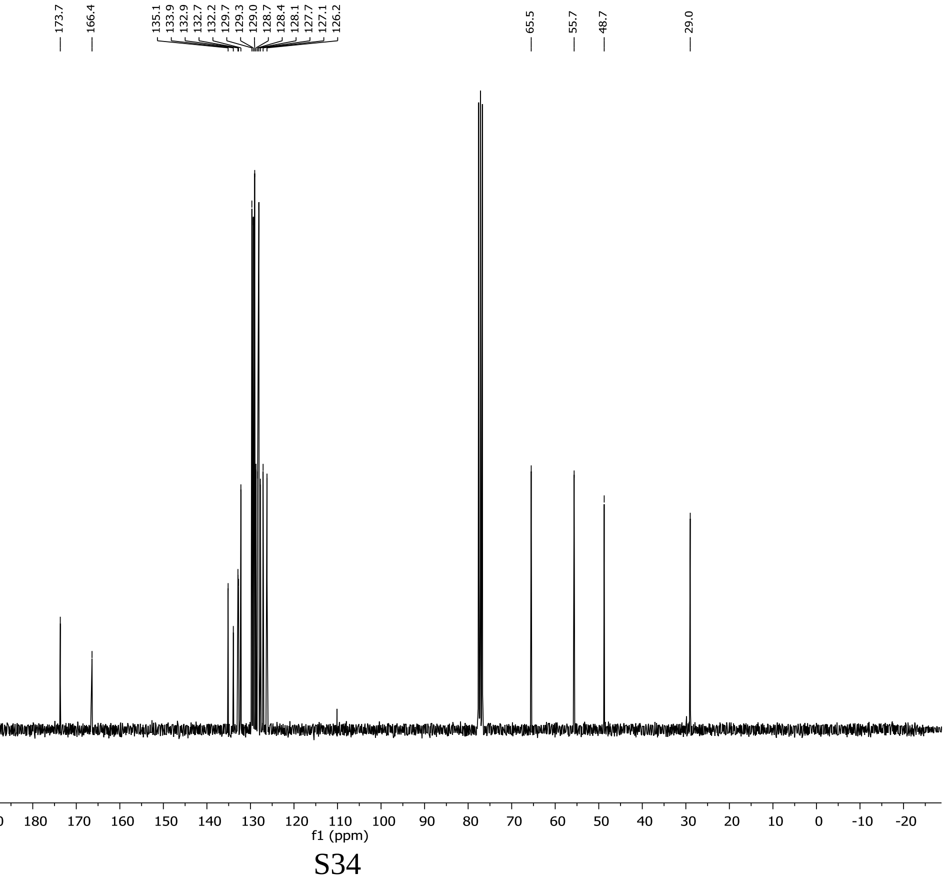

(1*S*,10b*R*)-3-Benzoyl-1-(*o*-tolyl)-1,5,6,10b-tetrahydropyrazolo[5,1-*a*]isoquinolin-2(3*H*)-one (3ab)

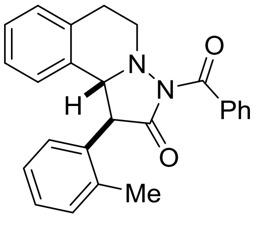

3ab

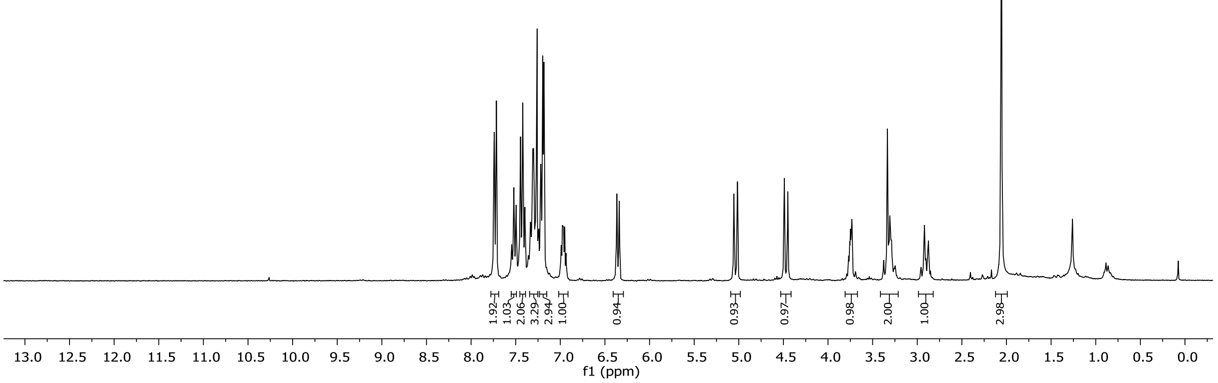

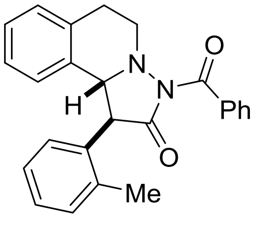

3ab

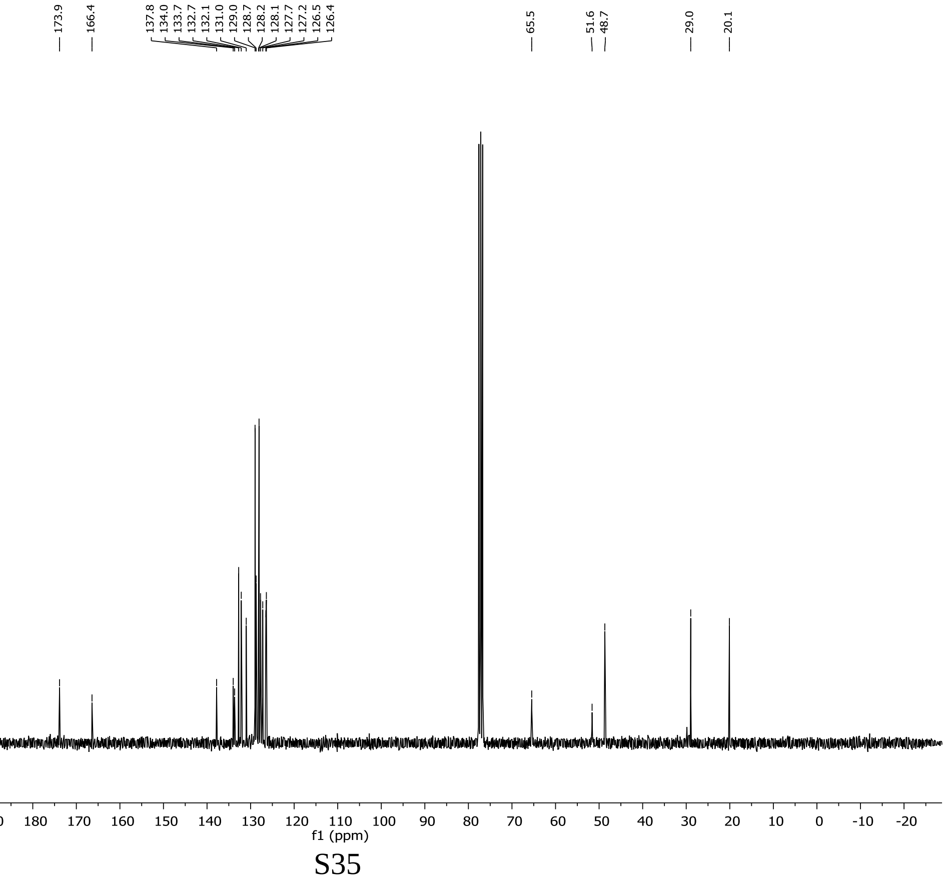

(1*S*,10b*R*)-3-Benzoyl-1-(4-methoxyphenyl)-1,5,6,10b-tetrahydropyrazolo[5,1-*a*]isoquinolin-2(3*H*)-one (3ac)

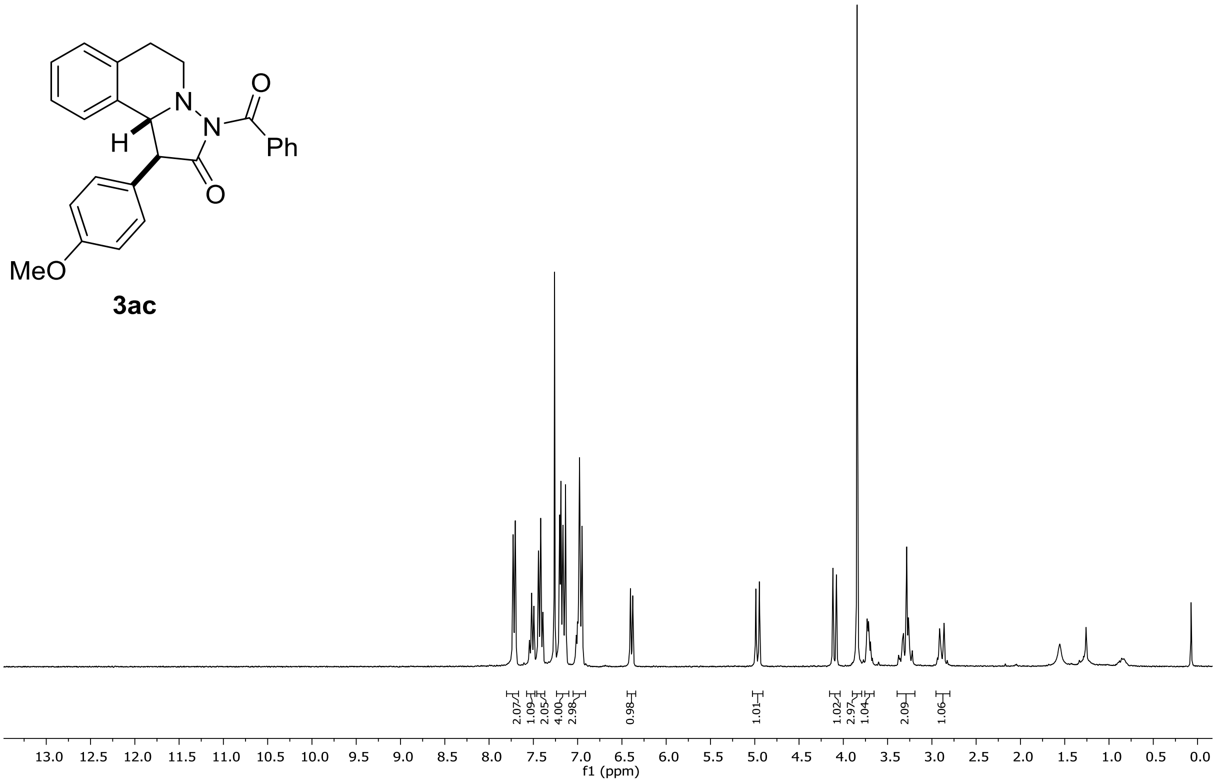

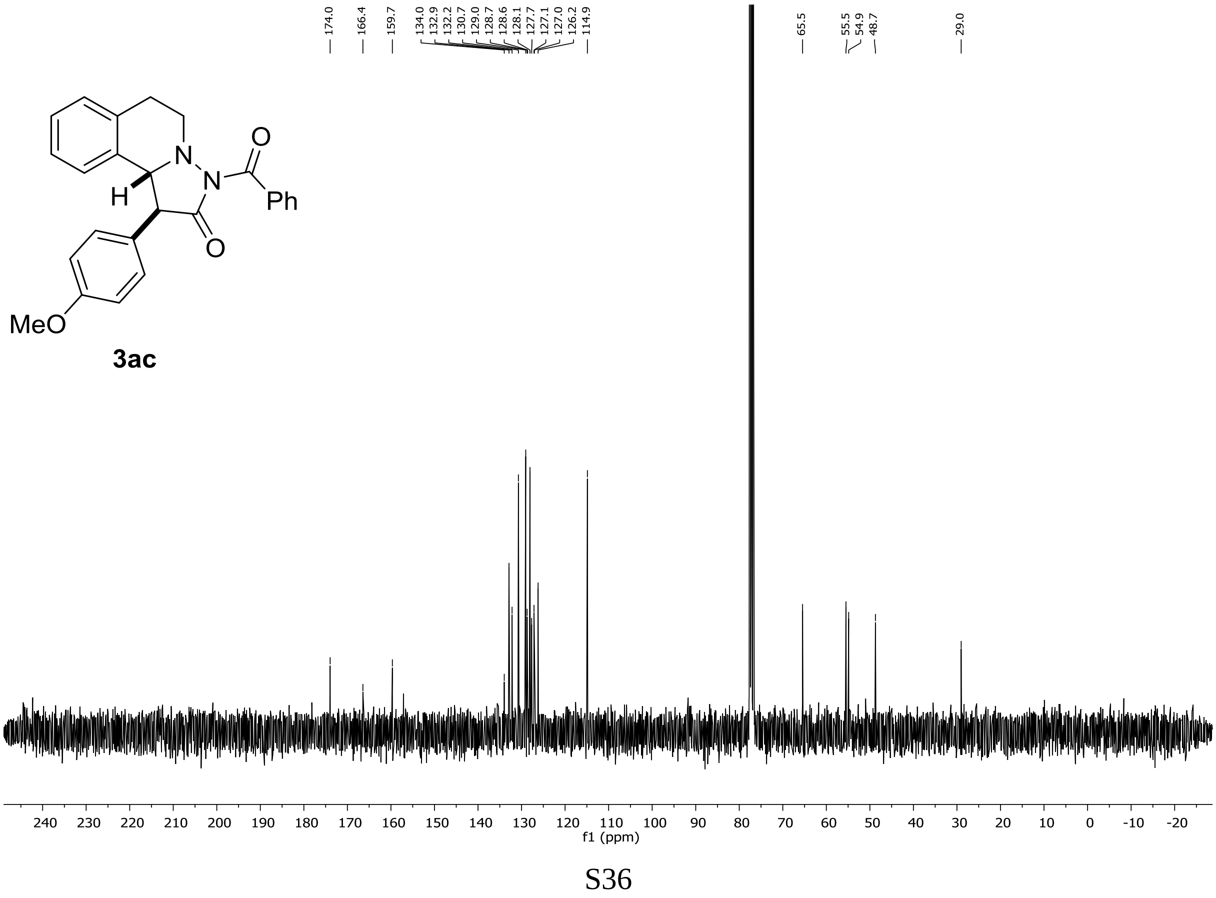

(1*S*,10b*R*)-3-Benzoyl-1-(4-chlorophenyl)-1,5,6,10b-tetrahydropyrazolo[5,1-*a*]isoquinolin-2(3*H*)-one (3ad)

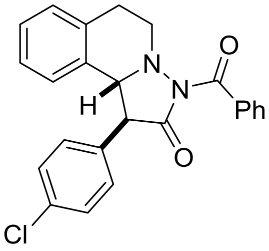

3ad

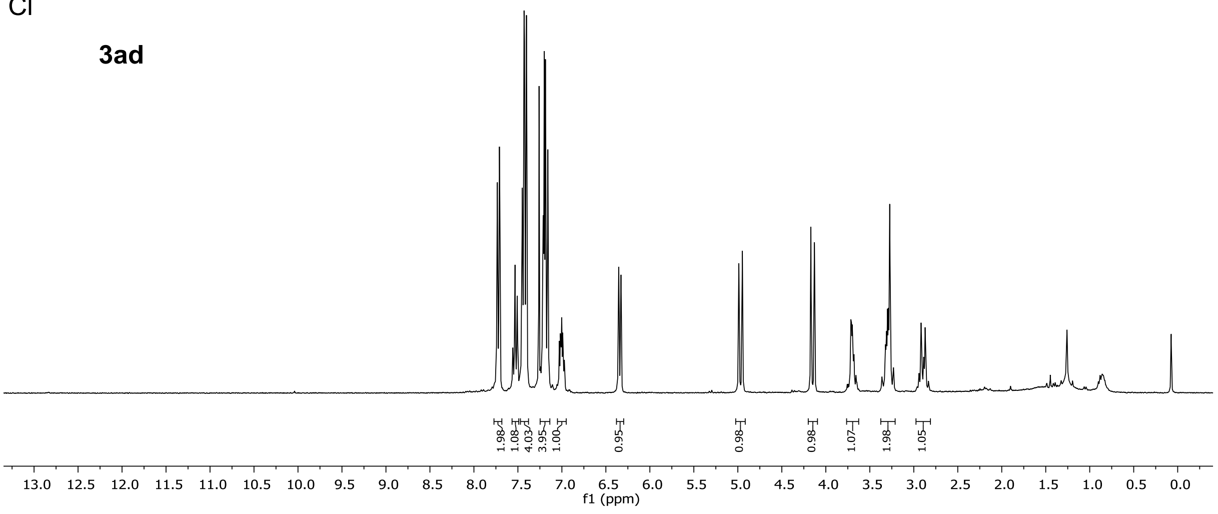

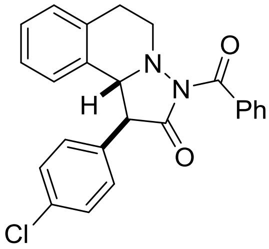

3ad

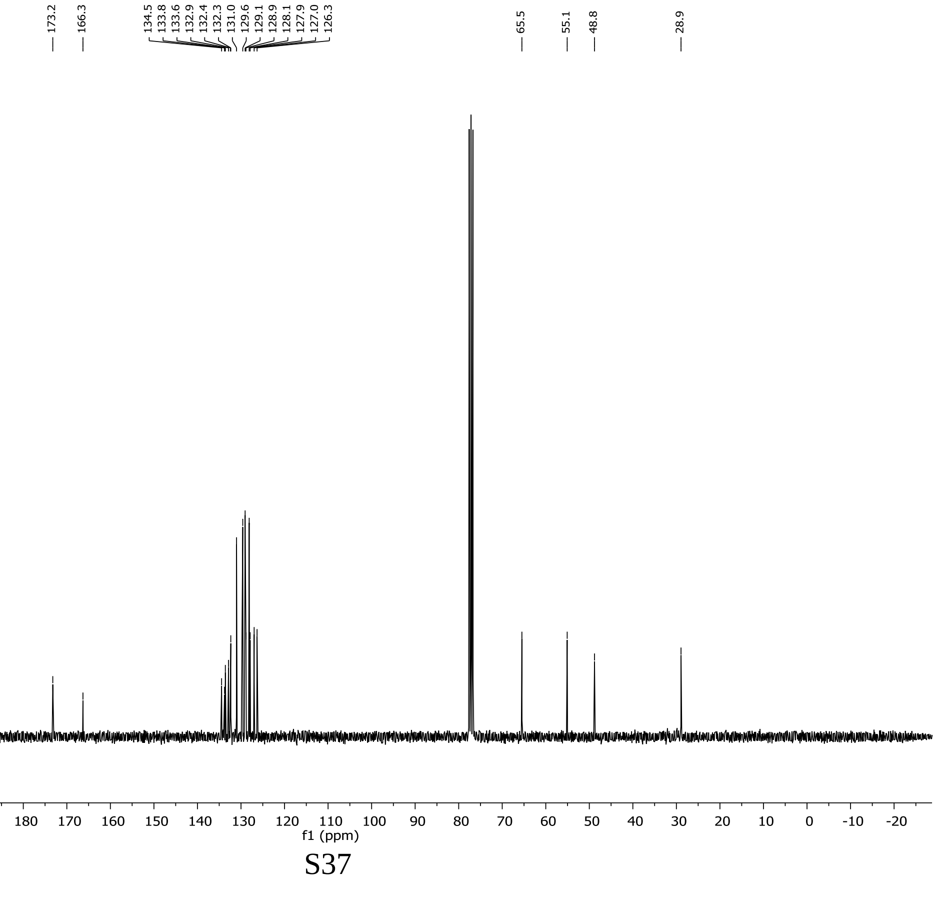

(1*S*,10b*R*)-3-Benzoyl-1-(3-chlorophenyl)-1,5,6,10b-tetrahydropyrazolo[5,1-*a*]isoquinolin-2(3*H*)-one (3ae)

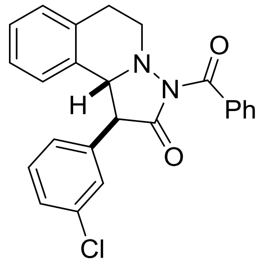

3ae

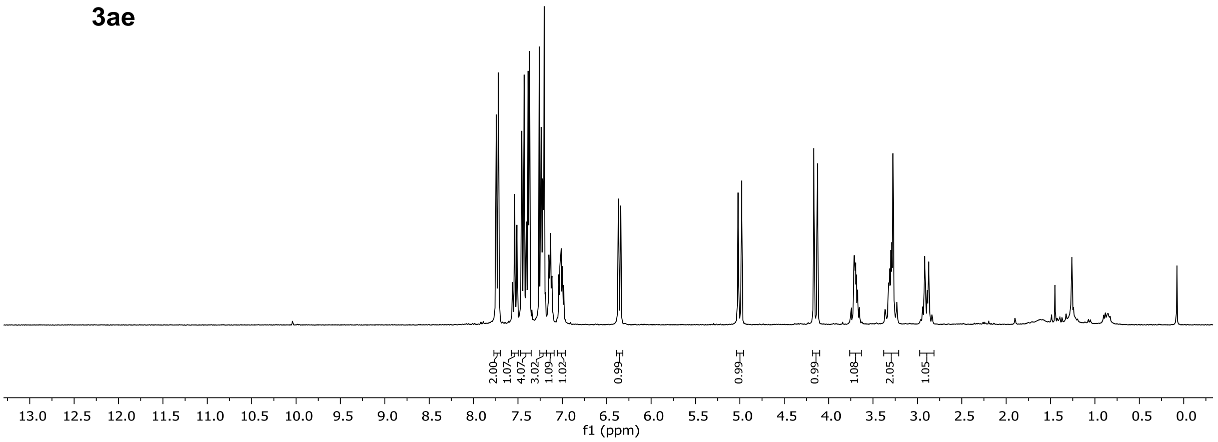

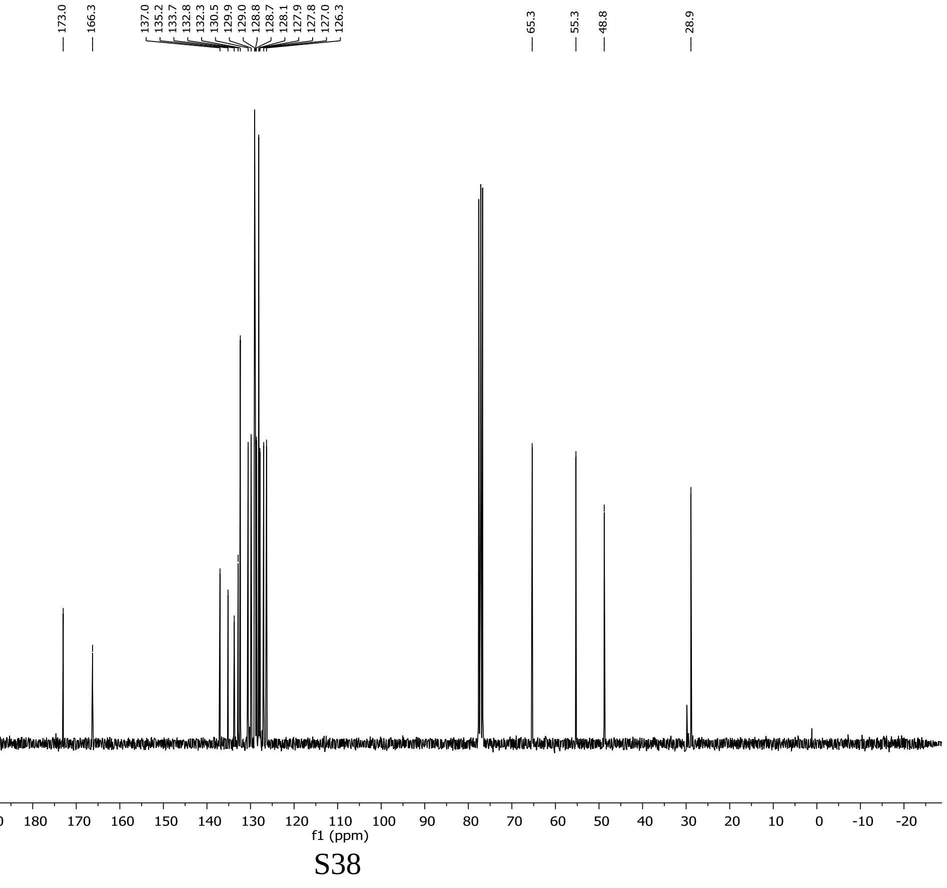

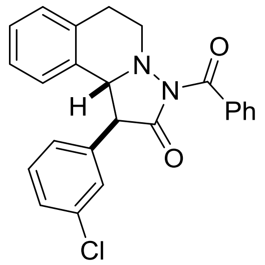

3ae

(1*S*,10b*R*)-3-Benzoyl-1-(2-fluorophenyl)-1,5,6,10b-tetrahydropyrazolo[5,1-*a*]isoquinolin-2(3*H*)-one (3af)

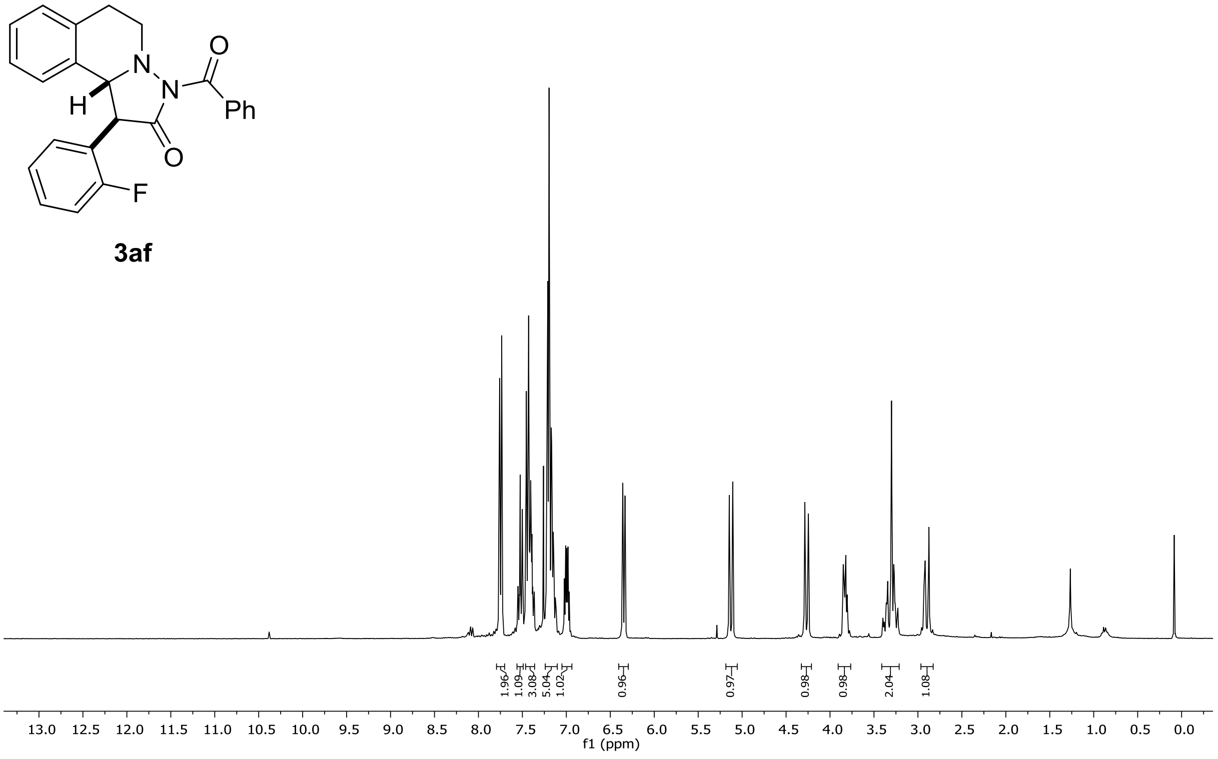

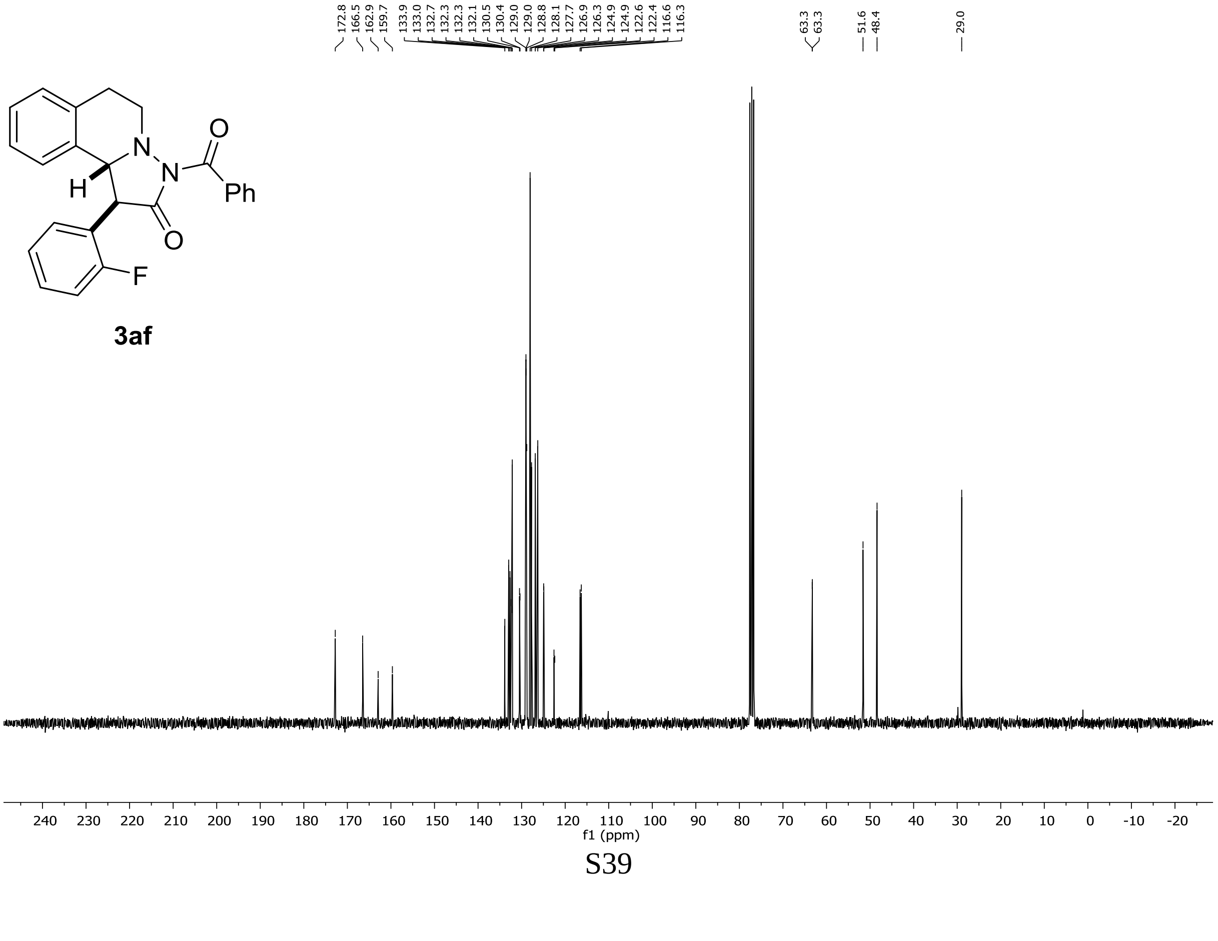

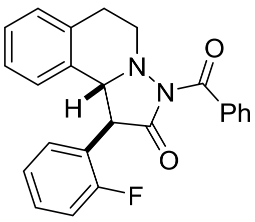

3af

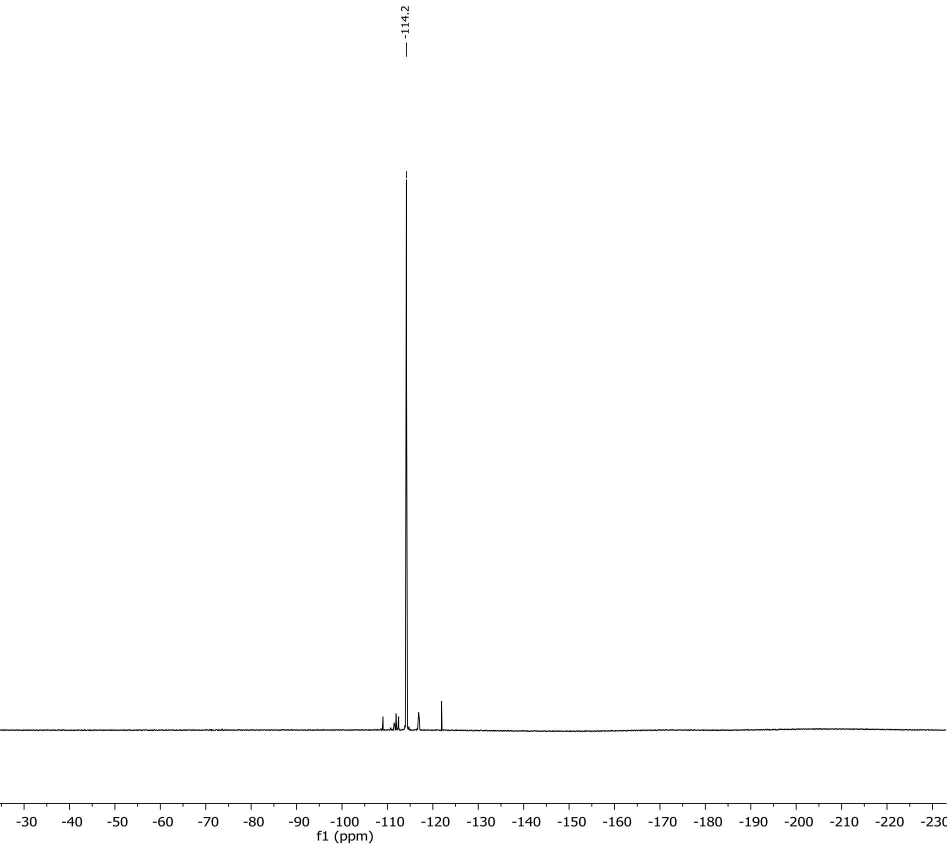

(1*S*,10b*R*)-3-Benzoyl-1-(4-(trifluoromethyl)phenyl)-1,5,6,10b-tetrahydropyrazolo[5,1-*a*]isoquinolin-2(3*H*)-one (3ag)

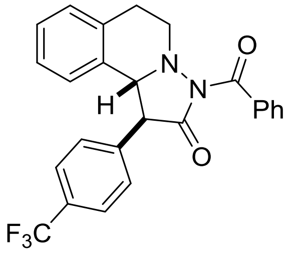

3ag

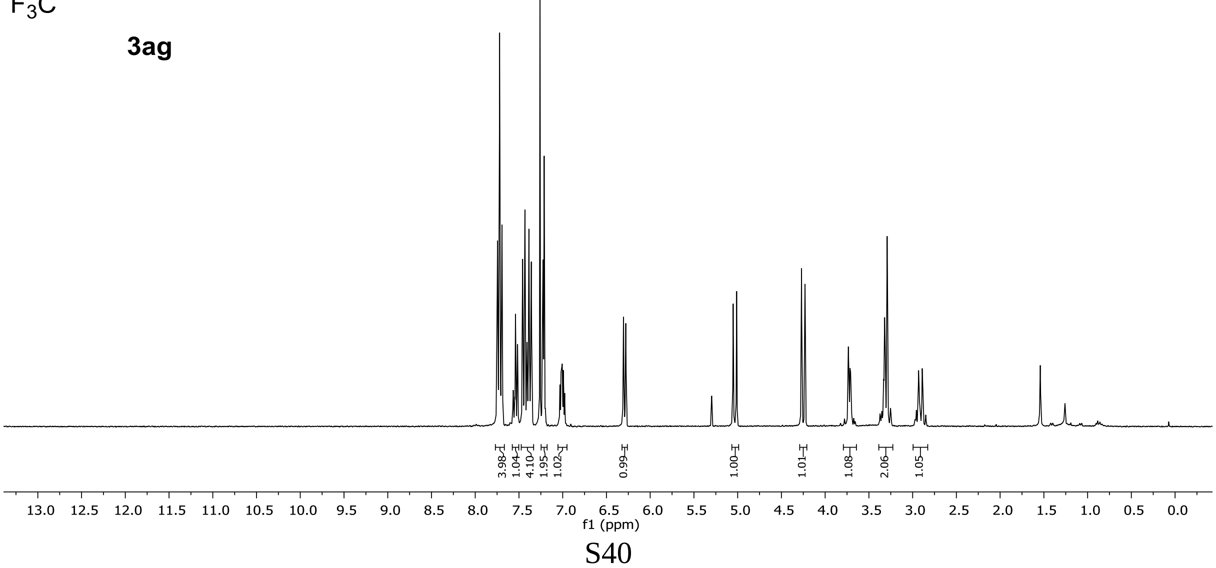

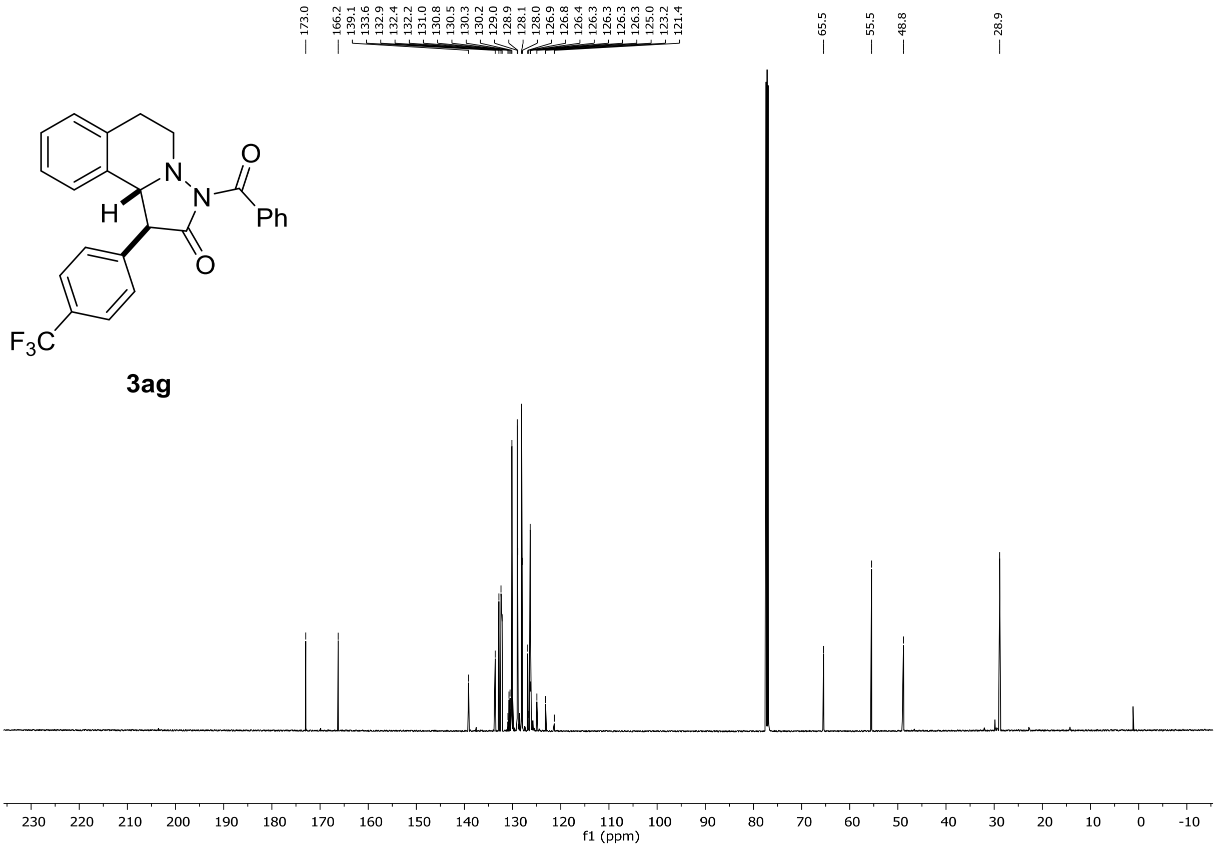

173.0
166.2
139.1
133.6
132.9
132.4
132.2
131.0
130.8
130.5
130.3
130.2
129.0
128.9
128.1
128.0
126.9
126.8
126.4
126.3
126.3
126.3
125.0
123.2
121.4
65.5
55.5
48.8
28.9
N
N
O
Ph
H
O
F3C
3ag
230 220 210 200 190 180 170 160 150 140 130 120 110 100 90 80 70 60 50 40 30 20 10 0 -10
f1 (ppm)

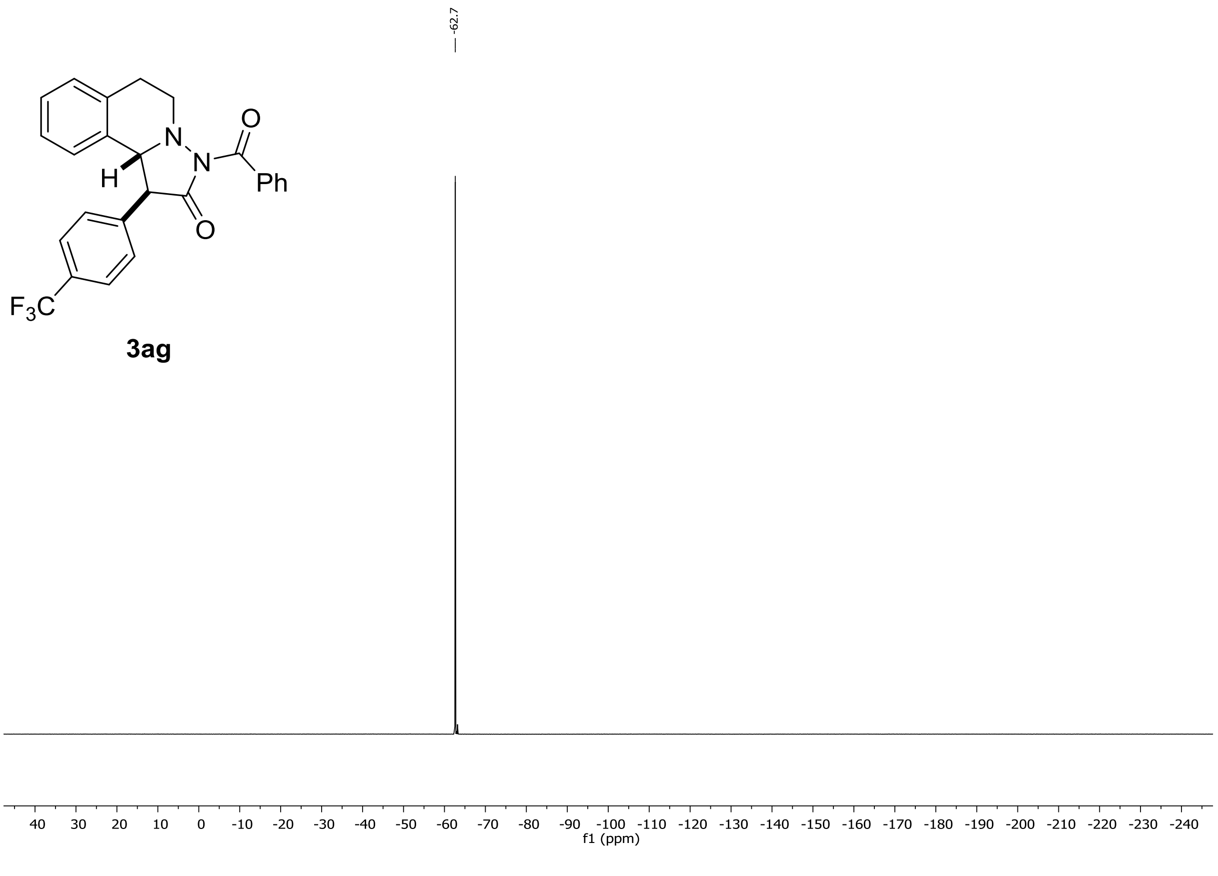

-62.7
N
N
O
Ph
H
O
F3C
3ag
40 30 20 10 0 -10 -20 -30 -40 -50 -60 -70 -80 -90 -100 -110 -120 -130 -140 -150 -160 -170 -180 -190 -200 -210 -220 -230 -240
f1 (ppm)

(1*S*,10b*R*)-3-Benzoyl-1-(naphthalen-2-yl)-1,5,6,10b-tetrahydropyrazolo[5,1-*a*]isoquinolin-2(3*H*)-one (3ah)

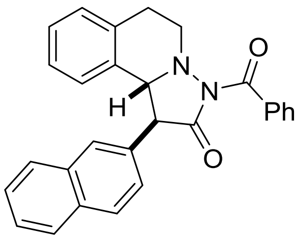

3ah

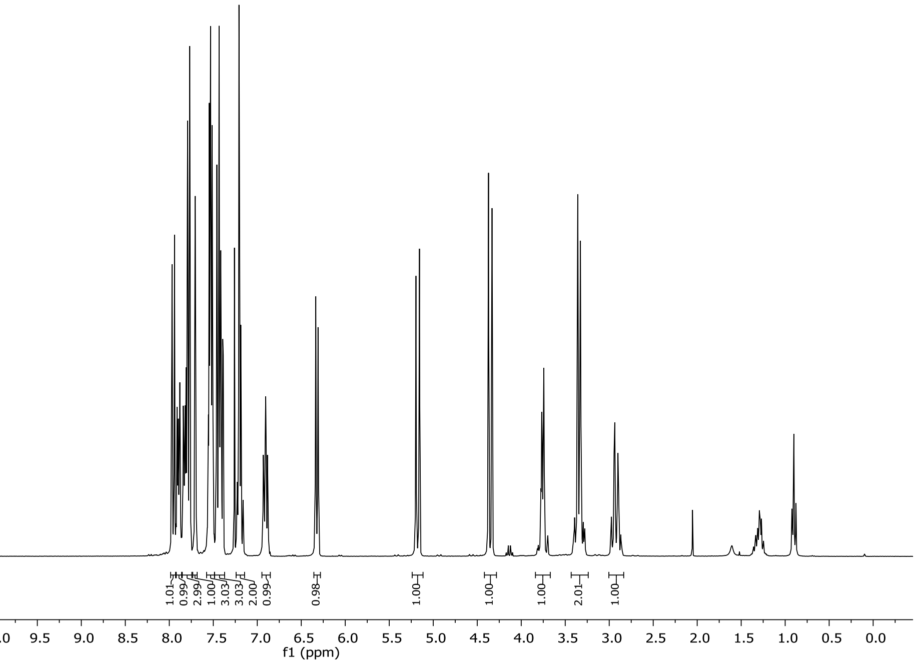

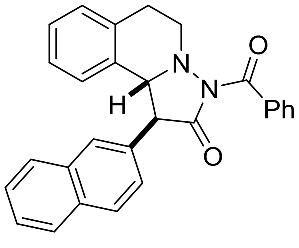

3ah

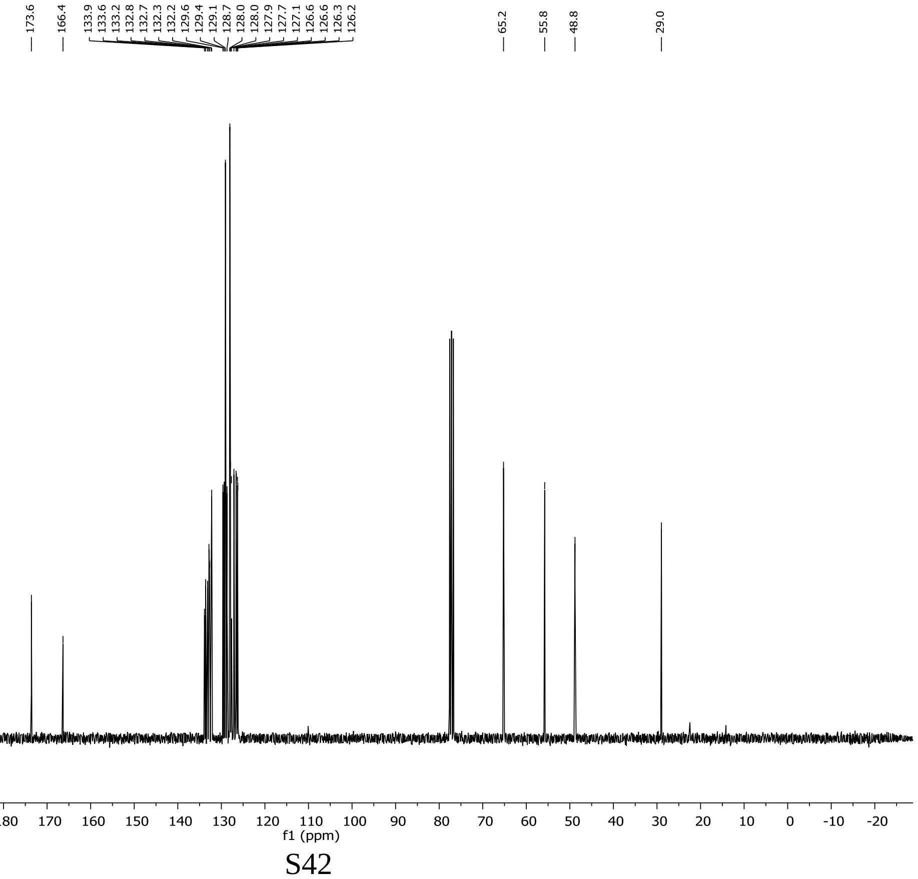

(1*S*,10b*R*)-3-Benzoyl-1-(thiophen-2-yl)-1,5,6,10b-tetrahydropyrazolo[5,1-*a*]isoquinolin-2(3*H*)-one (3ai)

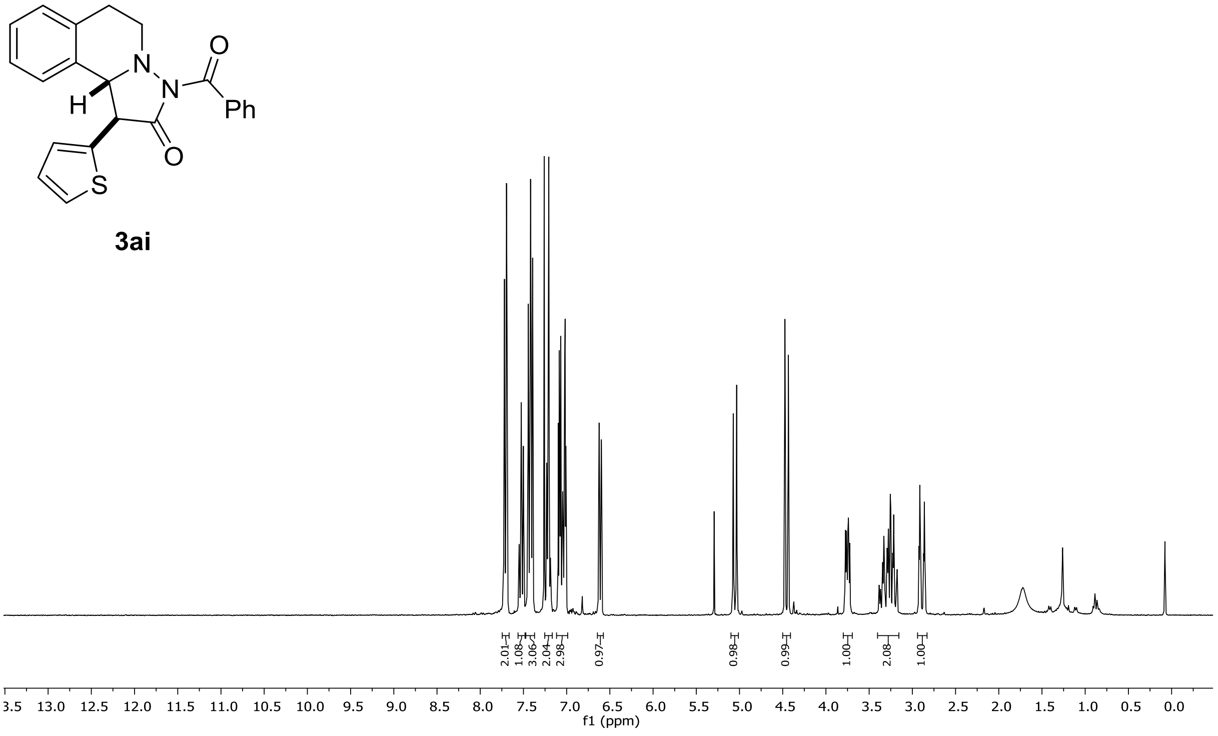

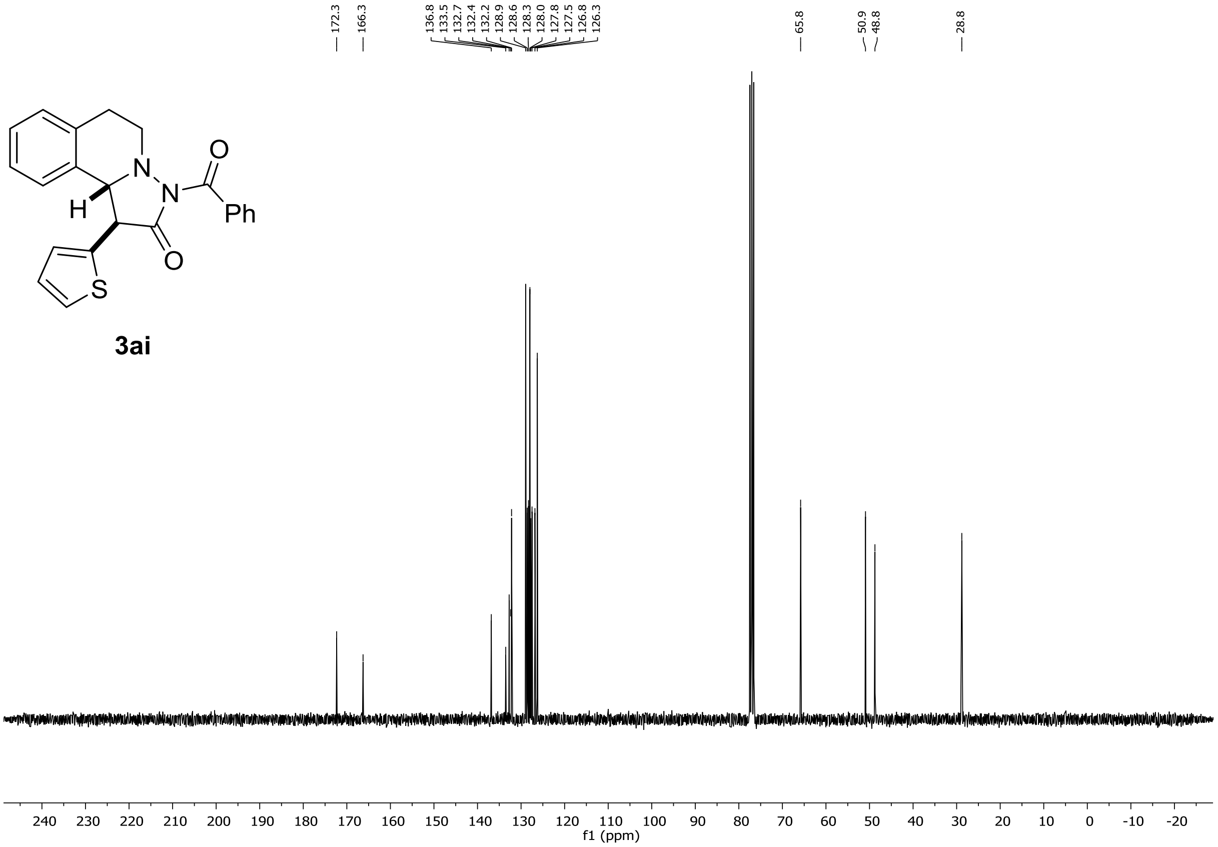

(1*S*,10b*R*)-3-Benzoyl-7-methyl-1-phenyl-1,5,6,10b-tetrahydropyrazolo[5,1-*a*]isoquinolin-2(3*H*)-one (3ba)

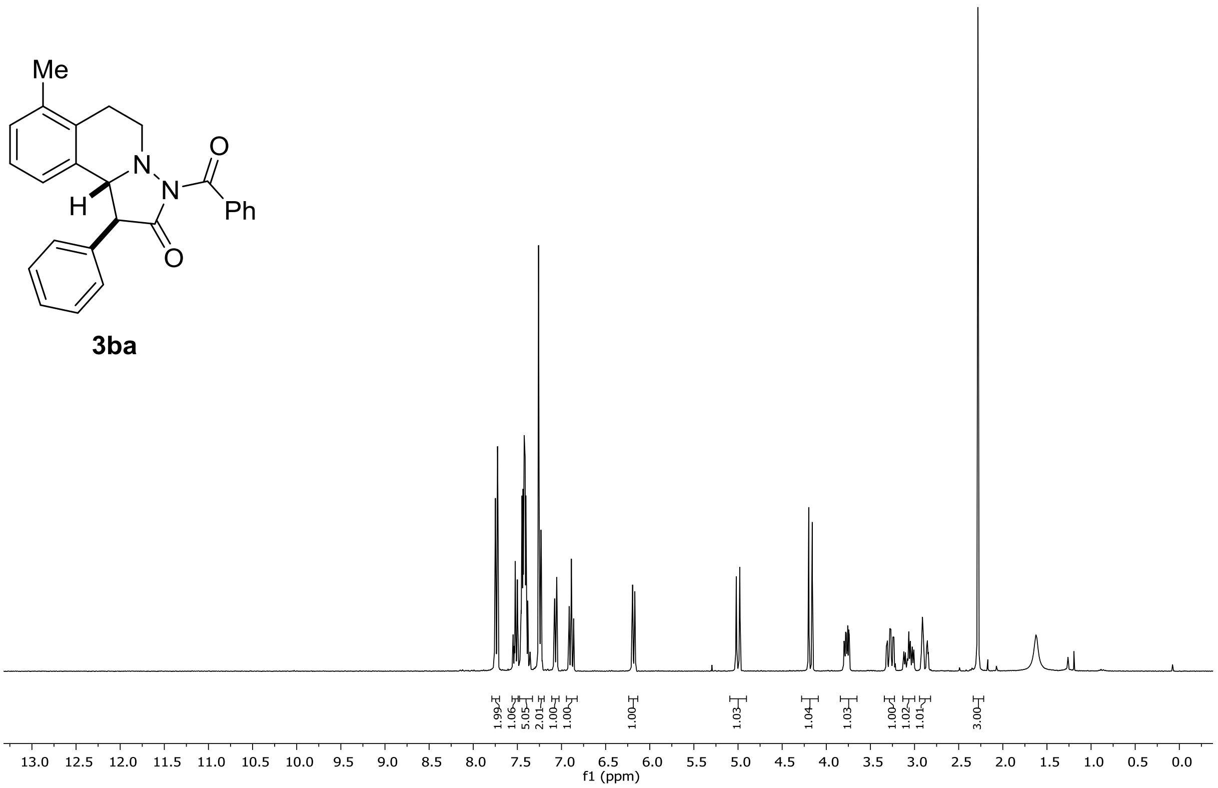

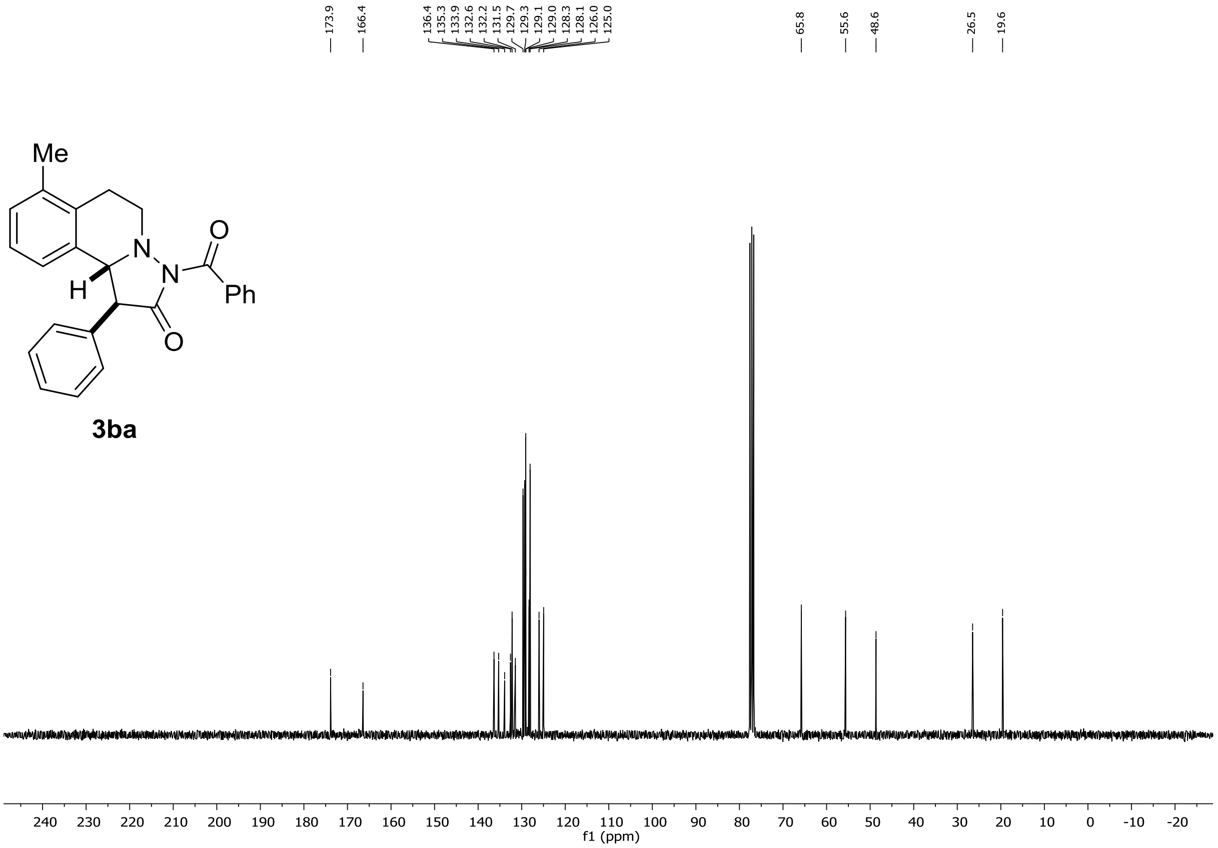

(1*S*,10b*R*)-3-Benzoyl-7-methyl-1-(naphthalen-2-yl)-1,5,6,10b-tetrahydropyrazolo[5,1-*a*]isoquinolin-2(3*H*)-one (3bh)

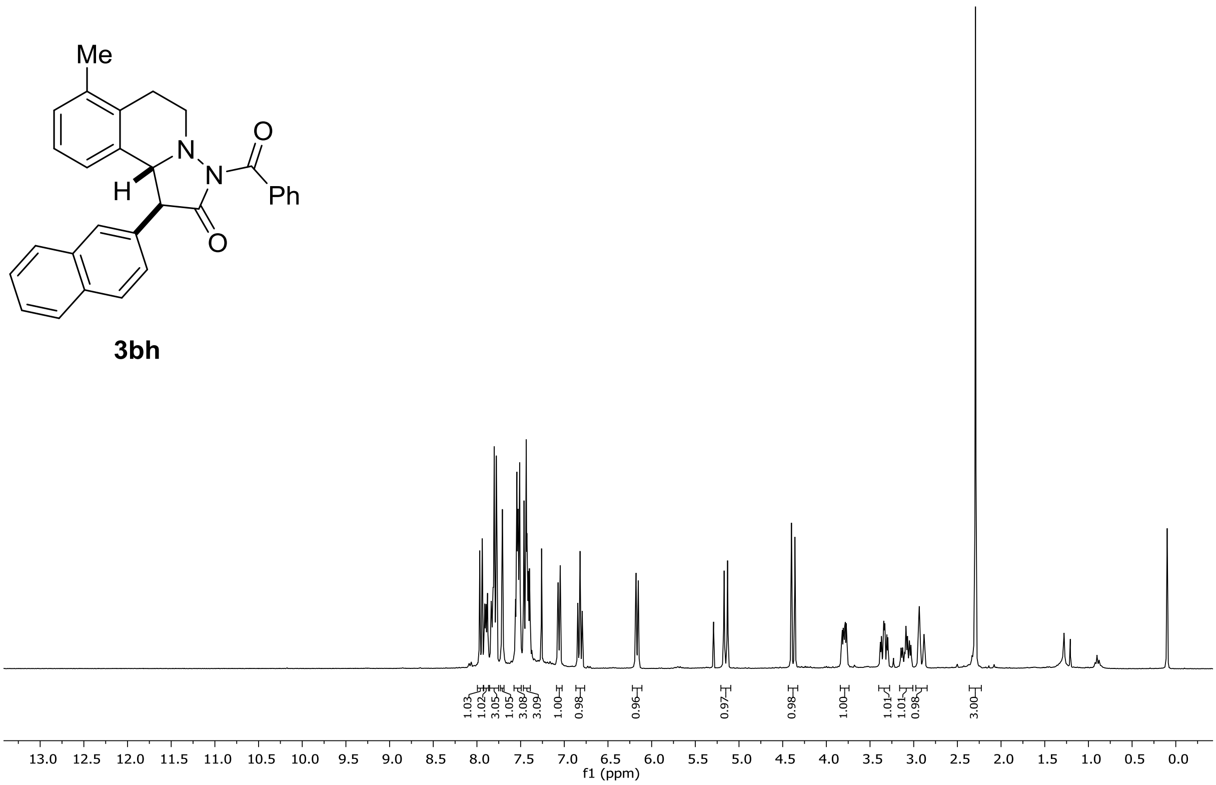

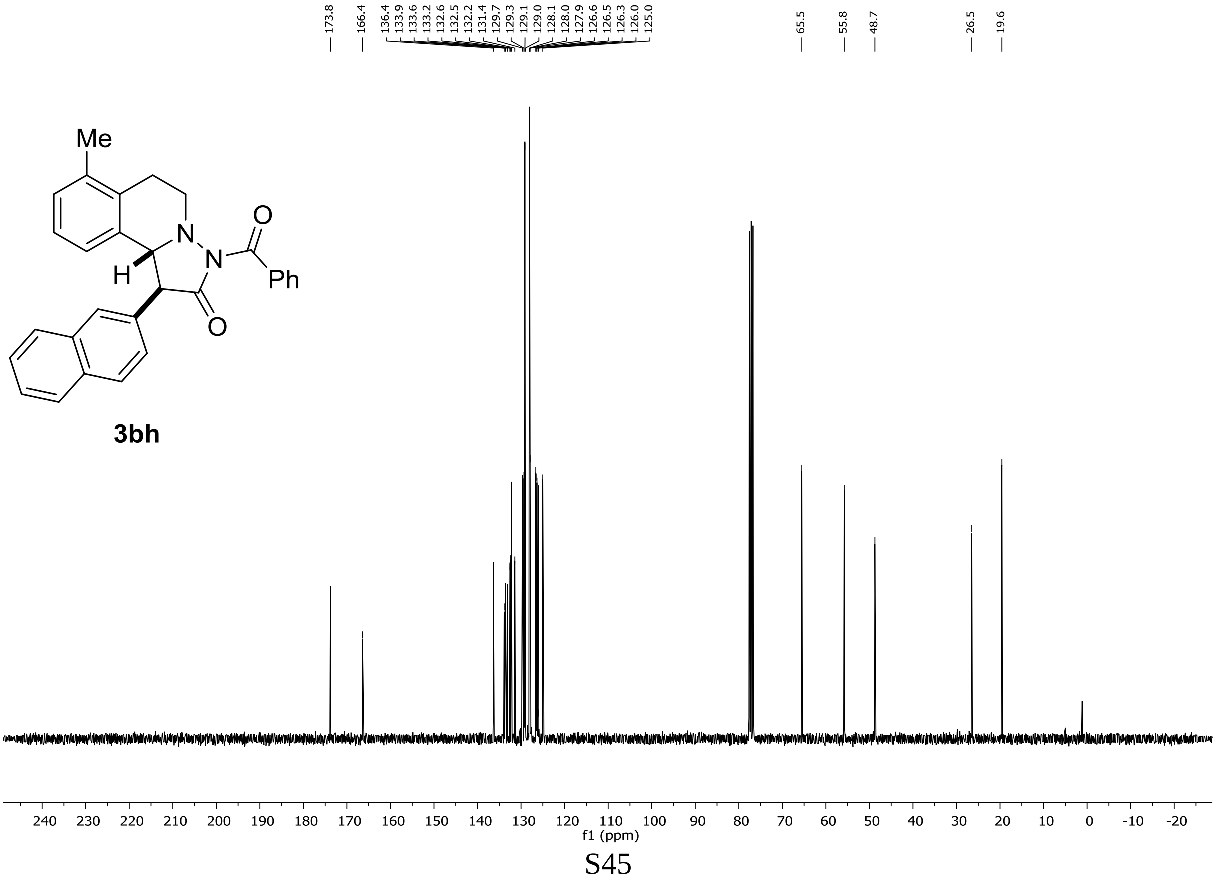

(1*S*,10b*R*)-3-Benzoyl-10-methyl-1-phenyl-1,5,6,10b-tetrahydropyrazolo[5,1-*a*]isoquinolin-2(3*H*)-one (3ca)

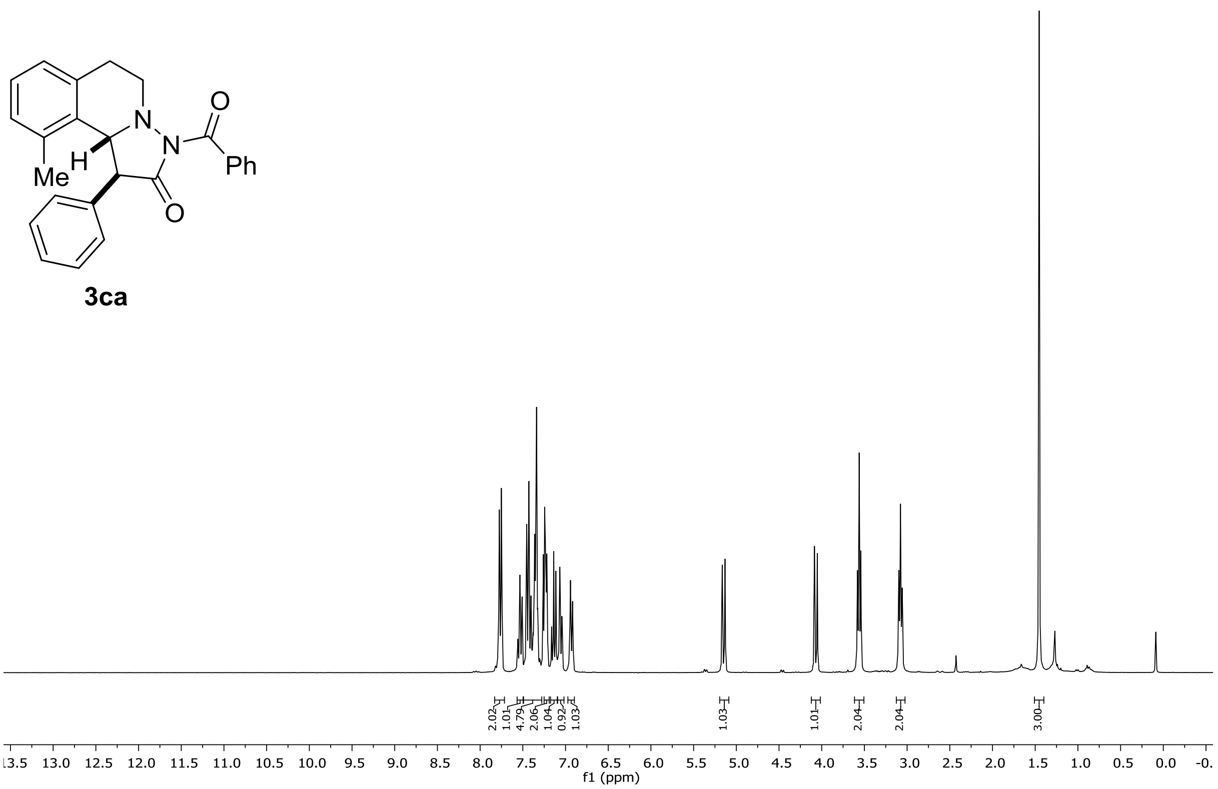

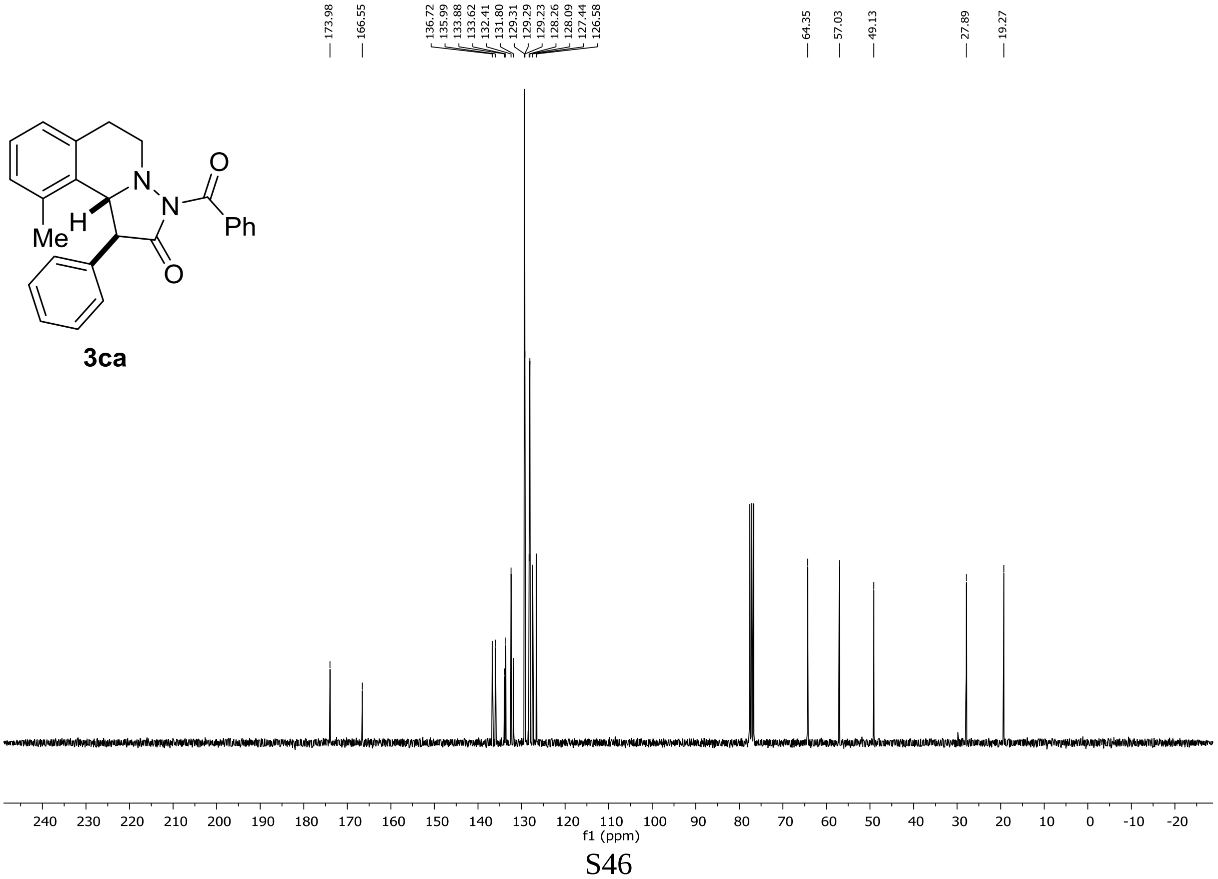

(1*S*,10b*R*)-3-Benzoyl-9-bromo-1-(naphthalen-2-yl)-1,5,6,10b-tetrahydropyrazolo[5,1-*a*]isoquinolin-2(3*H*)-one (3ch)

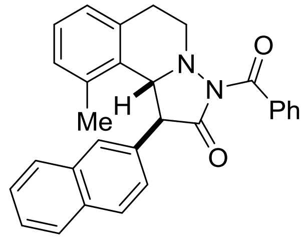

3ch

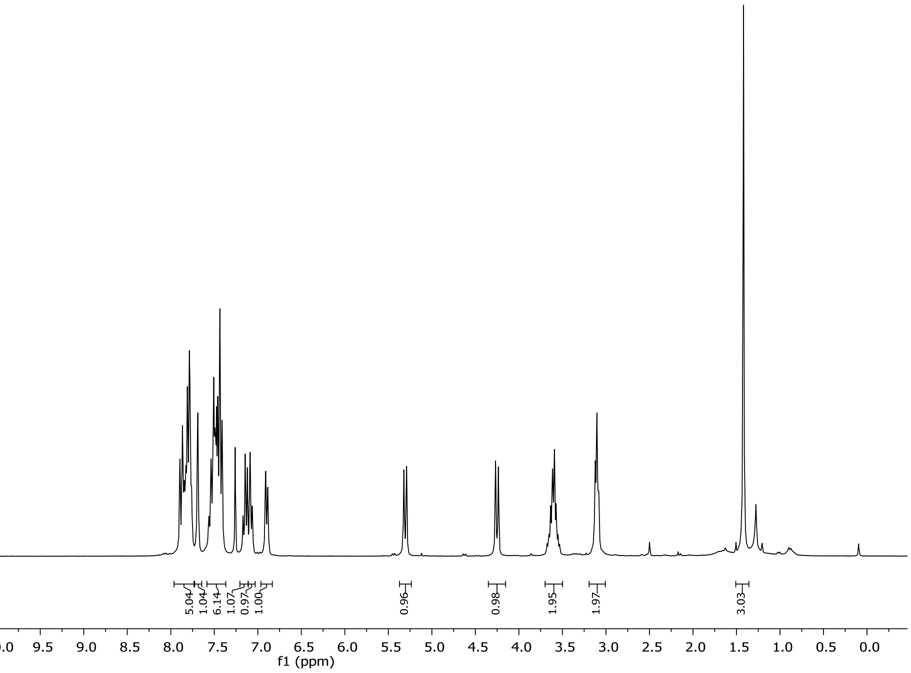

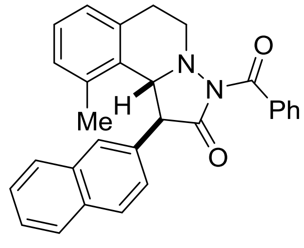

3ch

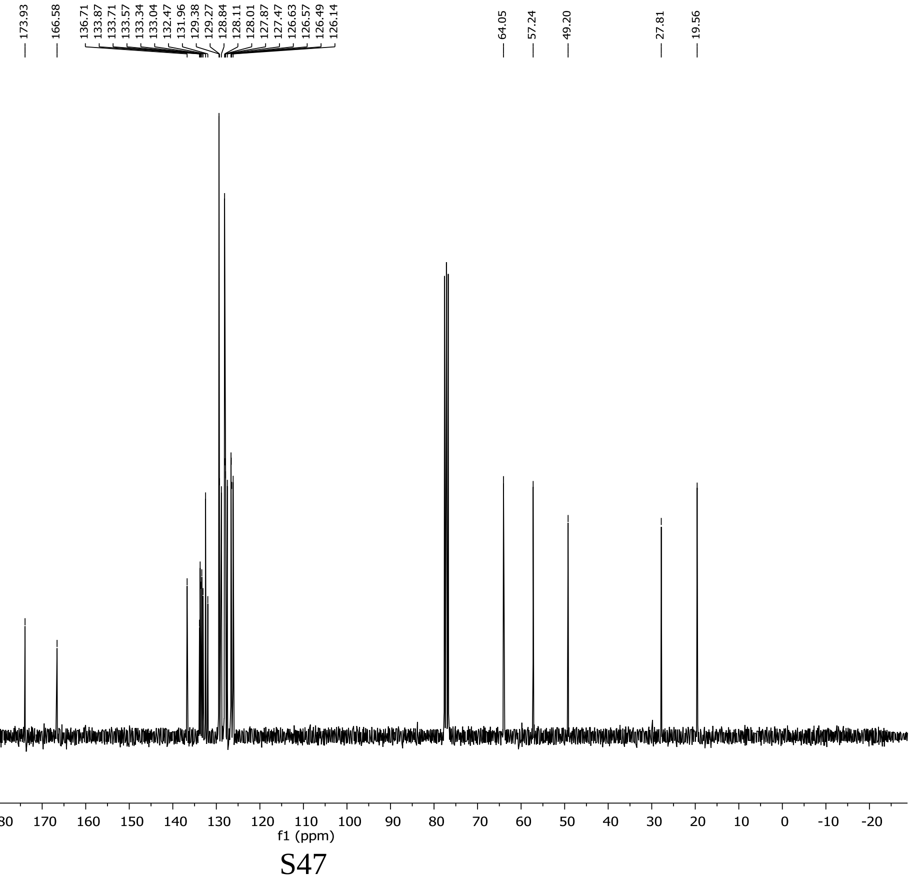

(1*S*,10b*R*)-3-Benzoyl-9-methoxy-1-phenyl-1,5,6,10b-tetrahydropyrazolo[5,1-*a*]isoquinolin-2(3*H*)-one (3da)

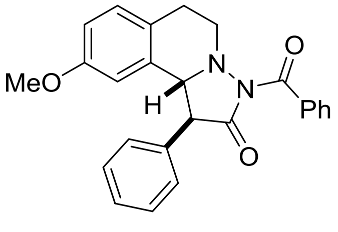

3da

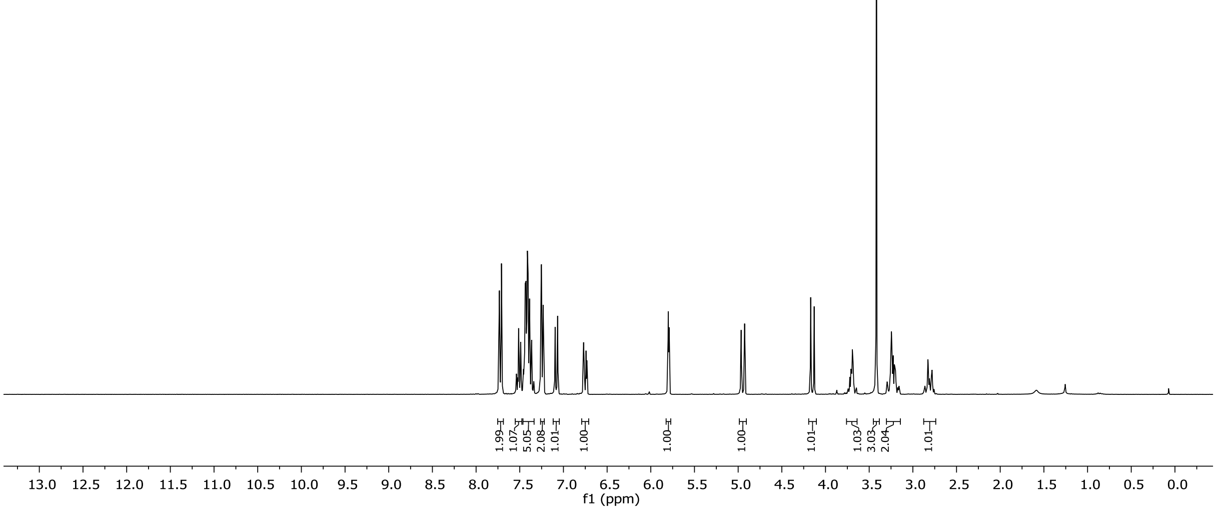

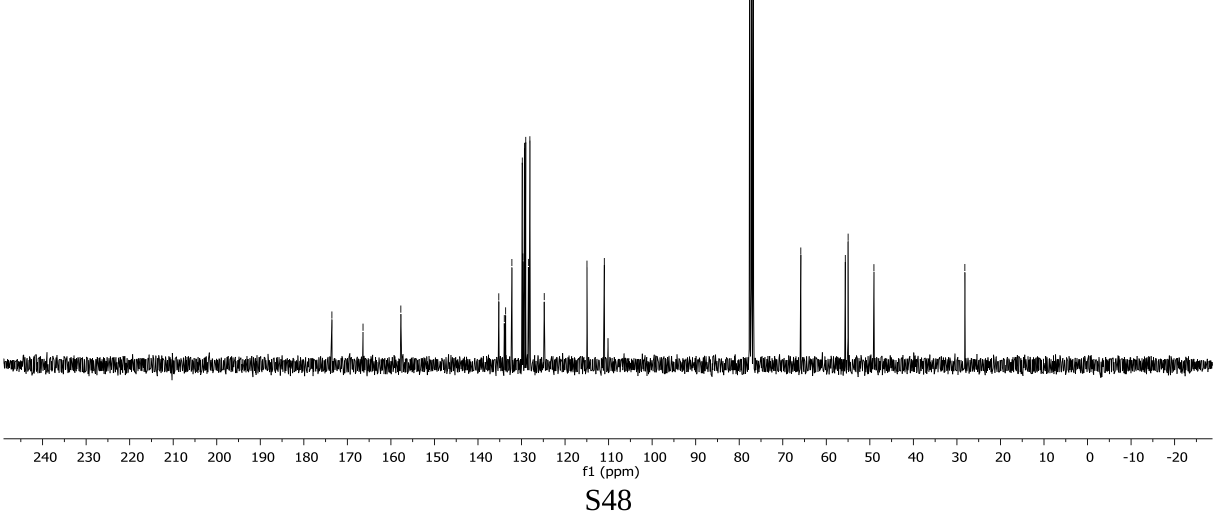

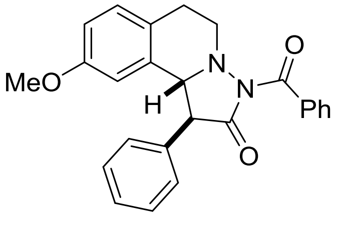

3da

(1*S*,10b*R*)-3-Benzoyl-9-methoxy-1-(4-(trifluoromethyl)phenyl)-1,5,6,10b-tetrahydropyrazolo[5,1-*a*]isoquinolin-2(3*H*)-one (3dg)

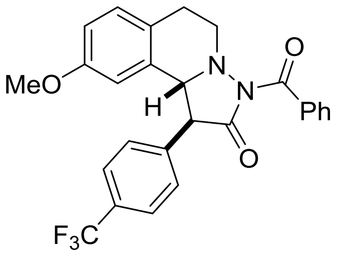

3dg

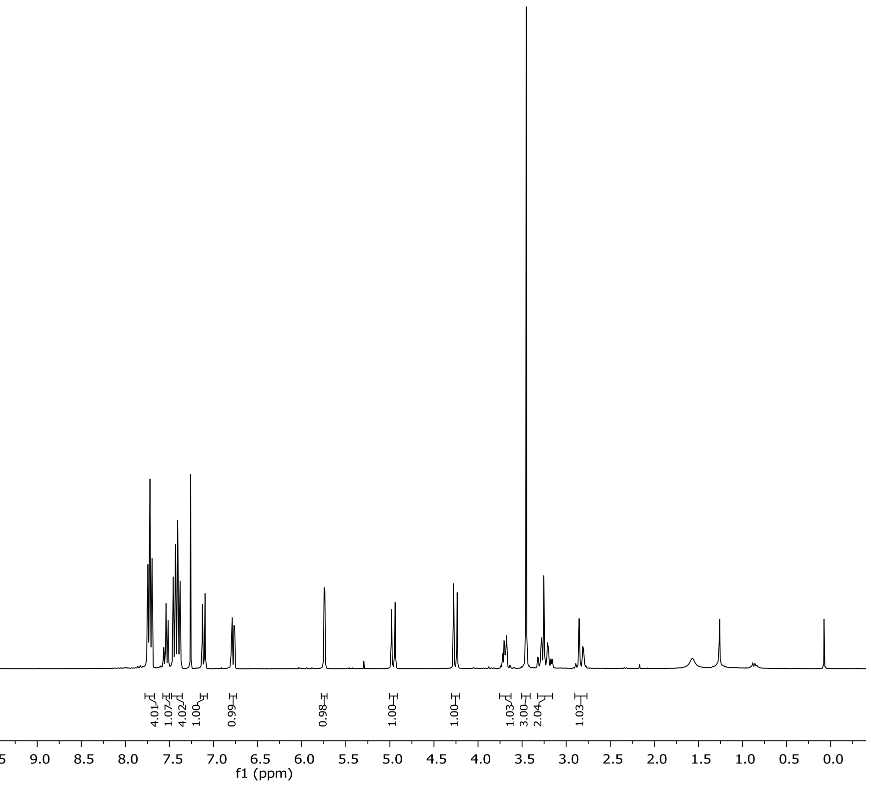

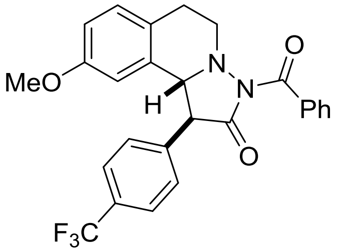

3dg

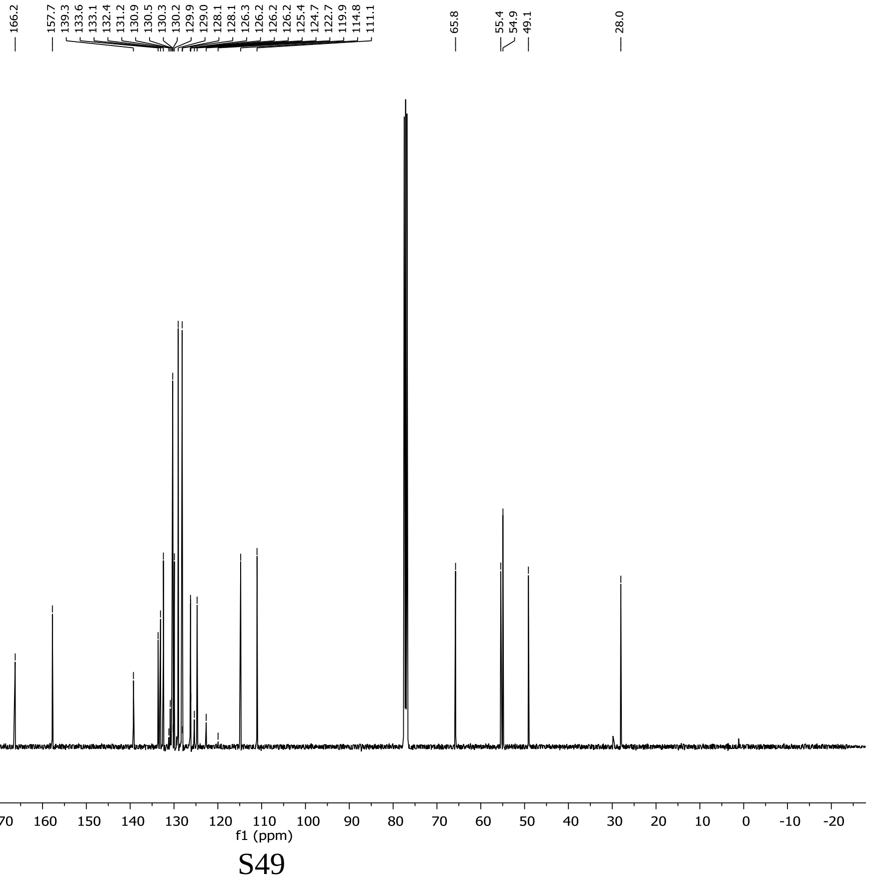

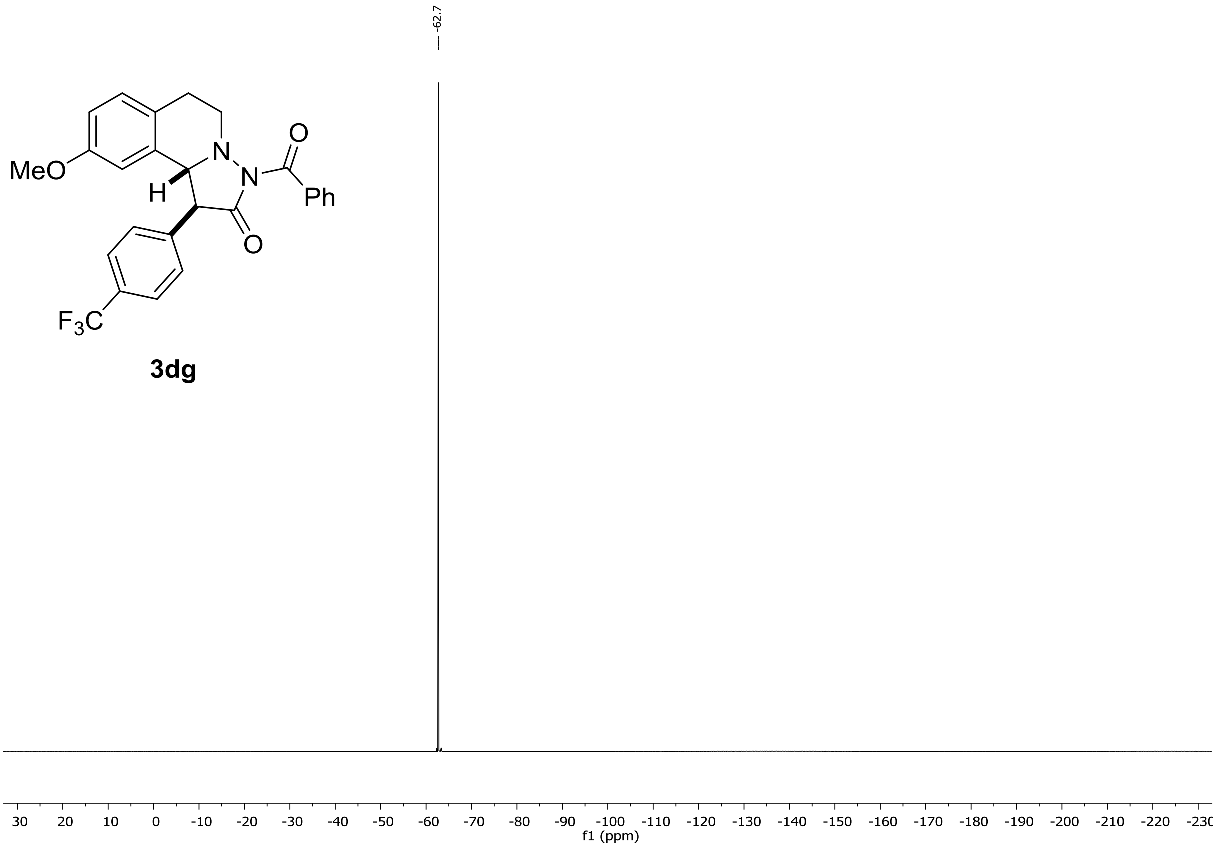

3dg

(1*S*,10b*R*)-3-Benzoyl-9-bromo-1-phenyl-1,5,6,10b-tetrahydropyrazolo[5,1-*a*]isoquinolin-2(3*H*)-one (3ea)

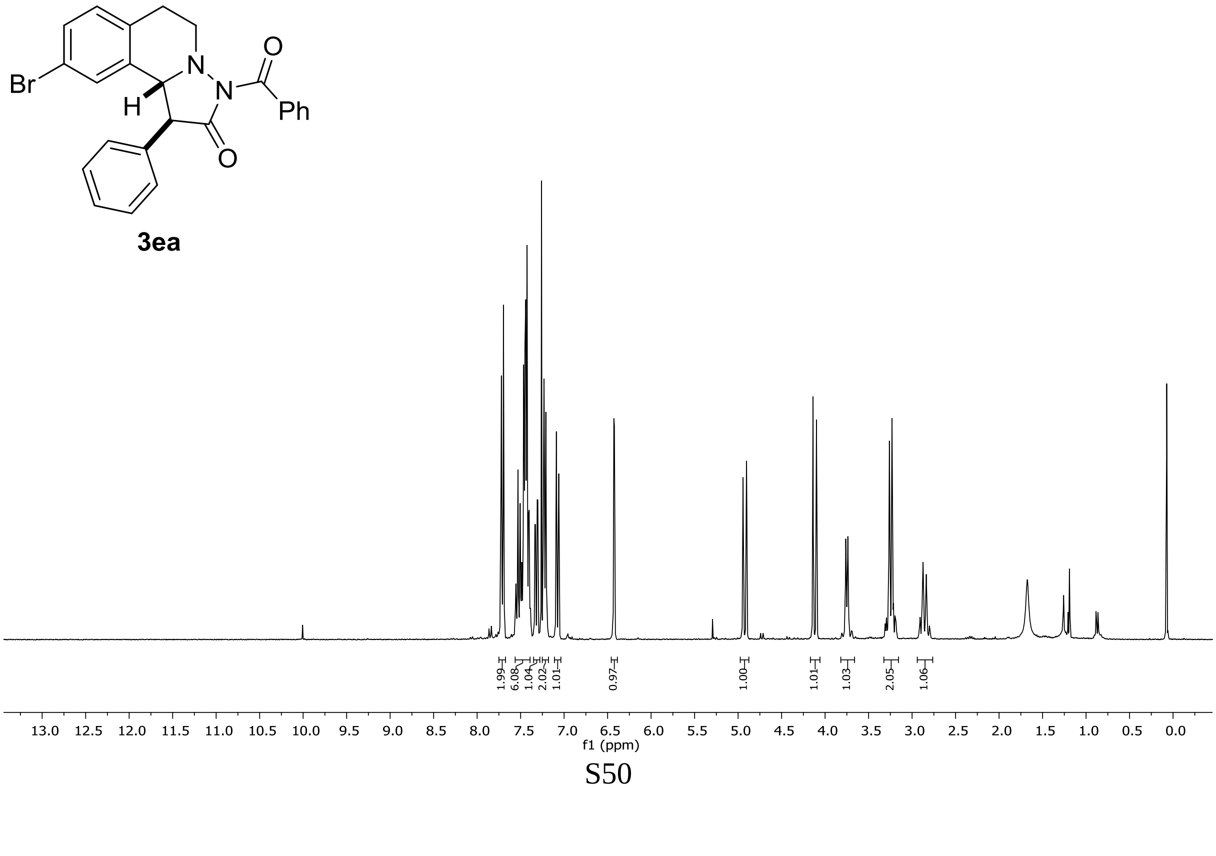

3ea

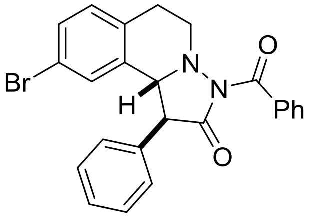

3ea

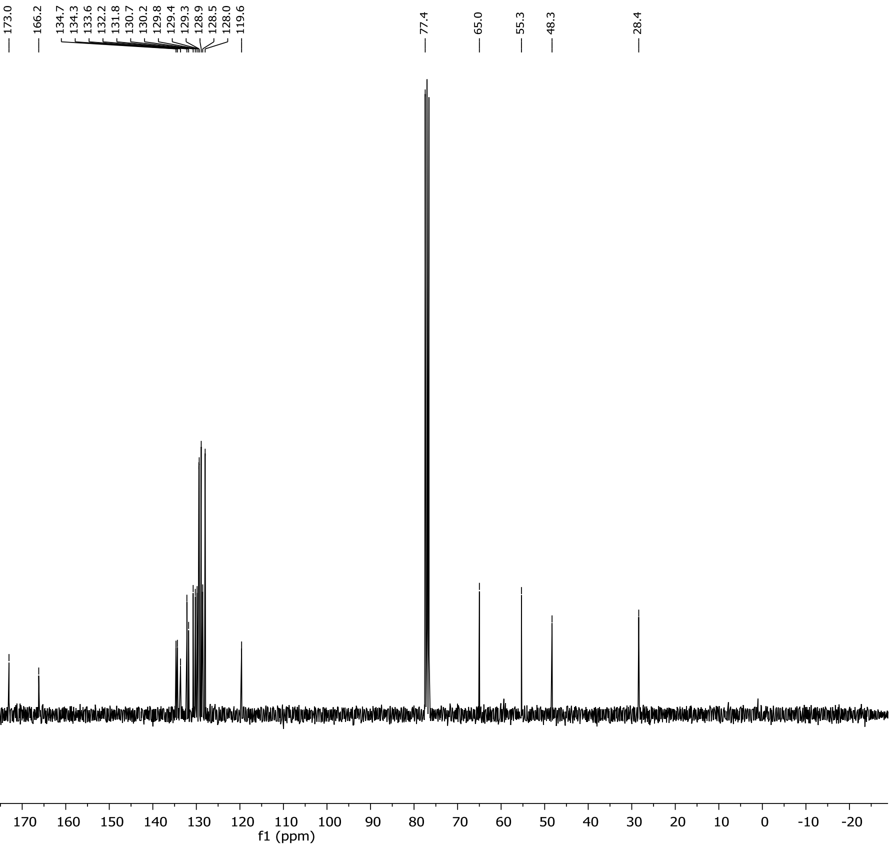

(1*S*,10b*R*)-3-Benzoyl-9-bromo-1-(naphthalen-2-yl)-1,5,6,10b-tetrahydropyrazolo[5,1-*a*]isoquinolin-2(3*H*)-one (3eh)

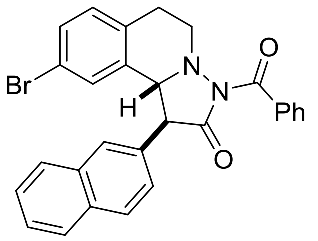

3eh

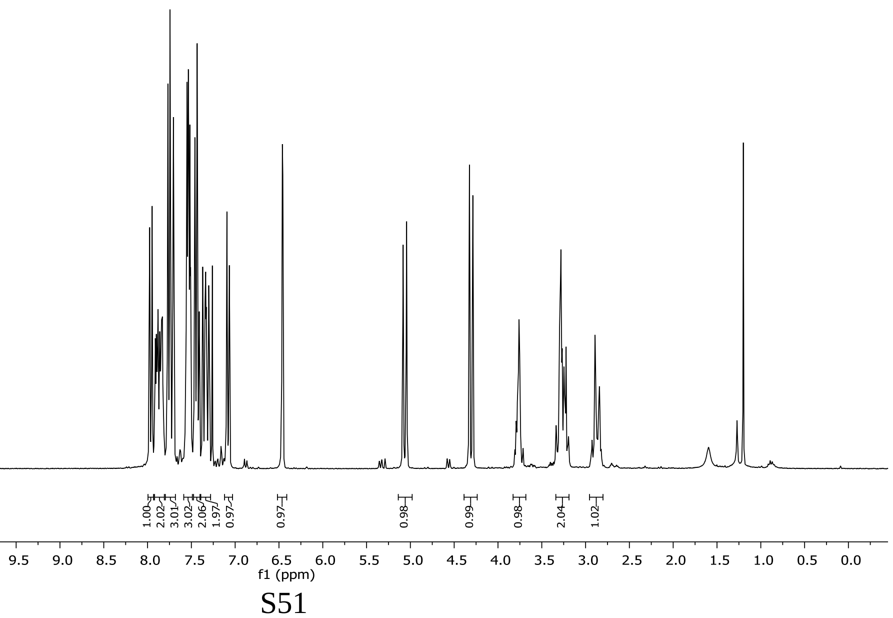

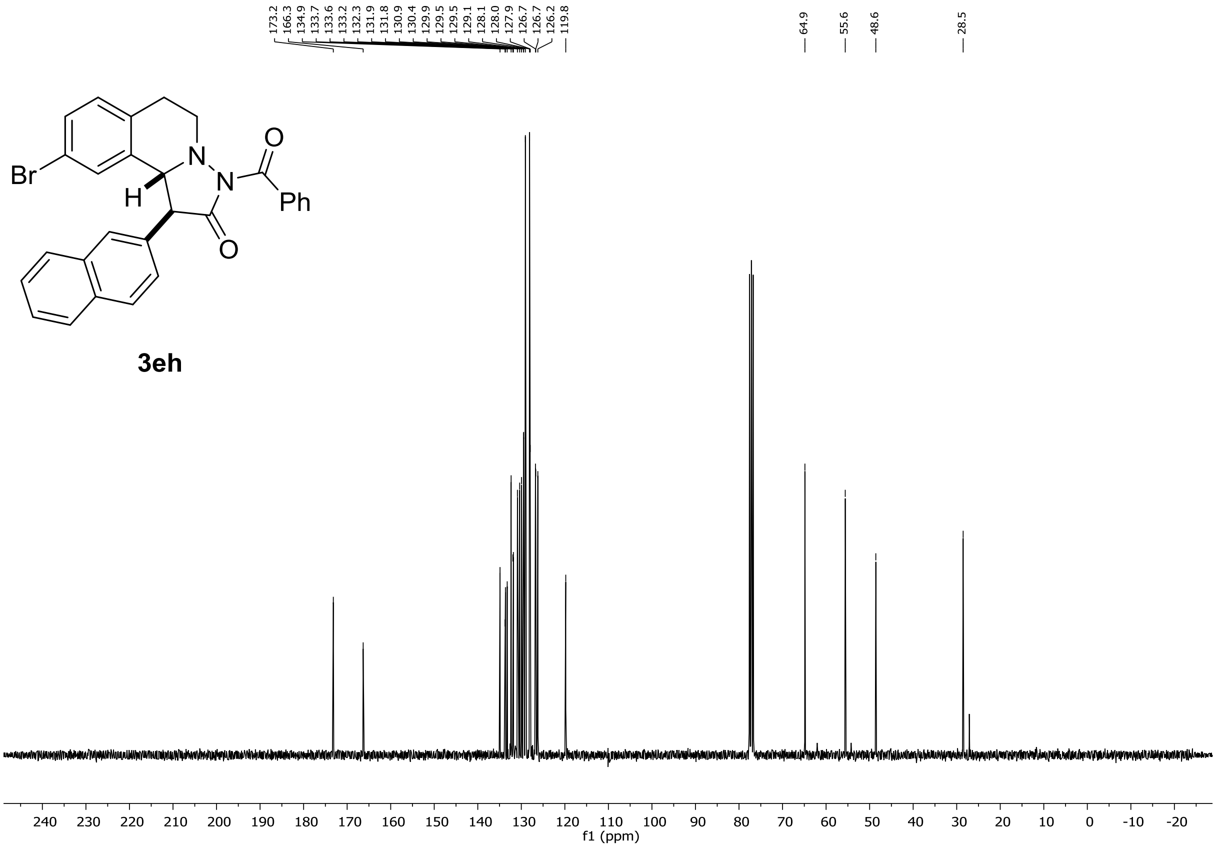

3eh

(2-Hydroxy-1-phenyl-1,5,6,10b-tetrahydropyrazolo[5,1-*a*]isoquinolin-3(2H)-yl)(phenyl)methanone (4)

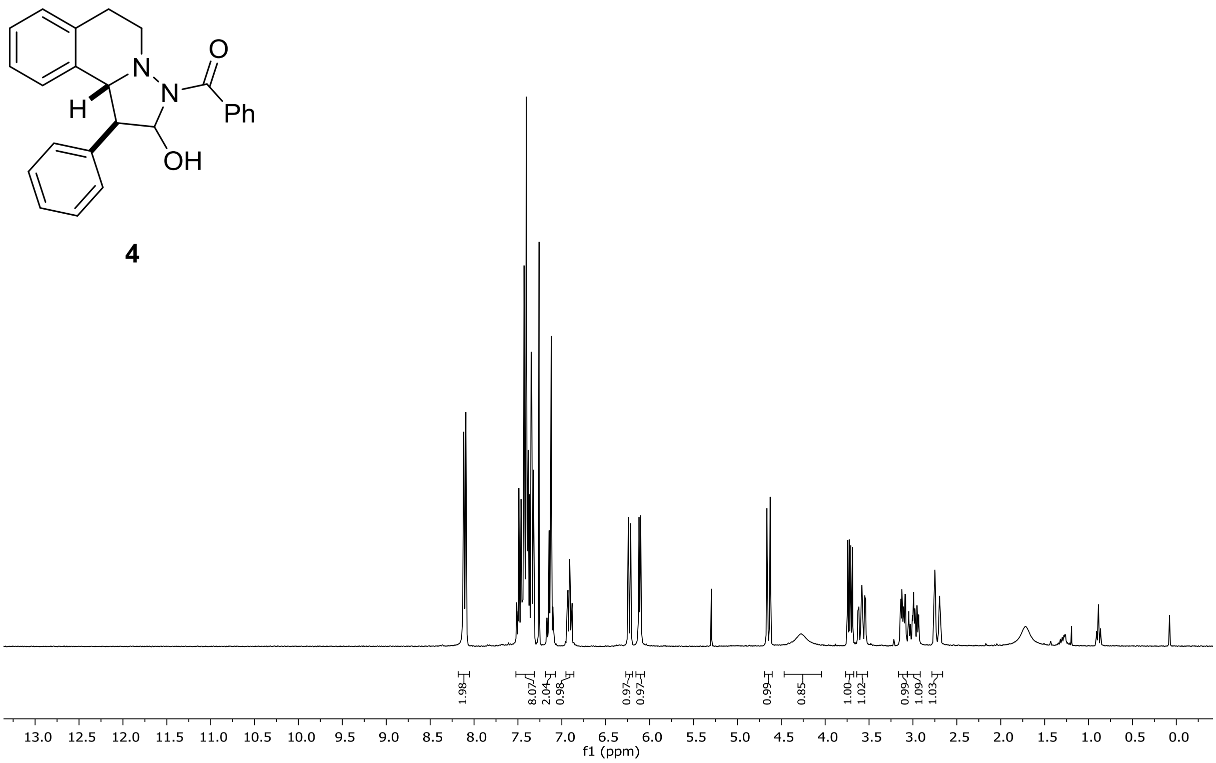

4

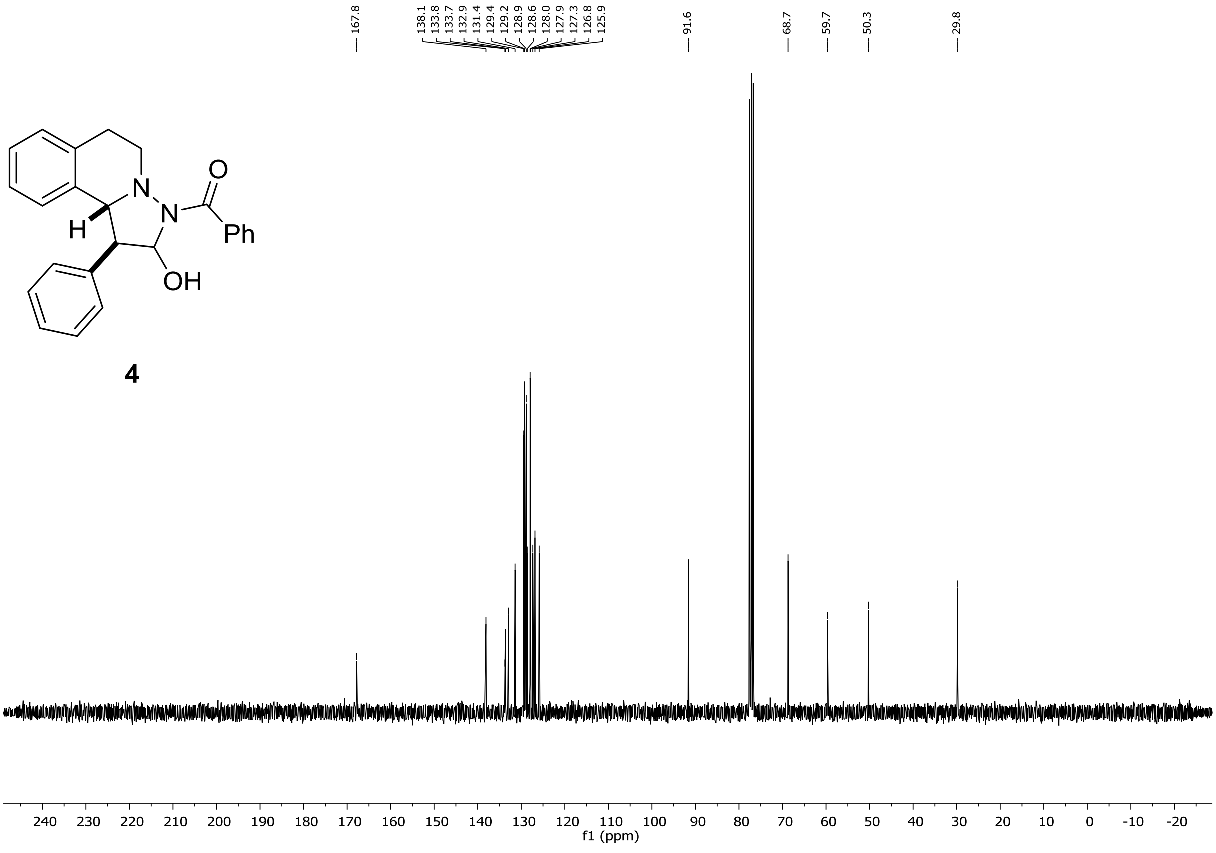

(*S*)-2-Phenyl-2-((*R*)-1,2,3,4-tetrahydroisoquinolin-1-yl)acetamide (5aa)

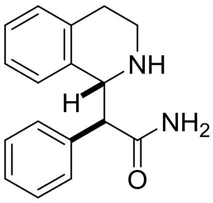

5aa

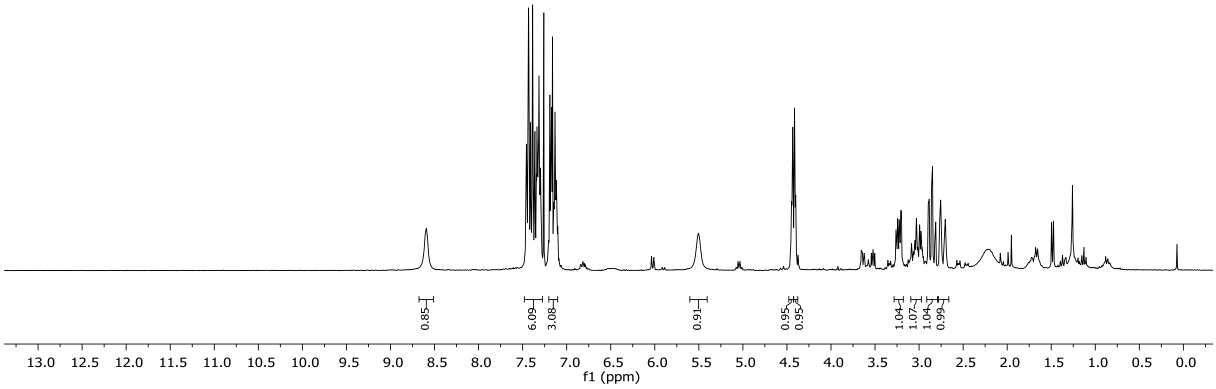

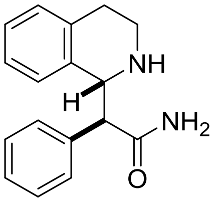

5aa

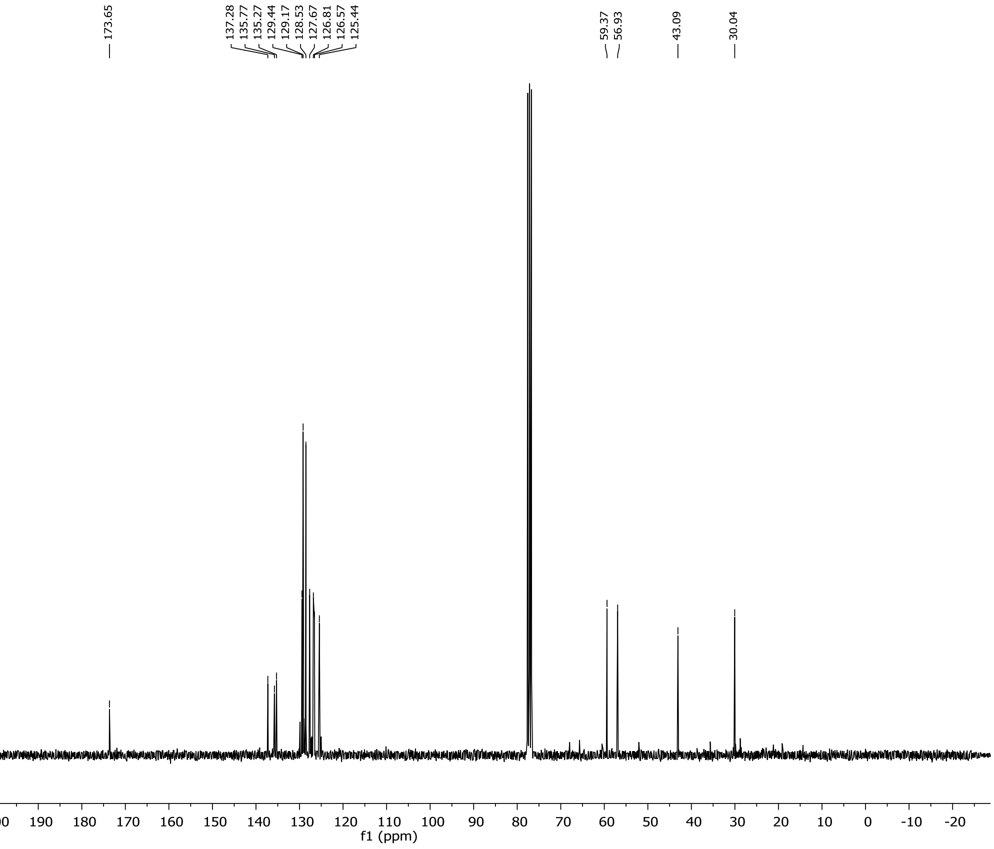

(*S*)-2-((*R*)-7-Methoxy-1,2,3,4-tetrahydroisoquinolin-1-yl)-2-phenylacetamide (5da)

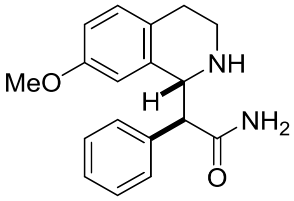

5da

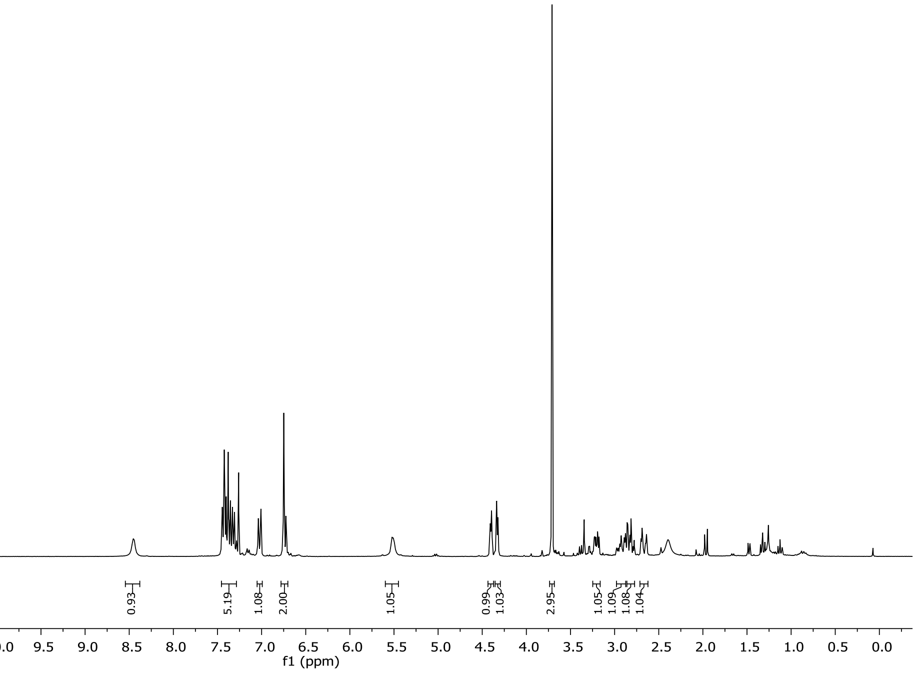

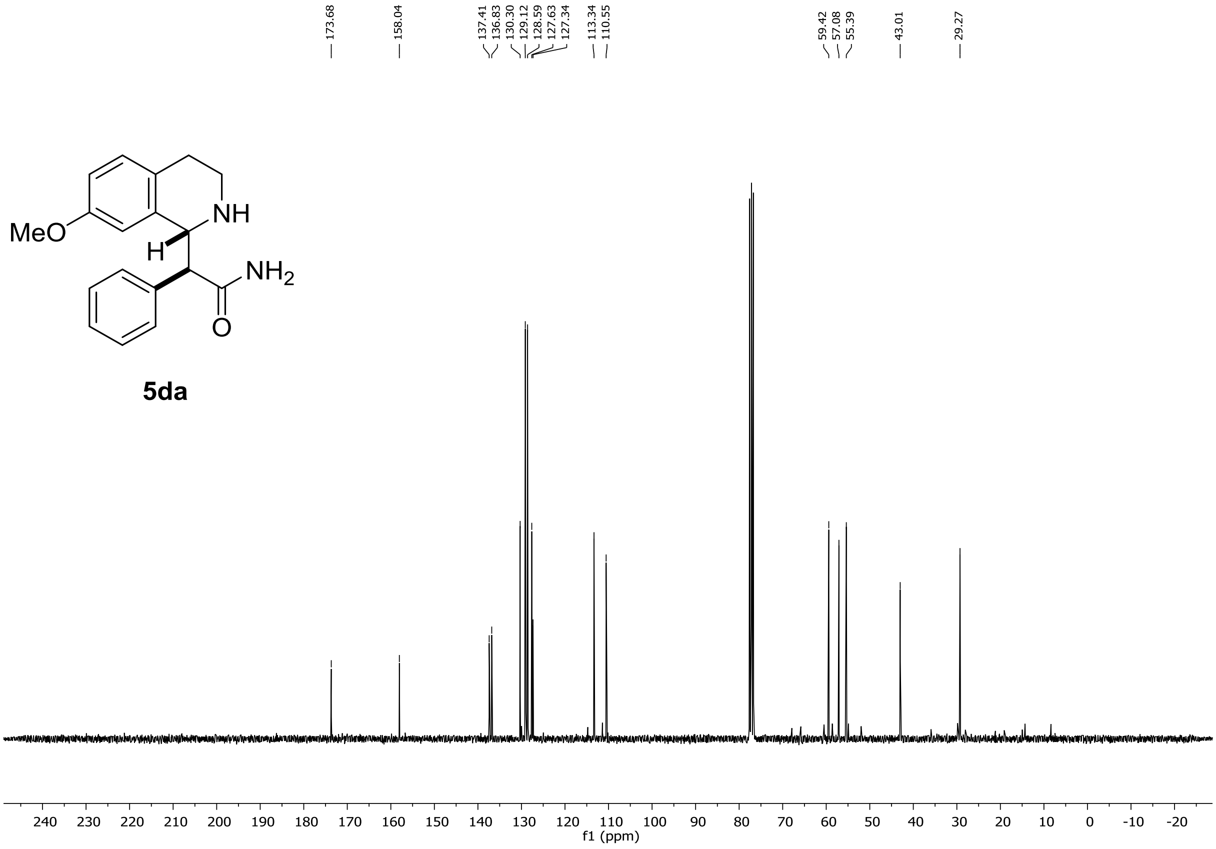
MeO
NH
H
NH2
O
5da
173.68
158.04
137.41
136.83
130.30
129.12
128.59
127.63
127.34
113.34
110.55
59.42
57.08
55.39
43.01
29.27
240 230 220 210 200 190 180 170 160 150 140 130 120 110 100 90 80 70 60 50 40 30 20 10 0 -10 -20
f1 (ppm)

7. Literature

[1] a) T. Hashimoto, Y. Maeda, M. Omote, H. Nakatsu, K. Maruoka, *J. Am. Chem. Soc.* **2010**, *132*, 4076; b) S. E. Reisman, A. G. Doyle, E. N. Jacobsen, *J. Am. Chem. Soc.* **2008**, *130*, 7198; c) P. C. B. Page, G. A. Rassias, D. Barros, A. Ardakani, B. Buckley, D. Bethell, T. A. D. Smith, A. M. Z. Slawin, *J. Org. Chem.* **2001**, *66*, 6926; d) X. Liu, B. Sun, Z. Xie, X. Qin, L. Liu, H. Lou, *J. Org. Chem.* **2013**, *78*, 3104; e) M.-Y. Zhou, S.-S. Kong, L.-Q. Zhang, M. Zhao, J.-A. Duan, Z. Ou-yang, M. Wang, *Tetrahedron Lett.* **2013**, *54*, 3962.

[2] J. L. Belletire, R. A. Bills, S. A. Shackelford, *Syn. Comm.* **2008**, *38*, 738.

[3] M. S. Kharasch, B. S. Joshi, *J. Org. Chem.* **1957**, *22*, 1439.

[4] Z. Otwinowski, W. Minor, *Methods Enzymol.* **1997**, *276*, 307.

[5] Z. Otwinowski, D. Borek, W. Majewski, W. Minor, *Acta Crystallogr. Sect. A* **2003**, *59*, 228.

[6] G. M. Sheldrick, *Acta Crystallogr. Sect. A* **1990**, *46*, 467.

[7] G. M. Sheldrick, *Acta Crystallogr. Sect. A* **2008**, *64*, 112.

[8] TURBOMOLE V6.5 2013, a development of University of Karlsruhe and Forschungszentrum Karlsruhe GmbH, 1989-2007, TURBOMOLE GmbH, since 2007; available from http://www.turbomole.com.

[9] J. Tao, J. P. Perdew, V. N. Staroverov and G. E. Scuseria, *Phys. Rev. Lett.* **2003**, *91*, 146401.

[10] F. Weigend; R. Ahlrichs. *Phys. Chem. Chem. Phys.* **2005**, *7*, 3297.

[11] S. Grimme, J. Antony, S. Ehrlich, H. Krieg, *J. Chem. Phys.* **2010**, *132*, 154104.

[12] S. Grimme, S. Ehrlich, L. Goerigk, *J. Comput. Chem.* **2011**, *32*, 1456.

[13] A. Klamt, G. Schüürmann, *J. Chem. Soc. Perkin Trans. 2* **1993**, 799.
